# Supplementary material for: Ancient DNA reveals admixture history and endogamy in the prehistoric Aegean
Source: Nat Ecol Evol. 2023 Jan 16;7(2):290–303. doi: 10.1038/s41559-022-01952-3 (PMC9911347; doi:10.1038/s41559-022-01952-3)
Supplement: Supplementary file 1 — Supplementary Note 1: Details on the archaeological background of the human skeletal material analysed for DNA, with embedded Supplementary Figs. 1–35. Note 2: Modelling and dating of genetic admixture. Note 3: Genotype imputations and pedigree reconstruction for Mygdalia individuals, with embedded Supplementary Figs. 36–38. [file 41559_2022_1952_MOESM1_ESM.pdf]

nature ecology & evolution

Article

https://doi.org/10.1038/s41559-022-01952-3

Ancient DNA reveals admixture history and endogamy in the prehistoric Aegean

In the format provided by the authors and unedited

SUPPLEMENTARY NOTE 1

Details on the archaeological background of the human skeletal material analysed for DNA.

In this section, we present extended information regarding the archaeological context from which the human skeletal remains mentioned in this study were recovered. New data from direct radiocarbon dates on selected human skeletal elements are included, as well.

CRETE

The Neolithic period in Crete

The Neolithic period in Crete has not received the attention that other periods of prehistoric Crete have to date. The reason for this is the lack of full publication of newly excavated sites. There are, however, several publications presenting material from surveys or sites which are partly excavated and published in preliminary reports. In addition, a number of papers concentrate on theoretical interpretations of existing data. Well-stratified evidence from settlements is not plentiful. Most of the existing settlement material comes from the sites of Knossos and Phaistos.

Currently, the earliest known Neolithic settlement in Crete was discovered at Knossos during the excavations of Sir Arthur Evans and later in the excavations of John D. Evans. This old material has been only partially published. A re-appraisal of the Evans material and its relation to finds from the rest of Crete has been published (Tomkins, 2018), taking into account previous publications (Evans, 1901; Evans, 1921; Evans, 1928; Evans, 1964; Mackenzie, 1903; Warren et al., 1968; Tomkins, 2007).

More recently, stratigraphic tests in the Neolithic levels of Knossos have produced stratified archaeological and palaeoenvironmental material (Efstratiou et al., 2013). A series of C14 dates, together with the dates from John D. Evans' excavations, give us a chronological framework into which pottery, architecture and artifact typology can be fitted, starting ca. 7000 BC. Recently, the C14 dates from Knossos have been re-evaluated, and the implications for their relation to other Neolithic sites have been studied (Douka et al., 2017).

The second main Cretan site which has produced Neolithic material, though of a late date, is that of Phaistos, excavated by the Italian School of Archaeology at Athens and later studied by Lucia Vagnetti (Vagnetti, 1972-1973). Neolithic material has also been found in later stratigraphic tests. In a recent appraisal of the Phaistos evidence (Todaro and Di Tonto, 2008), the Phaistos material is dated at present to two stratigraphic phases, Final Neolithic III and Final Neolithic IV (but see also (Tomkins, 2018)).

At Katsambas, not far from the northern coast, Stylianos Alexiou has excavated an important Neolithic house, at present dated to Early Neolithic I and II or alternatively to Late Neolithic I and II, with burials in cave-like tombs (Alexiou, 1953; Galanidou and Manteli, 2008). In recent years, Neolithic material and human bones have been found in a building plot at Katsambas not far from the house excavated by Alexiou (Serpetsidaki, 2011; Tomkins, 2012). Several underground cavities containing human bones and pottery were excavated. They were independent of excavated houses. A preliminary dating to Early Neolithic I and Early Neolithic II is given by the excavator. The material found in these cavities is very similar to that discovered near the Kastambas house (Galanidou and Manteli, 2008). Architectural remnants also came to light (Serpetsidaki, 2011). Comparable underground cavities have been found at Kannia near Gortys (Kontopodi, forthcoming). Part of a Neolithic settlement has been excavated at Nerokourou, on the plain, in the area of Souda Bay (Istituto per gli studi micenei ed, 1989). Lucia Vagnetti, who has studied this material, dates it to the Final Neolithic IV [e.g., see Tomkins (2007)]. A site considered as roughly contemporary was found in a totally different landscape: remnants of a settlement were found on the defensible site of the Acropolis of Gortys (Vagnetti, 1973). If this date is correct, the difference in the location chosen for the last two sites suggests that different needs and/or conditions were operative in different parts of Crete.

Neolithic houses have been excavated at Magasa near Palaikastro (Dawkins et al., 1904-1905) and at Kaloi Limenes in eastern Messara (Vassilakis, 1987). A partly-investigated settlement dating to the end of the Final Neolithic and continuing into Early Minoan I has been unearthed at Petras in eastern Crete (Papadatos, 2008). Another Neolithic settlement site with good stratification and paleoenvironmental remains has been excavated by Athanasia Kanta in the village of Kardoulianos near Kasteli Pediada, central Crete. The material from this site is presently under study. A different type of site which has produced Final Neolithic pottery is a well at Phourni, eastern Crete. The complete or almost complete vases provide a good idea of the shapes of the period (Manteli, 1992).

Cretan caves have produced Neolithic material, but few of them have been excavated exhaustively. It has been suggested that some of them were used for habitation, burial and cult practices. Most are known from preliminary reports. The cave of Gerani near Rethymnon on the northern coast is prominent among them. The Gerani cave material possibly dates from the Middle Neolithic and may continue later in the Neolithic period (Tomkins, 2009; Tomkins, 2012; Tzedakis, 1970). Extensive excavations have taken place in the cave of Pelekita, Kato Zakros. They have produced large quantities of paleoenvironmental material and C14 dates. Only preliminary reports have been published so far (Kanta et al., 2016; Bonga and Ferrence, 2016; Bonga, 2016), and the final publication is under preparation. Neolithic burials are not plentiful, though human bones and complete skeletons are known from Cretan caves (Tomkins, 2012). They all, however, have a problem of a lack of exact dating. Intramural burials are also known from Knossos (Evans, 1964; Triantaphyllou, 2008).

Aposelemis (Neolithic), Heraklion, Greece

Coordinates: 35.245350, 25.403200

Excavation: Antiquities for the Heraklion Prefecture, Hellenic Ministry of Culture and Sports, 2011-2012, directed by Dr. Athanasia Kanta (Director Emerita) and, for bioanthropological research, by Prof. Anagnostis P. Agelarakis.

Organized Neolithic cemeteries were unknown in Crete until recently. A Neolithic cemetery at Kephali in the Aposelemis Valley, central Crete, was partly located well below a large Minoan Neopalatial building and extended beyond it (Agelarakis and Kanta, 2016; Agelarakis and Kanta 2020). The cemetery was mostly comprised of graves dug into the natural soft bedrock. They varied in dimensions from ca. 1.30 by 1.20m and had a depth of ca. 0.50 to 0.65m, although there were graves of even smaller dimensions. Stones (and possibly earth) were placed on top of the bodies and covered the graves. There were also indications of burial customs, as some graves contained charcoal and animal bones, as well as small vases and some stone tools. All skeletons were placed in tightly flexed positions. There were also hearths and pits containing pottery. It seems, at present, that the pits did not contain human bones, but they had pottery, artifacts and animal bones and seem to have been related to burial ceremonies. No clear evidence of a Neolithic settlement has been found on Kephali hill. The duration of the cemetery is under consideration at present, but it does not seem to have lasted for centuries. A final answer to this will have to await the completion of the study of the material. The collagen

C14 dates from four Aposelemis skeletons are of particular importance because they provide crucial information about Crete at this time. For views on the beginning of the Neolithic in Crete, see Tomkins (2018); Douka et al. (2017).

Dento-skeletal specimens for archaeogenetic analyses were collected through random and selective sampling techniques which based on a project protocol were aiming to target a representation of different aspects of archaeological/funerary data, combined with assessments derived from the demographic/palaeopathological profiles of the skeletal population involved. Therefore, emphasis was placed on matters of: 1. the construction, spatial distribution and axonometric contextual relations of burial features, between the tightly clustered and among the relatively adjacently or peripherally positioned interments, 2. their respective relatedness to associated features of ritual function, 3. the presence or lack of associated burial artifacts, ecofacts, and manuports, 4. the age subgroups and biological sex categories, 5. the documented discrete non-metric traits of dental and skeletal variability, 6. the assessed skeleto-anatomic manifestations of developmental growth, along with acquired markers of habitual and/or occupational stress, and 7. the diagnosed dento-skeletal changes afforded by a gamut of pathological and traumatic conditions. An inherent rule of the sampling protocol dictated the absolute care and most careful handling and preservation of the archaeo-anthropological record (Agelarakis, 1996; Agelarakis, 2014); hence, no dental or relative skeletal samples were extracted which would have caused anatomic or similar structural damage to the human remains. The entirety of the materials retrieved through the random and selective sampling of specimens were already anatomically disarticulated and taphonomically, yet carefully, identified as mending components of the specific parent skeletons involved.

In total, 41 individuals from the Neolithic cemetery of Aposelemis were sampled for aDNA. Samples from the following six individuals produced genome-wide data that are presented in the genetic analyses. In addition, we present direct radiocarbon dates for four of them:

- APO004 (Grave 55-Burial 67; Lab 2014-2) is a female individual of estimated age ca. 65 years buried in a simple pit grave in a flexed position, lying on the left body side and in an east-northeast to west-northwest orientation (Supplementary Figures 1, 2). One small stone axe was found in the burial. In addition, the burial is also associated with seven stone querns. The skeleton was completely preserved but partially fragmented. The individual had a gracile morphoanatomy and exhibited significant dental

pathologies and a significant cranial vault compressed fracture in an advanced stage of healing.

- APO028 (Grave 26-Burial 30; Lab 2018-1) is a female individual of estimated age range 35-50 years who was buried in a circular-like pit (maximum diameter 0.9m; depth 0.3m) in a contracted position with the skull oriented to the south. Various limited morphological manifestations suggested a female biological sex, an estimation confirmed by the DNA analysis. One open clay vase and one stone tool were also recovered from the burial. The individual had a rather gracile morphoanatomy. Due to prevalent *ante-mortem* bio-mechanical stresses on her skeleto-muscular system, the upper extremities manifested bone changes indicative of specialized kinetics associated with heavy utility of hand flexing. She also exhibited ectocranial hyperporosis with some manifestations of healing. Radiocarbon-dating of a tooth (APO028.B): 7134±25 BP, 6059-5934 cal BC (2-sigma) (ID: MAMS-45082).
- APO029 (Grave 8-Burial 16; Lab 2018-10a) is a male individual of estimated age range 31-45 years who was buried in a contracted position with the skull oriented to the west (Supplementary Figure 3). The interment was in an ovoid pit with a maximum diameter of 1.33m. Burial goods included one clay bowl with horizontal handles, two obsidian chippings, one flint chipping and two stone axes. The individual displayed a very robust morphoanatomy both on the axial skeleton and the upper/lower extremities. Some dental pathologies and a regional ectocranial hyperporosity were also identified.
- APO037 (Burial 60; Lab 2013-23) is a male individual of estimated age range 35-50 years and was probably part of a double burial (Supplementary Figure 4). The skeleton was in a contracted position with the skull oriented southwest and was possibly covered by stones. One stone tool was included. The individual had a robust morphoanatomy and exhibited dental pathologies and mandibular superficial trauma in advanced healing and ectocranial porosity. In addition, evidence of specialized kinetics in the upper extremities was identified.

  Radiocarbon-dating of a tooth (APO037.A): 7168±32 BP, 6075-5986 cal BC (95% probability), (ID: MAMS-49519, AMS, IntCal20).
- APO043 (Grave 54-Burial 66; Lab 2014-4) is a female individual of estimated age range 35-45 years buried in a shallow circular pit of 0.85m diameter, dug on the bedrock and covered by stones (Supplementary Figure 5). The skeleton was in a contracted position with the skull oriented to the west. Three stone tools were found in the burial.

The individual had a gracile morphoanatomy and displayed cranial artificial deformation. Dental pathologies and ecto-endocranial pathological changes were identified. In addition, she exhibited specialized upper-extremity kinetics. Radiocarbon-dating of a bone (APO043.A): 7012±32 BP, 5984-5803 cal BC (95% probability, 0.4% collagen) (ID: MAMS-49520, AMS, IntCal20).

- APO044 (Grave 49-Burial 62; Lab 2014-26) is a male individual of estimated age range 25-35 years buried in a shallow circular pit of 0.85m diameter, dug on the bedrock and covered by stones (Supplementary Figure 6). The skeleton was in a contracted position, and the skull was oriented to the south. One clay vase was found in the burial. The individual had a robust morphoanatomy and presented dental pathologies and a healed superficial ectocranial compressed fracture, as well as regional ectocranial hyperporosity. Radiocarbon-dating of a bone (APO044.A): 7125±33 BP, 6065-5919 cal BC (95% probability, 0.3% collagen) (ID: MAMS-49521, AMS, IntCal20).

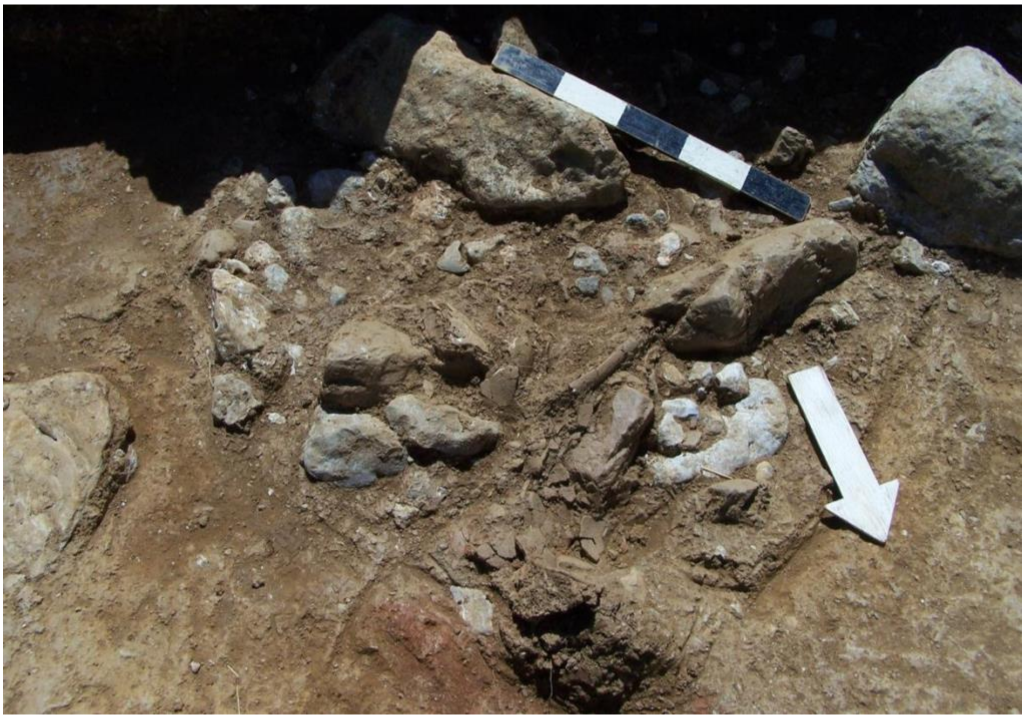

Supplementary Figure 1. Grave 55 in the Neolithic cemetery of Aposelemis.

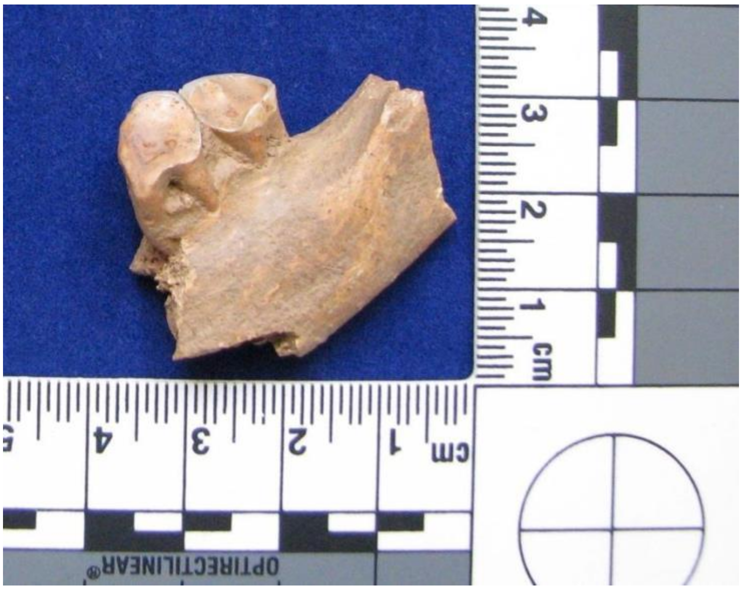

Supplementary Figure 2. Individual APO004 (Grave 55-Burial 67; Lab 2014-2): A left mandibular *corpus* fragment with the M_2 and M_3 *in situ* showing bifurcations due to alveolar bone resorption caused by advanced periodontal disease and severe, oblique wear patterns on the dental platforms. There is a nearly complete obliteration of the M_2 crown except for a remaining disto-lingual/disto-interdental enamel ring component, while the M_3 retains the periphery of its enamel ring surface where focally, buccal, supra-gingival, calculus deposits were preserved. Both masticatory platforms reveal traces of the *in vivo* reparative processes by tertiary dentin; for the M_2, nearly within the roof of the pulp chamber.

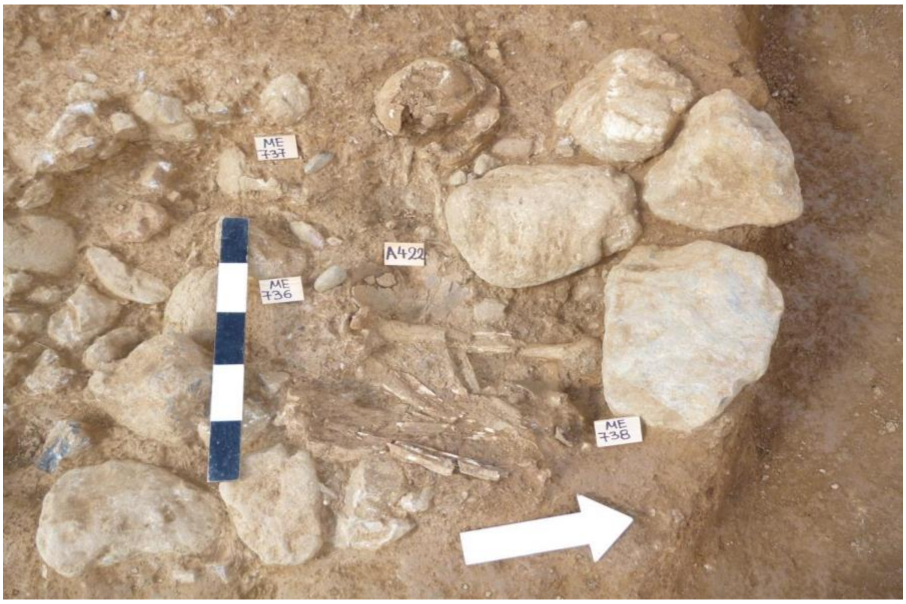

Supplementary Figure 3. Grave 8-Burial 16 in the Neolithic cemetery of Aposelemis.

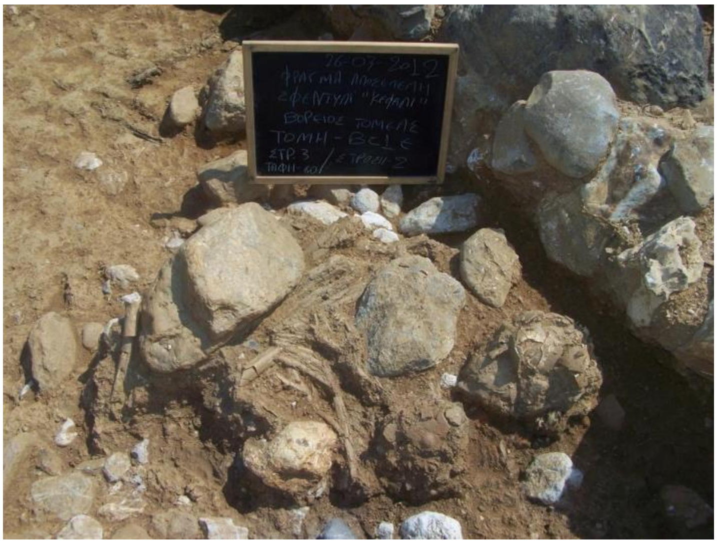

Supplementary Figure 4. Burial 60 (Northern sector; Cut B) in the Neolithic cemetery of Aposelemis.

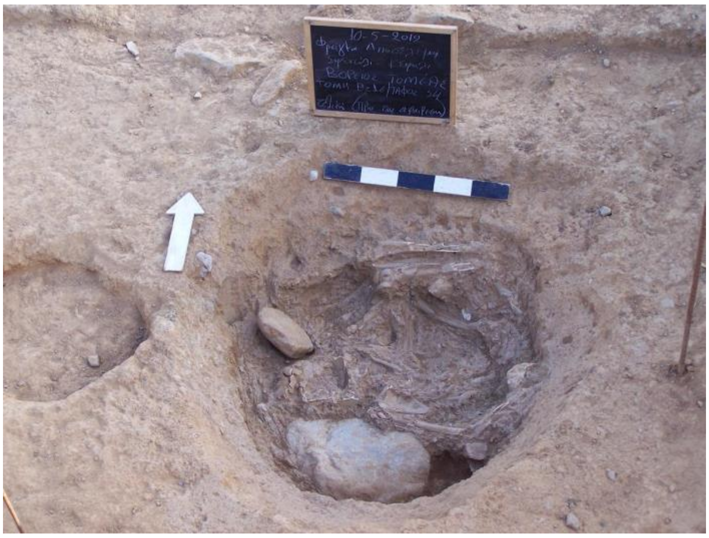

Supplementary Figure 5. Grave 54-Burial 66 in the Neolithic cemetery of Aposelemis.

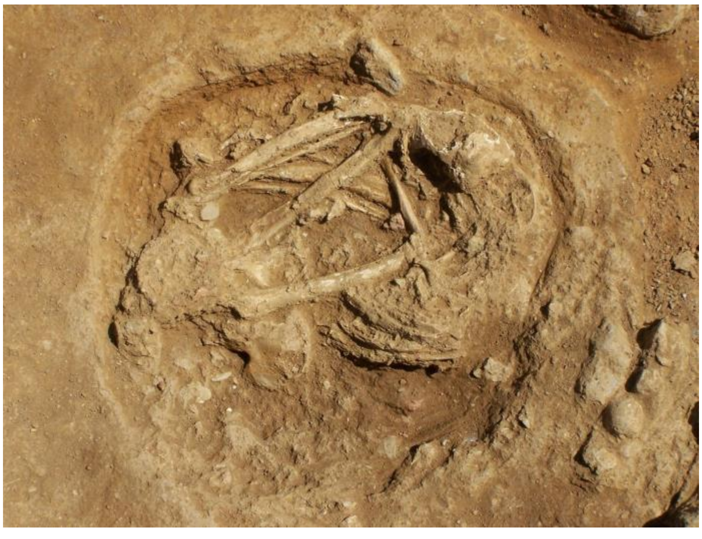

Supplementary Figure 6. Grave 49-Burial 62 in the Neolithic cemetery of Aposelemis.

Aposelemis-Ornias (Late Minoan), Heraklion, Greece

Coordinates: 35.28016123, 25.34358724

Excavation: Antiquities for the Heraklion Prefecture, Hellenic Ministry of Culture and Sports, 2007, directed by Dr. Calliope E. Galanaki; study team: Dr. Athanasia Kanta (Director Emerita), Danae Kontopodi and, for bioanthropological research, Prof. Anagnostis P. Agelarakis.

The burial cave at Ornias lies in the wider Aposelemis area. The archaeological context of this site is not very exact, as the material was collected by the Heraklion Ephorate after illicit looting activities. The site has not been published yet. Re-evaluation of the excavation data and the pottery material found in the cave suggest that the human bones collected from the cave do not belong to primary burials but represent a secondary disposal of skeletal material after a clearing of the cave for re-use. They were mostly found in a pit near the entrance of the cave. This pit contained only bones and no pottery, and direct radiocarbon dating on the skeletons (see below) produced Neopalatial dates. In addition, among the sherds collected from the cave, there were distinct Neopalatial sherds. It seems that the cave was cleared for re-use during the Late Minoan IIIA2 period and most specifically from 1350-1300 BC. Probably, the secondary disposal of skeletal material happened at this time. After the recent re-study of the pottery, the above date was confirmed. Burial caves of the rock shelter type in Crete have a long history which extends from the Early Bronze Age to the Geometric and possibly beyond. Many of these caves were used multiple times.

Following the same method for sample collection described for the Neolithic human skeletal remains from Aposelemis, samples from six individuals from the secondary burial pit were analyzed for aDNA. We present genome-wide data and direct radiocarbon dates for three of them:

- APO022 (Pit of bones at entrance of cave; Lab 2015-Dental Homo 1) is an individual of estimated age range 17-25 years. The limited skeletal preservation indicated a female, an estimation confirmed by the DNA analysis. One linear enamel hypoplastic (LEH) defect, as well as other dentoalveolar pathologies, was detected. Radiocarbon-dating of a tooth (APO022.A): 3286±21 BP, 1612-1506 cal BC (95% probability), (ID: MAMS-47520, AMS, IntCal20).

- APO023 (Pit of bones at entrance of cave; Lab 2015-Dental Homo 2) is an individual of estimated age range 35-55 years. The limited skeletal preservation indicated a female, an estimation confirmed by the DNA analysis. Advanced periodontal disease and other dentoalveolar pathologies were detected. Radiocarbon-dating of a tooth (APO023.A): 3287±21 BP, 1612-1506 cal BC (95% probability), (ID: MAMS-47521, AMS, IntCal20).
- APO025 (Pit of bones at entrance of cave; Lab 2015-Dental Homo 3) is an individual of estimated age range 35-45 years. The limited skeletal preservation indicated a female, an estimation confirmed by the DNA analysis. Very advanced periodontal disease, linear enamel hypoplastic (LEH) defect and other dentoalveolar pathologies were detected. Radiocarbon-dating of a tooth (APO025.A): 3315±24 BP, 1627-1509 cal BC (95% probability), (ID: MAMS-47522, AMS, IntCal20).

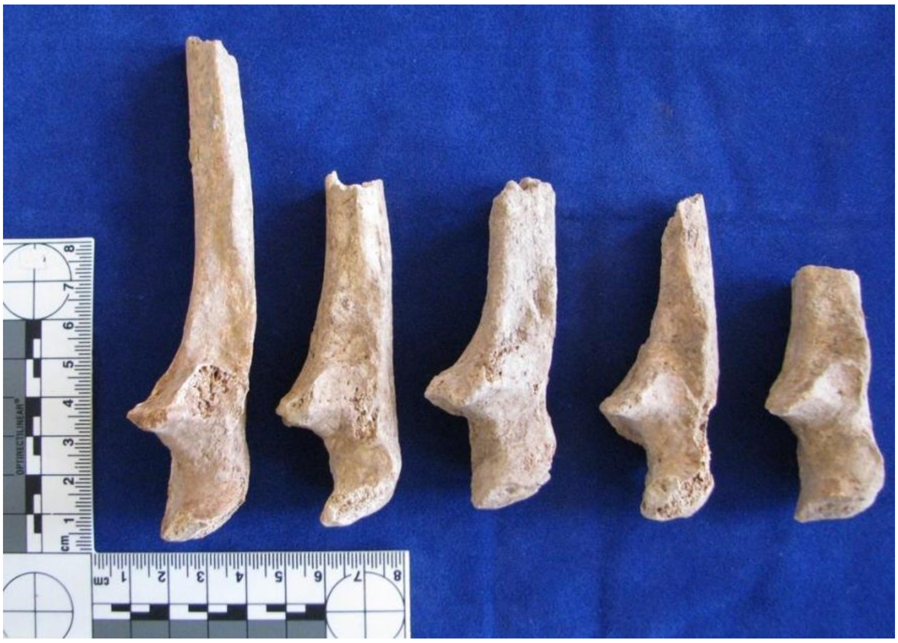

Supplementary Figure 7. Five proximal ulnar components of right forearms, identifying five individuals, within the ossuary at Aposelemis-Ornias.

Chania/Khania (ancient Kydonia), Chania/Khania, Greece

Coordinates: 35.5172563, 24.0149291 (Supplementary Figure 8)

Excavations: 25th Ephorate of Prehistoric and Classical Antiquities (-2014) and Ephorate of Khania Antiquities (2015-present), directed by Dr. Maria Andreadaki-Vlazaki (except for Palama Street: co-directed by Dr. Elpida Hadjidaki; Malefakis plot: co-directed by Ms

Efthymia Kataki; Tsapakis plot: directed by Ms Eftychia Protopapadaki). Bioanthropological research: Dr. P.J.P. McGeorge

In the 4th millennium BC, Kastelli hill in Khania (Kydonia), which dominates a natural harbour, was chosen as the most advantageous location for the first organized settlement in the area. A Minoan palace was later founded on the same site overlooking the harbour, which favoured contacts with the mainland. Contacts with the Peloponnese began in the Early Minoan period, if not earlier, and continued throughout Antiquity. A reciprocal flow of influence and merging of Minoan and Mycenaean cultural elements is evident in Khania in the Neopalatial period (ca. 1700-1450 BC).

After the palatial complex at Kydonia was destroyed in 1450 BC, a new palace was erected in LM IIIA1/A2 (ca. 1370/1350 BC) and a Linear B-based administration was established at Khania, which seems to imply the presence of Greek speakers. Khania was a vibrant centre of commerce, evidently with a multi-lingual, multi-ethnic urban population in contact with many different areas of the Mediterranean including Sardinia, Italy, Cyprus, Syria, the Levant and Egypt. In the Final Palatial period (ca. 14th-13th BC), Kydonia, based on recent finds, appears to have been a Mycenaean palatial stronghold with hints of a military capability to defend the interests of its administration. This is suggested by spectacular weapons in the "warrior tombs" discovered in the Kouklakis plot, while the position of Khania as the Cretan port closest to the Mycenaean centres of the Peloponnese and Mainland was clearly a factor that would have facilitated immigration. The simultaneous appearance in central and western Crete of new material culture and social practices characteristic of the southern Greek Mainland raises the issue of immigration on a significant scale. Mainland influence permeated many aspects of life at this time, from writing systems and trade to palatial, residential and funerary architecture.

The foundation of a new cemetery at Kydonia in this period, in LMII-LMIII, and the adoption of new burial practices and customs reflects remarkable social change and presented the opportunity to use aDNA to substantiate whether or not the theory of immigration holds true. The extramural cemetery, 800m to the southeast of the settlement, spread over an area 2km^2 to the southeast of the settlement, now lies under the expanding modern suburbs of the town and so has been excavated piecemeal as opportunities for research arose due to building permits required by private individuals (Andreadaki-Vlazaki, 1997; Andreadaki-Vlazaki, 2010; Andreadaki-Vlazaki, 2011; Protopapadaki, 2021). Two hundred tombs, isolated or arranged in groups, have been uncovered thus far. They belong mainly to three discrete

architectural types, previously unknown in Crete: chamber tombs of Mycenaean type (which replaced the multi-chambered Neopalatial tombs), pit-caves and shaft graves. This admixture of funerary architecture suggests the presence of people from different regions. Marked on the map (Supplementary Figure 8) are 9 sites from which the samples submitted for aDNA analysis derive.

The recently discovered Kouklakis plot (no. 7) is very significant for the history of Khania at the transition from the 15th to the 14th century BC, because a number of “warrior graves” furnished with bronze weapons were discovered there. Most of the burials are in “pit-caves,” consisting of a narrow trench about 3m deep with a cave-like opening in one long side to house a single burial. There are three exceptional cases with side chambers that face each other. In Crete, pit-caves had previously only been found at Zafer Papoura near Knossos, the island’s dominant Minoan palatial centre. Among the Kouklakis tombs, two examples stand out: the LM II pit-cave no. 40 and the LM IIIA1 Mainland-type shaft grave no. 46. The swords of Type C1 and D found in the tombs, suggestive of high-ranking warriors, are closely comparable with tombs at Knossos and the Argolid, and support the hypothesis of Mycenaean domination of the island during the second half of the 15th century. The new architectural tomb types, mortuary practices and grave furnishings (the weapons, clay alabastra and the three-handled piriform jars or amphoriskoi), as well as the overt warlike character of the tombs, suggest Mycenaean elements (Andreadaki-Vlazaki, 2022b).

In the Postpalatial period, after 1200 BC, following disturbances and destructions, the town was abandoned. However, the evidence provided by the cemetery indicates that some inhabitants remained in this location, implying uninterrupted occupation on Kastelli hill.

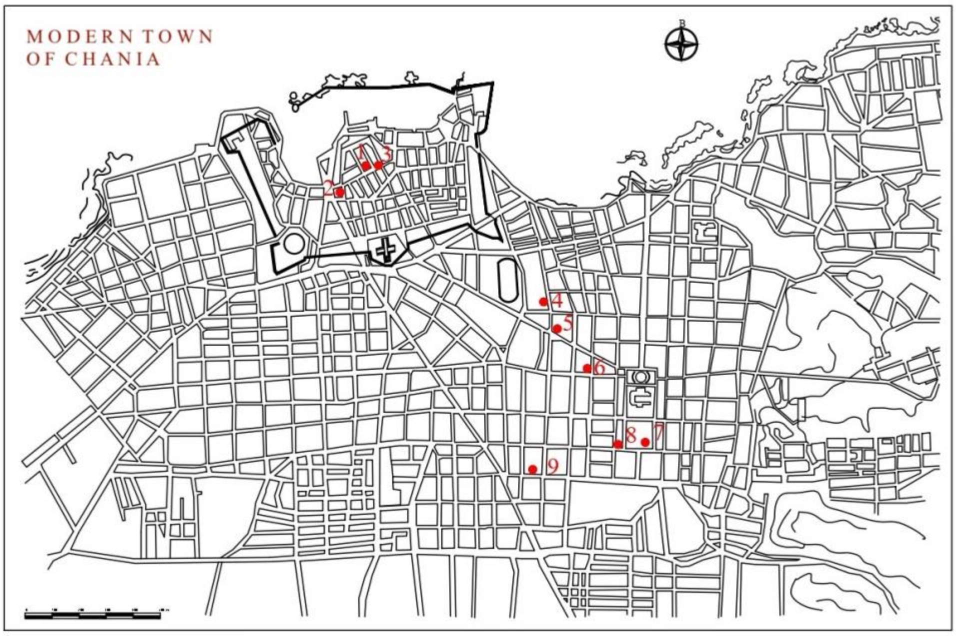

Supplementary Figure 8. Google map view of modern city of Khania with the locations of mentioned excavations. 1. Agia Aikaterini Square, 2. Katre Street, 3. Kanevaro Street, 4. Palama Street, 5. Lentaris Plot, 6. Malefakis Plot, 7. Kouklakis Plot, 8. Tsapakis Plot, 9. Rovithakis Plot.

A total of 61 samples representing 53 individuals were analysed for aDNA. These individuals come from 3 different locations on Kastelli Hill and 6 locations under the suburbs of Khania.

Kastelli Hill (Kydonia Palatial Centre)

1. **Agia Aikaterini Square**

The collaborative Greek-Swedish-Danish systematic excavation (Hallager and Hallager, 2003, 2011 and 2016) of Agia Aikaterini Square on Kastelli Hill has unearthed a Neopalatial two-storey building and part of four others. In this building complex, a baby burial **(XAN035)** without grave offerings came to light in 2014 (McGeorge, 2017; Hallager and Andreadaki-Vlazaki, 2017) buried in a shallow oval pit, 50cm long by 30cm wide and 9 to 14cm deep, covered by three stone slabs, below the LM IIIA2/B1 building 2, which in this part of the excavation was constructed deep into the Neopalatial layers. It is securely dated stratigraphically to the MM III/LM I (ca. 1700-1450 BC) and at present is the earliest

occurrence of an intramural subfloor pit burial in western Crete. Another sub-floor pit burial of a premature infant was previously discovered under an LM IIIB2 floor near the hearth in Room E used for the preparation of food (McGeorge 2003; Persson 2003). Numerous infant burials of this type have been also found at Knossos, where 16 in the Stratigraphical Museum excavation were similarly dated to MM IIIB/LM IA. Intramural burial of infants is not a typical Minoan burial custom; it was practised on the Greek Mainland throughout the Bronze Age. For example, at Asine in the Argolid, there were 57 intramural infant or child burials, 45 of them in pits (Nordquist, 1987; McGeorge, 2011; McGeorge, 2012).

In conclusion, considering the geographic proximity of western Crete to the Peloponnese, the introduction of this burial rite in Crete could be related to a subtle migration process at the height of the island's prestige and prosperity, which initiated a gradual transformation of the local population's social and genetic matrix earlier than was previously expected.

- XAN035 is a full-term infant consisting of cranial fragments including the petrous bones, long bones, ribs, metacarpals, metatarsals, manual and pedal phalanges and 12 partially-formed, unerupted deciduous teeth, which appear malformed. Since enamel hypoplasia is a correlate of low-birth-weight infants, this was probably a low birth weight, full-term infant. Lesions on the internal surfaces of the parietal and occipital bone may have been caused by bacterial infection or foetal distress due to poor maternal nutrition. These indications suggest that perinatal death may have been caused by poor maternal nutrition, poor hygiene or neonatal infection.

2. Katre Street

The ongoing Greek systematic excavation at Number 1, Katre Street on Kastelli Hill, has attracted much attention. The site lies on top of an active seismic fault, where a space of more than 140m^2, covered by a roof supported by wooden pillars in two rows, was discovered (Supplementary Figure 9). In the northern and northeastern end of the area, the lower parts of walls were covered with wall paintings preserved up to 0.65m in height (Andreadaki-Vlazaki, 2022a), reinforcing the view that the structure is part of the Mycenaean palatial building complex.

In the LM IIIB1 period (ca. 1300-1250 BC), following a catastrophic earthquake (6.5/7.5 Richter), a huge sacrifice took place in the plaster floor, part of which had been intentionally removed. The dismembered remains of a young woman (XAN036) (Supplementary Figure 10) were found mingled with a mass of similarly dismembered remains of various animals (43 sheep and wild goats, 4 pigs and 2 cattle) (Supplementary Figure 11). Her body had been treated in precisely the same manner as the animal carcasses. There was no evidence of burning (Andreadaki-Vlazaki, 2015; McGeorge, 2015; Mylona, 2015; Andreadaki-Vlazaki, 2018). The deposit was demarcated and sealed by a deposition of stones and slabs. This unique discovery of a human sacrificed in a ritual context should not come as a surprise, since Greek mythology presents numerous examples of virgins offered in purification sacrifices in exceptional circumstances. The sacrifice enacted here appears to have been intended to appease chthonic deities after the emotionally shocking devastation caused by the earthquake.

- XAN036 is a young female, whose skeletal remains had similar breakages and cut marks to the animal bones, sustained on ‘green’ or fresh bone. All the parts of the skeleton, which is far from complete (Supplementary Figure 12), clearly belonged to a single female individual of small, slight build.

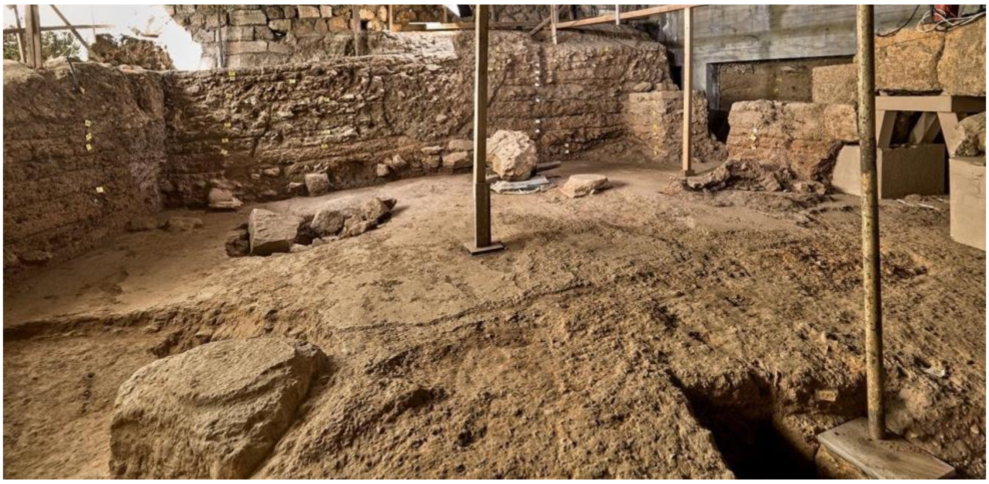

Supplementary Figure 9. Katre Street Nº1. Part of the Column Space with plaster floor.

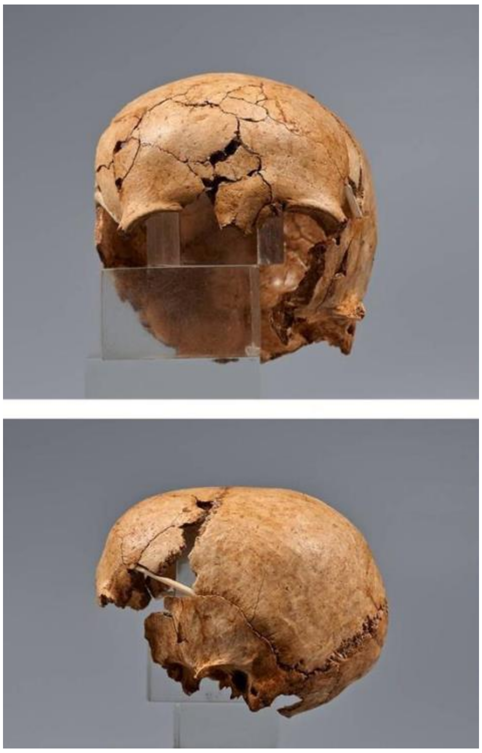

Supplementary Figure 10. Katre Street Nº 1 (XAN036). The human skull reconstituted.

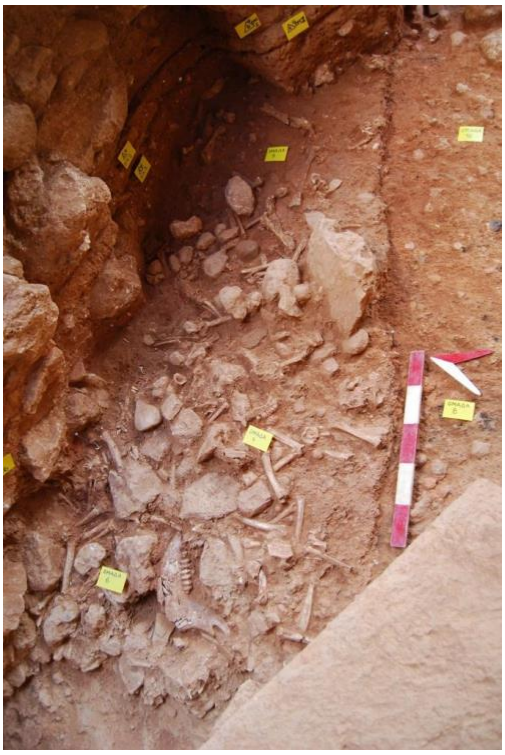

Supplementary Figure 11. Katre Street Nº 1. Detail of the deposit of bones found *in situ* on the destroyed plaster floor.

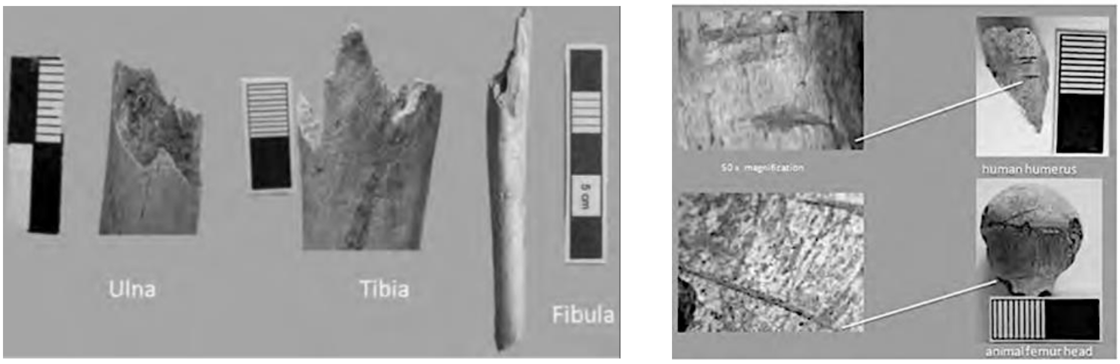

Supplementary Figure 12. Katre Street Nº 1 (XAN036). Left: W-shaped fractures, with sharp, bevelled edges. Right: Cut marks on human humerus and animal femur.

3. Kanevaro Street

In 1998, the paving of Kanevaro Street, the main road that crosses Kastelli hill from west to east, revealed building remains of various phases of the Minoan palatial settlement (Andreadaki-Vlazaki, 2004). The remains of three human skeletons, two women and a man **(XAN013, XAN014, XAN015)**, were found at different levels in a deep narrow well carved into the bedrock. The well was filled with many stones, slabs, parts of pithoid and cylindrical vessels, an *asaminthos* and a painted stirrup jar. The pottery from the well dates to the LM IIIB1 period (ca. 1300-1250 BC).

- XAN013
- XAN014
- XAN015

Kydonian Cemetery

4. Palama Street

A building plot at No. 4 Palama Street is 800m from the excavated area of the Bronze Age settlement on Kastelli Hill. A dense cluster of 17 rock-cut tombs of three different types was excavated (Supplementary Figure 13). Eleven were pit-caves (: 1-4, 8,9 and 11-15); four were

chamber tombs (: 5, 10, 16 and 17); one was a mere ‘cavity’ (:6); and, one was a simple pit grave (:7). A circular stone structure in the midst of the tombs is thought to have served some practical or ritual purpose in funeral ceremonies.

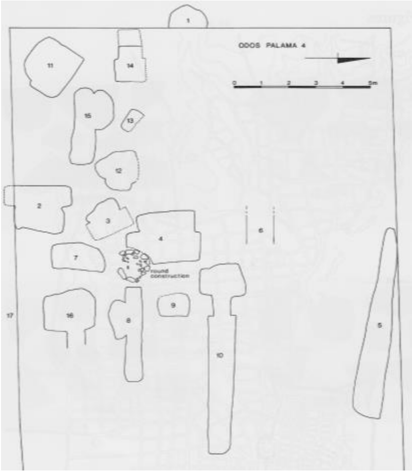

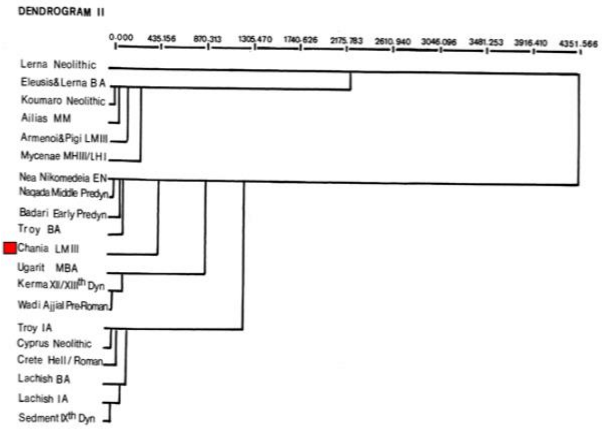

Supplementary Figure 13. Left: **The Palama St. tombs**; Right: **Cluster analysis**

The 17 tombs from Palama St. yielded a total of 29 inhumation burials, 16 adults and 13 children, of which one was an embryo and seven were under 5 years old. The remaining five children in pit cave 11 were between 6 and 11 years old and appeared to have died in a short space of time, perhaps victims of an epidemic, such as cholera, typhus, measles, plague, or of an accident or natural disaster. Two children with *cribra orbitalia* may have suffered from iron deficiency anaemia or a heavy parasite load. The mean age at death of males (n=7) was 34.14 years and of females (n=9) 25.6 years. Caries in females was10% higher, and the incidence of abscesses and the rate of tooth loss twice as high as in males. The abnormal difference in mean stature of males (164.54 cm) and females (148.83) reflects a difference in the status of men and women. Stature correlates directly with nutritional status and access to food resources, which is in turn linked to susceptibility to disease and to factors such as population density, hygiene and medicine.

The Palama Street tombs all date to the LM IIIA2-LM IIIB1 period (ca. 1350-1250 BC) (Hallager and McGeorge, 1992). None had been robbed or re-used, probably because they are not high-status and contained only modest grave gifts. Most of the 14 clay vases were locally made, but there was one imported Mycenaean piriform jar.

The most exotic and precious finds from amongst the tombs in this plot were the three “un-Aegean” silver signet rings that were found in tomb 8: one in the cave with the pregnant

female 8C **(XAN007)** and another two in the earth fill of the rectangular pit, together with the bones of a man and a woman. All three signet rings are similar in size and design and probably originated from the same geographical source. The seal devices of the two best-preserved rings present a rather crude quadruped and a human figure. A close parallel to the latter is found in a silver signet ring with the figure of the Egyptian god Bes, guardian of pregnant women and children, found at Ras Ibn Hani, the harbour town of Ugarit (Bunni et al., 1998). Although this parallel was not known at the time of the 1992 publication, the cluster analysis of morphometric cranial measurements on 20 female populations from Mainland Greece, Crete, Cyprus and the Levant had previously clustered the females from pit-caves 3 and 8 between Bronze Age Troy and Middle Bronze Age Ugarit (Supplementary Figure 13).

- XAN003 is a young adult female interred in chamber Tomb 10A with two children accompanied by a steatite and two carnelian beads, a bone pendant and a crab's claw. Fusion of the basilar suture and tooth wear place this individual within the 17–25-year age range. Her cranium was stippled with coarse porotic lesions, the aetiology of which could be thalassaemia, a quantitative disorder of the haemoglobin, or some other form of anaemia, such as anaemia associated with chronic infections. Her dental health was poor: 70% of her teeth had caries. Enamel hypoplasia indicates episodes of ill-health or poor nutrition in childhood. Pronounced development of the right humerus' deltoid muscle contrasted noticeably with the left arm, indicating right hand dominance.

- XAN007 is an undisturbed male burial without grave gifts in the pit cave Tomb 15C. He was a mature male, aged between 30-40 years of age. In the shaft, 15kg of broken pottery dated to LM IIIB1 allows a terminus *ante quem* for the burial. Like XAN003, this cranium also bore porotic lesions. Thirteen teeth had been lost ante-mortem and three *post-mortem*. The remaining 16 teeth exhibited fairly severe dental attrition, and 10 had caries. Hypoplastic lines were observed on most teeth. There were traces of plaque and evidence of alveolar recession caused by periodontal disease The teeth of the lower dental arch were crowded and misaligned. The enamel of the lower left 1st molar was fractured, probably when biting on some hard substance; over time, with usage, the damage became worn and shiny. Herniation of the discs of the mirror surfaces of thoracics 8 and 9 and severe degenerative changes in the lower thoracic and lumbar sections (Th10 to L3), rarely seen in the modern era in patients under 60, may

be linked with bending, turning or pivoting movements putting a strain on the spinal column.

5. Lentaris Plot

The E. Lentari - Ch. Manolikaki plot, in A. Papandreou Street, was excavated in 1987 (Andreadaki-Vlazaki, 1992; Preve, 2009a). Five tombs came to light: four chamber tombs and one pit cave, which was found empty. The chamber tomb burials had been disturbed by looters. The few offerings, mainly clay vessels, that were left date to the LM IIIB period (ca. 1300-1200 BC). Tomb 3 is a chamber tomb (1.70 X 2.10, ca. 1.50m with a dromos 5.50m long and 2.70m deep. In the centre of the chamber, there was a male burial in situ **(XAN016)** and the bones of at least seven other individuals in heaps and in a deep pit carved into the floor **(XAN017, XAN018)**. The material is being prepared for a detailed publication.

- XAN016 (Lentaris plot – Tomb 3 – Individual 1) was found in a crouched position in the centre of the tomb, with its head towards the entrance, and covered in debris from the roof collapse. Its damaged cranium and pelvic bones were diagnostically male. All of the molars had been lost ante-mortem. Some severely worn anterior mandibular teeth, with their pulp chambers exposed, suggest an age of 40-45 years. Linear enamel hypoplasia seen on the lateral incisors and premolars and Harris lines seen on the x-rays of the fibulae and tibia with marked periostitis indicate growth arrests. Calcified tendons were present on a right patella and left calcaneum. A mean stature of 169.77cm was estimated from the right femur and tibiae.

- XAN017 (Lentaris plot Tomb 3 – Skull 2) is the well-preserved secondary burial of a young adult female. She presents porotic hyperostosis on the parietals and squamous, wormian ossicles on the left branch of lambda and a unilateral occurrence of the left parietal notch bone. She shares these hereditary traits with 3B, to whom she is very probably related. Her dental health was very poor, having lost 10 teeth ante-mortem (including all the mandibular molars and premolars, apart from the RM_3, and pm_1). Three sockets (RM^2, Rpm_1, LI^2) had abscess infections, and there was an abscess sinus on the right cheek bone. All the teeth had significant amounts of root exposure, ranging from 3.5 to 1.8mm, indicating serious periodontal disease with inflamed gingiva.

Calculus adheres to the buccal and distal surfaces of upper RM^1. The basilar suture was fused. Clearly visible vault sutures and dental attrition indicate an age of 17-25 years. Almost the entire vertebral column is present, but only the first element of the sacrum. S1-2 centra had not fused, confirming this individual was < 27 years old. Prominent intertrochanteric lines for the ilio-femoral ligament, a well-developed deltoid ridge on the right humerus and radius pronator teres insertion indicate a physically active individual. Post-bregmatic flattening of the parietals might be due to carrying weights on the head. A mean stature of 159.93 cm was estimated from 8 long bones.

- XAN018 (Lentaris plot. Tomb 3 Skull 3C) is the well-preserved secondary burial of another young adult female, aged between 17-25 years based on suture synostosis and tooth wear and pubic symphysis morphology. Four molars had been shed ante-mortem (both lower M_1s and RM_3 and upper LM^2). There are deposits of calculus on the upper molars and premolars, with evidence of periodontal disease. RM_2 had an abscess infection. Nearly all teeth had dental hypoplasia. There is a unilateral, left side, occurrence of the epipteric bone. A small osteoma on the occipital bone near lambda, 4-5mm in diameter, about 2 mm above the vault surface. The post cranium shows a strong, young individual actively involved in manual labour. The medial surface of the tibiae presents periostitis, and the left fibula below the distal third of the shaft has a sinus for the evacuation of pus**.** Stature, estimated from 10 long bones, was 157.60cm.

6. Malefakis Plot

Two LM III chamber tombs, 1 and 3, and the dromos of a third, tomb 2, were excavated in 2008 in the Malefakis plot at the junction of I. Sphakianakis and Platon streets in the heart of the cemetery of ancient Kydonia (Kataki 2014). Tomb 1 is a subterranean, rock-cut chamber tomb (Supplementary Figure 14). The dromos is 15m long and 6.90m deep. The dimensions of the chamber are 2.90 x 3.50 x 2m. It was plundered in antiquity, like the majority of similar tombs in Khania. Bones of four skeletons were interred in the dromos **(XAN022, XAN023, XAN024**: two women and a man**)** (Supplementary Figure 15), covered with huge stones, while another skeleton of a child lay on the wall blocking the entrance, together with two LM IIIB conical cups **(XAN021)**. The chamber had traces of extensive fire and the remains of at least

one burial accompanied by four clay vases dated to the LM IIIB period, an agate sealstone and two stone conical button beads.

Tomb 3 is also a chamber tomb, plundered in antiquity. The dromos is 10m long and 6.13m deep. The dimensions of the chamber are 2.80 x 2.50 x 1.70m. The skeletal remains of a man (**XAN027**) were found in the dromos. At a depth of 4.50m, in front of the entrance to the chamber, a rectangular stone (0.56 x 0.25m) may have been a grave marker. The upper part of the blocking wall of the chamber had been removed by looters. A small part of the roof had collapsed into the chamber, and bones from at least four skeletons were found around its rectangular perimeter, though none was in situ (Supplementary Figure 16). Female skeleton 2 **(XAN025)** was found in a rectangular niche in the northwestern corner of the chamber. In addition to the niche, there are two other shallow pits in the southeastern part of the chamber. The bones of skeleton 3 **(XAN026)** were piled, together with the bones of the fourth skeleton, along the western side of the chamber. Owing to the looting, only sherds dated to LM IIIB, a sealstone and two steatite beads were found. The material is being prepared for a detailed publication.

- XAN021 (Malefakis plot – Tomb 1 – Individual 1**)** is a female child aged between 8 and 10 years old represented by a fragmented cranium, mixed dentition (Supplementary Figure 14) and incomplete postcranial remains. The child's unerupted RC^1 and RM^2 exhibit linear enamel hypoplasia, evidence of stress possibly due to malnutrition or childhood illness.

- XAN022 (Malefakis plot – Tomb 1 – Individual 2; Supplementary Figure 15) is the young adult female skeleton uncovered in the dromos at a depth of 3.05 - 3.16m, about 50 cm below burial 1, with sherds from an amphoroid krater, a kylix and the shaft of a bronze needle. She lay supine, arms by her sides, head to the southwest, towards the chamber, facing west where a boulder was positioned. Her cranial table, in numerous pieces, was thin. There were 28 teeth with maxilla and mandible fragments. Both mandibular 2nd molars appear to have been lost not long before death, as the alveoli had just begun to heal. The upper lateral incisors were lost *post-mortem*. There were 5 cavities. The upper right M^1 had a large interdental cavity, while the upper left pm^2, M^2, M^3 and RM_3 all had occlusal cavities. Wear, correlating with age, had exposed dentine on the incisive surfaces of upper central incisors, on the anterior cusps of the

maxillary 1st molars and on the buccal and palato-distal cusps of the mandibular 1st molars.

- XAN023 (Malefakis plot – Tomb 1 – Individual 3) is an adult female of ca. 25-35 years, burial 3, found below burial 2 (XAN022) at a depth of 3.30 / 3.70m, under a huge stone with some plain sherds. Represented by a few long bones and a damaged skull, she also lay supine with her head southwest towards the chamber. Mandibular and maxillary teeth with cavities and ante-mortem tooth loss (Supplementary Figure 17), indicate poor dental health.

- XAN024 (Malefakis plot – Tomb 1 – Individual 4) is the fourth burial in the dromos, a young adult male found at a depth of 4.00-4.10m in front of the entrance to the chamber. Positioned in an east-west orientation with head to the east, enclosed in an irregular pit cut into the rock, lined with many small stones. There was a large slab in front of the knees and the posture of the body seemed peculiar placed face down: the upper spine was twisted, and the arms appeared to be behind the back.

- XAN025 (Malefakis plot – Tomb 3 – Individual 2) is a young adult female in a secondary burial in a rectangular niche in the northwestern corner of the chamber. Her cranium exhibited remarkable metopism and multiple wormian ossicles on both branches of the lambdoid suture. Two loose maxillary teeth, identified as RI$^{\underline{1}}$ and a LC presented linear enamel hypoplasia. There were four fragments of a mandible and 9 mandibular teeth, five of them with cavities and most with substantial deposits of calculus. A left femur~~,~~ gave a stature estimate of 143.267 ± 3.72cm Supernumerary body parts, indicated the presence of a second, taller most probably a female individual. A stature of 156.603cm was estimated from a right radius.

- XAN026 (Tomb 3 – Individual 3) are the skeletal remains of a young adult female individual commingled with a second female, a male skeleton and animal remains found piled along the western side of the chamber. This anatomically female cranium presented external auditory exostoses (EAE), more pronounced on the left than the right. EAEs are associated with prolonged exposure of the auditory canal to cold water.

They have been reported extensively in Holocene skeletal samples where people engaged in exploiting aquatic resources, which seems plausible since the site is close to the sea. A stature of 150.677 ± 3.72 cm was estimated from a complete left femur.

- XAN027 (Malefakis plot – Tomb 3 – Individual 1) belongs to a poorly preserved adult male skeleton found in the dromos in front of the entrance to the tomb resting in a foetal position and surrounded by stones and covered with slabs. The aperture used to plunder the tomb was just to the south of the burial. This material included 5 femurs representing a minimum of 3 people, as well as 3 tibias (two left and one right).

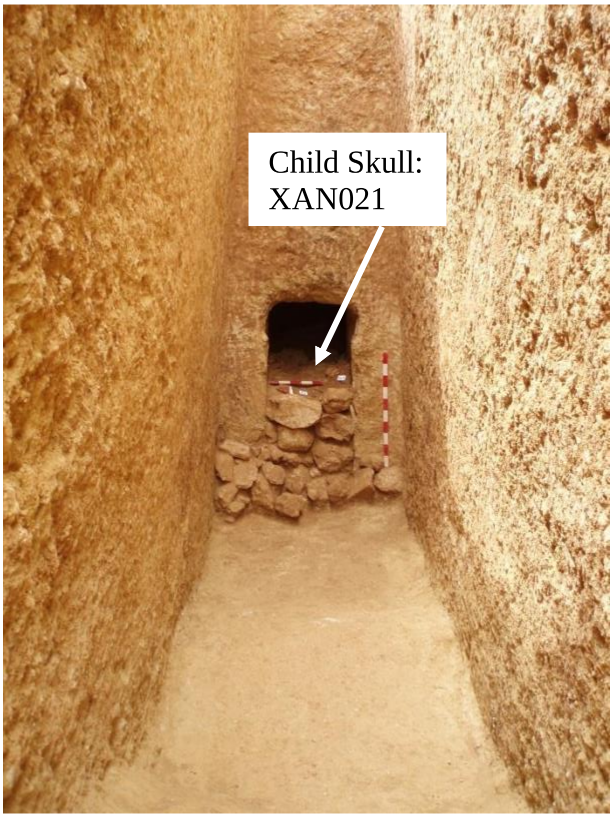

Supplementary Figure 14. Malefakis Plot, Tomb 1.

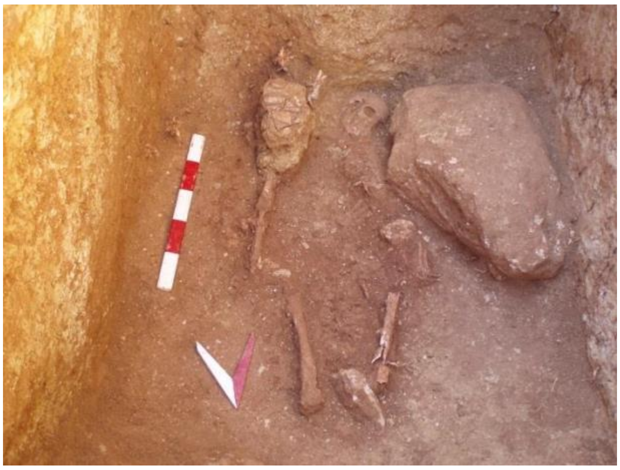

Supplementary Figure 15. Malefakis Plot, Tomb 1. Individual 2.

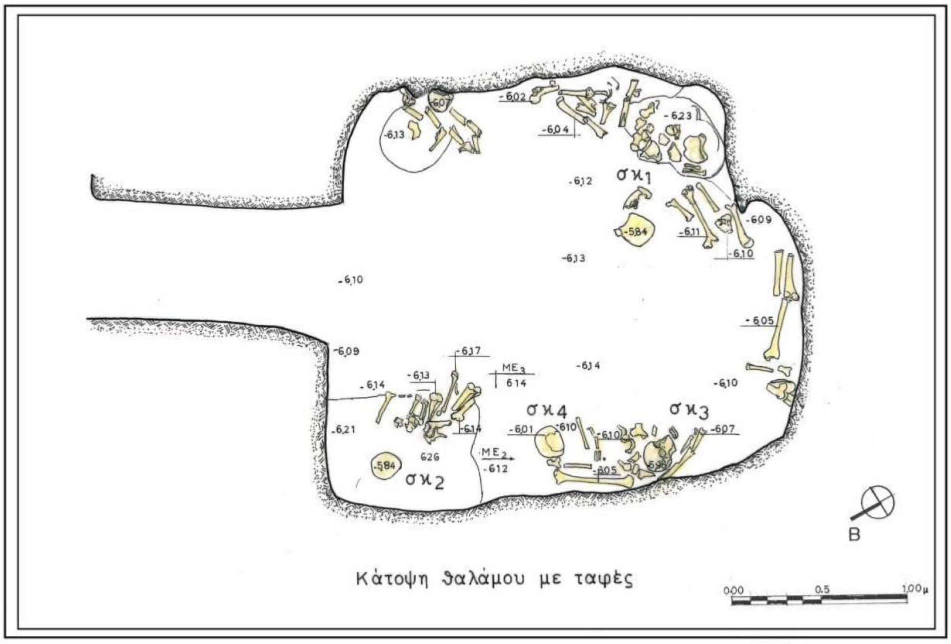

Supplementary Figure 16. Malefakis Plot; Tomb 3. Plan of the chamber (‘σκ’: skeleton).

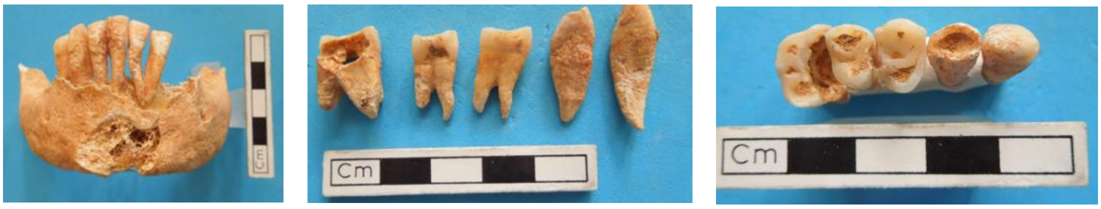

Supplementary Figure 17. Malefakis plot, XAN023: mandible, maxillary teeth M^1, $Pm^{1\text{-}2}$, C^1, I^2 with cavities and wear

7. Kouklakis Plot

Sixty tombs dating to LM II-LM IIIA2 (ca. 1450-1300 BC) were excavated in 2004 in the Kouklakis plot (Andreadaki-Vlazaki and Protopapadaki, 2009; Andreadaki-Vlazaki, 2010) (Supplementary Figure 18). Apart from chamber tombs, the majority were pit-caves and graves, dating to LM II and IIIA1 (about 1450-1370/1350 BC). Most pit-caves consist of a ca. 3m deep narrow trench with a cave-like opening, a chamber or niche, in one long side of the pit, intended for a single burial (Supplementary Figure 19). A rough-and-ready dry-stone blocking sealed the opening.

Among the tombs, two examples stand out: the LM II pit-cave no. 40 and the LM IIIA1 mainland-type shaft grave no. 46. Their weapons were symbols of warrior superiority and status. The admixture of funerary architectural types: of chamber tombs, pit caves, shaft and pit graves, suggests the advent of peoples from different regions. This extraordinary combination of tomb types and burial customs, practised by people springing perhaps from different tribal and cultural origins, apparently flourished in this new setting.

Samples for aDNA analysis were taken from 12 tombs, but there was no ancient DNA preservation from the warrior tombs. The only results came from individuals in tombs N° 25 and 36, which are dated to LM IIIA1 and A2 (ca. 1400-1300 BC).

Tomb 25 is a chamber tomb. The dromos is 8.66m long and 3.00m deep. The dimensions of the chamber are 2.40 x 2.10 x 2.15m. A deceased was placed in a rectangular pit in the floor of the dromos. In the chamber, two burials were found in situ **(XAN042)** accompanied by a single clay vase. On the east side of the chamber, the bones of two more individuals, a man and a woman **(XAN040, XAN041)**, were placed in heaps, also accompanied by only one clay vase.

Tomb 36 is a pit grave that received successive burials of children **(XAN051, XAN053)**, two males and a female. The three children were covered by a ritual deposit with an abundance of domestic pottery, prominent among them cooking utensils. The excavation as a whole is not yet published.

- XAN040 (Kouklakis plot - Tomb 25 – Skull 1) is a secondary burial with a well-preserved cranium and rugged skeleton of a young adult male, in the 17–25-year age range. The young man had an apical abscess above upper Lpm^1 and traces of calculus on right and left pm^2, M^1 and M^2 were noted. The medial right tibia presented periostitis. Mean stature was 166.62cm.

- XAN041 (Kouklakis plot - Tomb 25 – Skull 2), also a secondary burial (buried with XAN040), includes the partially preserved cranium and completely edentulous mandible of an elderly female, ca. 50-60 years old. The cranial sutures were entirely obliterated on the endocranium and, except for coronal 1-2 and lambdoid 2-3, were barely visible on the exocranium. Of the post-cranial remains, only the right clavicle and right femur were complete, the latter providing a stature estimate of 147.59cm.

- XAN042 – The material is being prepared for a detailed publication.
- XAN051 - The material is being prepared for a detailed publication.
- XAN053 - The material is being prepared for a detailed publication.

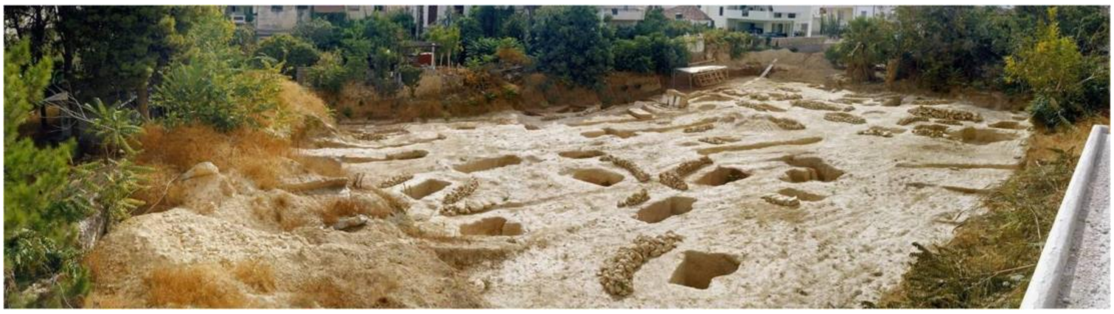

Supplementary Figure 18. Kouklakis Plot. General view of the excavation.

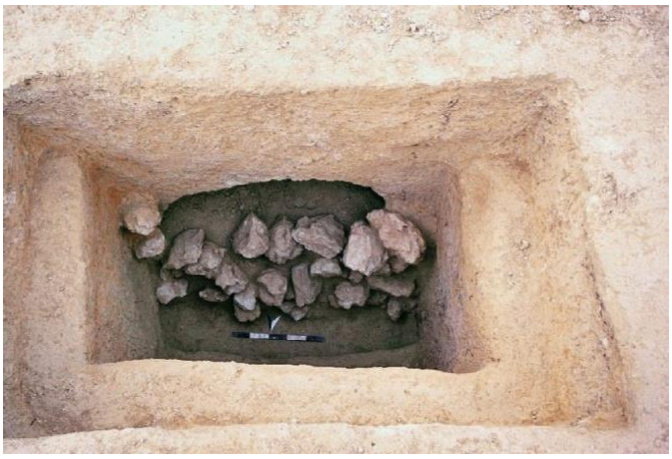

Supplementary Figure 19. Kouklakis Plot. A pit cave.

8. Tsapakis plot

Between 2010 and 2018, eight tombs were excavated in the Tsapakis plot, located to the west of the Kouklakis plot. Six date back to the early Hellenistic period. The other two tombs are chamber tombs that date to LM IIIB1 (ca. 1300-1250 BC: tomb 5) and LM IIIC1 (ca. 1200-1150 BC: tomb 1). Sample **XAN028** comes from tomb 5 (Supplementary Figure 20), with a spacious chamber of $11m^2$, approached by a 7.50m long and 3.70m deep dromos (Supplementary Figure 21). The chamber had collapsed to half its height. Although there were no signs of looting, the fill was very disturbed. Fragmented clay offerings were found on all sides of the tomb.

- XAN028 (Tsapakis plot – Tomb 5 - Individual) is a female skeleton, represented by a mandible. She was accompanied by grave gifts including a bronze knife, a carnelian sealstone and nine clay vases: a small piriform jar, a mug, the lid of an incense burner, a kylix, and two one-handled cups. The most interesting offerings in the ensemble are two hybrid types of ritual vessels made in the local pottery workshop of Kydonia. The first is an incense burner with four ventilation holes, the second a triple ritual vessel with two plastic female heads, whose elaborate ritual headdress, a rendering of divine figures characteristic in Aegean iconography, affirms the religious character of the vase.

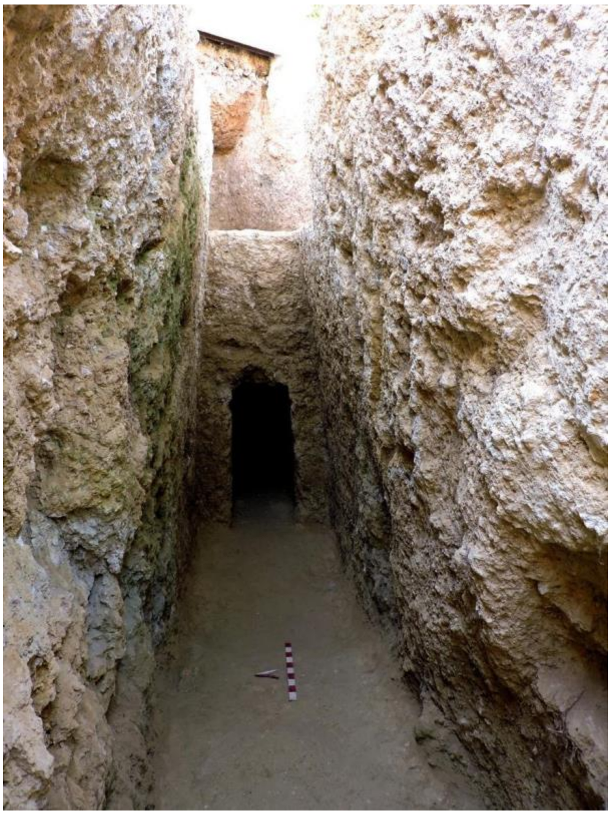

Supplementary Figure 20. Tsapakis Plot. Tomb 5. Dromos.

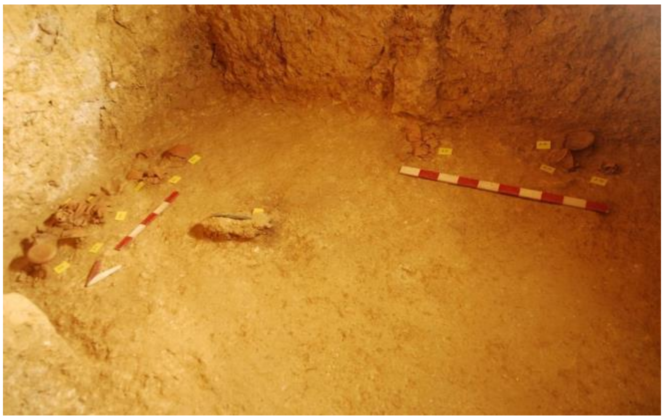

Supplementary Figure 21. Tsapakis Plot. Tomb 5. Chamber.

9. Rovithakis plot

A cluster of five chamber tombs was excavated in 1996 in the Rovithakis plot in the western part of the cemetery (Andreadaki-Vlazaki, 2003; Preve, 2009b). Tombs 1 and 2 are impressive examples of underground rock-cut subterranean burial monuments of prehistoric Kydonia. Tomb 1 has a dromos 14.40m long and 5.80m deep and a chamber measuring 2.95 x 3.55 x 2.10m. It was plundered in antiquity. Three stirrup jars were found by the entrance to the chamber, and nearby was the burial of a dog. Part of the walls and ceiling of the chamber had collapsed. Two disturbed burials were lying on the floor **(**one of which belonged to the male **XAN029)**, and bones of a third skeleton had been placed in a heap. Fragments of decorated plaster testify to the presence of wooden coffins for the dead. Objects that escaped the looters are a stirrup jar, a one-handled cup, a censer, a glass bead and a rock crystal pendant that date to the LM IIIB1 period (ca. 1300-1250 BC).

Only the eastern part of the dromos of the tomb 4 (maximum dimensions: length 3.60m, width 1.85m, depth 6.85m) could be explored. Some quite bulky, worked stones probably belong to a grave marker. Male skulls 3 and 4 (**XAN030** and **XAN031**, respectively) were uncovered in the thick layer of fallen stones. Two more skulls were collected from the fill in front of the chamber, along with a small bowl. Two burials had been placed on the blocking wall of the entrance to the chamber in the Subminoan period (ca. 1100-1000 BC). They were accompanied by two pieces of sealstones, a bronze fibula, two bronze rings and a glass bead and had been partly damaged during the looting of the tomb. The plundered chamber was

square, measuring approximately 3.00 x 3.05 x 2.75m. Five skulls (one of which belongs to the female **XAN034**; N° 8) and scattered bones were left on the floor. Among the grave offerings are vases (a spouted cup, a kalathos, another double one, a stirrup jar and pieces of a kylix and an amphoroid krater), stone spindle whorls and a bronze utensil that date the burials to the LM IIIB1 period (ca. 1300-1250 BC). A few bones of a dog and a sheep were also collected from the chamber. The material is being prepared for a detailed publication.

Hagios Charalambos, Lasithi, Greece

Coordinates: 35.1772505, longitude 25.4410963

Excavation(s): Ephorate of Antiquities 1976 and 1982-1983, directed by Prof. Costis Davaras; The American School of Classical Studies at Athens, 2002-2003, directed by Prof. Philip Betancourt and Prof. Costis Davaras. Bioanthropological research: Dr. P.J.P. McGeorge

The Hagios Charalambos Cave (also known as Gerontomouri), on the Lasithi Plain in the mountains of central Crete, was discovered by chance in 1976. During the construction of a road that passed in front of the cave, dynamiting operations destroyed the roof of two outer chambers of the original seven, brimming with human remains. A spectacular volume of human and animal skeletal remains was recovered during the excavations of 1976, 1982-83 (Davaras, 1983; Davaras, 1986; Davaras, 1989b; Davaras, 1989a; Davaras, 2015; Davaras, 1976) and 2002-2003 (Betancourt, 2014; Betancourt et al., 2008). The bones are amazingly well-preserved because of the low temperatures in the cave. With only minor temperature fluctuations, it was a natural refrigerator and contributed to unprecedented standards of preservation for human remains of this early date, making them ideal specimens for aDNA analysis (McGeorge, 1988).

The Lasithi Plain has a microclimate and in harsh winters is often cut off by snow for months. This must have been the case in the past so that the people of this relatively remote region were isolated for periods, though not totally, since the study of the pottery from the cave indicated that in successive periods, people came under alternate, competing spheres of external influence. This is relevant for an understanding of the genomic data, which suggests a significant degree of endogamy.

In the Neolithic and Early Minoan periods, caves were often used for primary burial, but the Hagios Charalambos cave was used as an ossuary in the Middle Minoan IIB for the secondary burial of human remains originally interred elsewhere. This is evident from the many

bones that are "etched" with root impressions, although the cave itself had virtually no soil other than sediment washed into it. The pottery provides the best dates for the material placed in the ossuary (Langford-Verstegen, 2015). Although it was all placed in the cave in a single episode, the pottery shows that it consisted of burials that had been made over a long period of time, from Late Neolithic II (4th millennium BC) to Middle Minoan IIB (18th century BC). Neolithic pottery was scattered throughout from the modern surface to the floor of the cave. These remains can only have come from a long series of burials that were removed from their original place of deposition and placed in the cave without concern for their original contexts. Along with the bones, those who moved the remains brought objects that had been placed in the original graves, including jewellery, tools, pieces of pottery, seals, and other items. Joins of pottery fragments found in different rooms help attest to the random nature of the collection and deposition. Stones intrusive to the context of the cave suggested a possible origin from built tombs, but no trace of tombs has yet been found. An alternative, elegantly-reasoned hypothesis (Davaras, 2015) proposes that the Psychro Cave, only 1km away, could have been the original location of the primary burials, which had to be removed to purify it when Psychro became the focus of cult worship in the Middle Minoan period.

The remains under study, derived from all campaigns, constitute perhaps the largest and best-preserved corpus of human material from such an early period. These commingled burials, mixed with grave goods that range in date from the Neolithic to Middle Minoan, with some Late Minoan offerings, remained undisturbed following the Bronze Age. The placing of all the human remains together makes us reflect on a different sense of individuality in that period. Mindful, however, of rapid progress in archaeological techniques, a large volume of material was left behind for future researchers, and the cave was sealed in 2003 to protect it from potential looters.

The transfer of the primary burials to their new location was clearly a major undertaking, but the care with which the bones were collected is revealed by the fact that virtually every part of the anatomy is represented in the burial corpus, although in varying frequencies. As there are no discrete burials, each bone is treated as a separate individual, and so far, 32,000 records have been made. The chances of identifying bones that belong to the same individual are slim indeed. However, there are some exceptions. For example, the limb bones of a pituitary dwarf, fused vertebrae, and some articulated vertebrae suggest incomplete decomposition at the time of the transfer from the primary burial site.

As illustrated in Supplementary Figueres 22 and 23, there is little actual soil deposit in the cave. Post-excavation, it was determined that the secondary deposition was a single short

event, apparently without meaningful stratigraphy. Nonetheless, when the human remains were sorted into anatomical elements, the provenance of each bone continued to be recorded in case this information should turn out to be useful. Printed provenance labels have been attached to every bone or fragment. Where broken fragments of a single bone united, there can be several provenance labels for the same bone.

The size of the sample is still not yet reliably calculable, and there are many, many fragments of skulls still to be restored and examined. Almost 5000 tarsals, metatarsals and pedal phalanges have been studied. Of the latter, the 1109 calcaneus and talus bones, which are fairly dense and durable bones, are the most numerous. The right calcaneus has given a minimum number of individuals (MNI) of 292. Sorting the bones into anatomical elements allows one to group and seriate them, enabling one to study and observe variations in comparative morphology and size. This facilitates differentiation of sex and age in the population and permits one to assess the burden of pathology affecting particular areas of the anatomy and the differences in the distribution of pathology between the sexes. Surprisingly, no more than 10-12% of the material appears to be immature. The percentage of immature individuals may be low due to the greater fragility of subadult remains, although perhaps that is not the entire explanation. In addition, the immature individuals are affected by pathology which reveals their participation in arduous chores causing repetitive injury to the articulations of very young limbs (Supplementary Figure 24).

There is evidence that life was not always peaceful and that it could be "nasty, brutish and short", but there were skilled medical practitioners who could prolong life. The study has provided amazing examples of audacious surgical interventions (McGeorge, 2006) which patients survived. Trephination seems to follow Hippocratic procedure long before the Hippocratic treatises were written. There is also evidence for a range of pathologies: traumas, arthropathies, spondyloarthropathies, neoplasm (McGeorge, 2019), endocrine and genetic disorders.

All of the teeth that have been analysed for DNA come from Room 5, which was excavated in 2002 and 2003. The room was a roughly elliptical space ca. 4 x 6m in size at the base, oriented with the long dimension east-west. It could be entered in antiquity either from Room 4 in the south or from Room 3 in the east, but at the time of excavation, the deposit of bones inside the room was so deep that less than a meter of space existed at the entrances. In the west, a hole led down to Room 7. The eastern end of the room was filled with stones that had fallen from the highly cracked ceiling, and the fallen blocks supported the ceiling at the northeast of the room.

The teeth for DNA analysis were collected by a staff member (Louise Langford-Verstegen) at one time in the centre of Room 5 several centimetres below the surface of the deposit. The deposit of Room 5 consisted of mixed human bones, animal bones, stones, soil, pottery, and a number of other artifacts. It had no internal stratigraphy except for the human skulls placed on top of the deposit. The room had been prepared for the deposition of the human remains by the construction of two rubble walls oriented north-south, one at the west beside the hole leading down to Room 7 and one near the centre of the room. These walls helped retain the deposit because the floor sloped down toward the west. At its deepest, the top was ca. 1.6m above the floor. Like most other items in this deposit, they were not associated with any specific artifacts or other human bones. In the aDNA lab (MPI-SHH), a minimum number of unique individuals was defined, and 42 well-preserved teeth were selected for aDNA analysis. After merging data from samples determined after analysis to be from the same individuals and excluding those of poor quality (Methods), genome-wide data from a total of 28 individuals were assembled (21 males/7 females). Radiocarbon-dating analyses on eight samples suggest that they fall mainly within the Early Minoan II and III periods (second half of the third millennium BC). This date is compatible with some of the pottery found in Room 5 (Langford-Verstegen, 2015). The teeth would have been old when they were deposited in the cave, which may help explain why they were loose within the deposit.

- Radiocarbon-dating on human tooth HGC001.A: 3732±19 BP, 2200-2041 cal BC (95% probability), (ID: MAMS-37426, AMS, IntCal13).
- Radiocarbon-dating on human tooth HGC002.B: 3777±19 BP, 2283-2139 cal BC (95% probability), (ID: MAMS-37427, AMS, IntCal13).
- Radiocarbon-dating on human tooth HGC003.A: 3648±19 BP, 2125-1948 cal BC (95% probability), (ID: MAMS-37428, AMS, IntCal13).
- Radiocarbon-dating on human tooth HGC004.A *(no genome-wide data reported)*: 3716±22 BP, 2196-2034 cal BC (95% probability), (ID: MAMS-37429, AMS, IntCal13).
- Radiocarbon-dating on human tooth HGC005.A: 3757±19 BP, 2276-2059 cal BC (95% probability), (ID: MAMS-37430, AMS, IntCal13).
- Radiocarbon-dating on human tooth HGC006.A: 3764±19 BP, 2279-2063 cal BC (95% probability), (ID: MAMS-37431, AMS, IntCal13).

- Radiocarbon-dating on human tooth HGC007.A *(no genome-wide data reported)*: 3725±19 BP, 2198-2038 cal BC (95% probability), (ID: MAMS-37432, AMS, IntCal13).
- Radiocarbon-dating on human tooth HGC008.A: 3795±19 BP, 2290-2146 cal BC (95% probability), (ID: MAMS-37433, AMS, IntCal13).
- Radiocarbon-dating on human tooth HGC026.A: 3751±25 BP, 2280-2039 cal BC (95% probability), (ID: MAMS-49762, AMS IntCal20).

This assemblage of human teeth provides an examination of Minoan DNA from an early phase of Minoan culture. It comes from an interior part of the island of Crete, from a period well before the increased trade and travel that characterizes the island in the Late Bronze Age (after 1500 BC). The radiocarbon analysis indicates a date before the major palaces were built at Knossos and elsewhere early in the Middle Bronze Age.

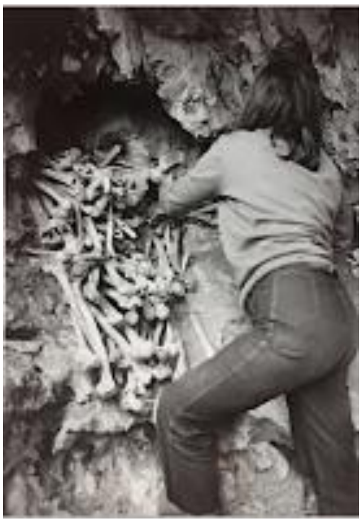

Supplementary Figure 22. Hagios Charalambos outer chamber, 1983.

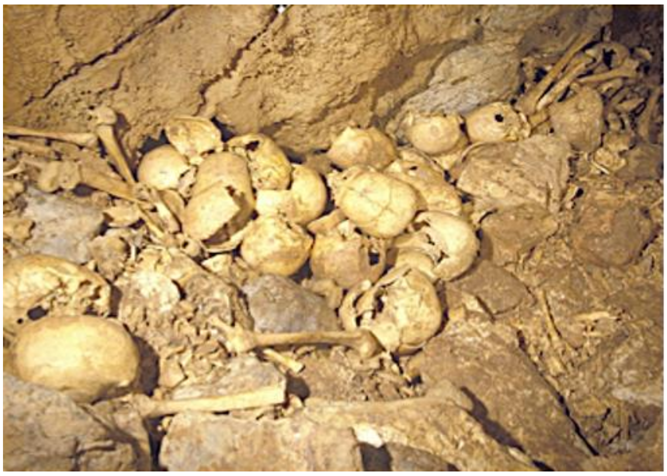

Supplementary Figure 23. Hagios Charalambos chamber 5, 2002.

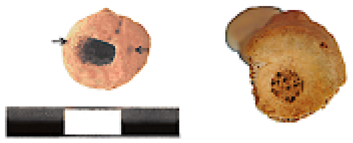

Supplementary Figure 24. Radii of a child and an adult with epiphyseal lesions.

Krousonas, Heraklion, Greece

Coordinates: 35.2394569205021, 24.9883048004559

Excavation: Antiquities for the Heraklion Prefecture, 2011, directed by Dr. Athanasia Kanta (Director Emerita) and for bioanthropological research by Prof. Anagnostis P. Agelarakis.

The built, square tholos tomb of Krousonas (Supplementary Figures 25 and 26) came to light during work operations for a road leading to the village. In the interior of the tomb were a sarcophagus, vases, other burial gifts and skeletons. A preliminary report of the excavation is available (Kanta and Serpetsidaki, 2013). The tomb contained at least ten burials. It produced pottery and artefacts dating to the 12th and the first half of the 11th century BC (Supplementary Figure 27). Such tombs in Crete are usually family tombs and elite burial places.

Following the same method for sample collection described for the human skeletal remains from Aposelemis, samples from twelve individuals from the tholos tomb at Krousonas were sampled for DNA. We present genome-wide data and direct radiocarbon dates for two of them:

- KRO008 (Square A1: Stratigraphic Layer 1; Lab 2016-4) is an individual of estimated age range 35-55 years, tentatively assigned to female. No apparent skeletal pathologies were identified. Radiocarbon-dating on human bone (KRO008.A): 2975±24 BP, 1365-1114 cal BC (95% probability), (ID: MAMS-49524, AMS, IntCal20).
- KRO009 (Square A1: Stratigraphic Layer 1; Lab 2016-6-a) is an individual of estimated age range 35-50 years, tentatively assigned by limited morphoanatomic loci to female. Palaeopathological changes caused by spondyloarthropathies were identified (Supplementary Figure 28). Radiocarbon-dating on human bone (KRO009.A):

2970±22 BP, 1268-1060 cal BC (95% probability), (ID: MAMS-49525, AMS, IntCal20).

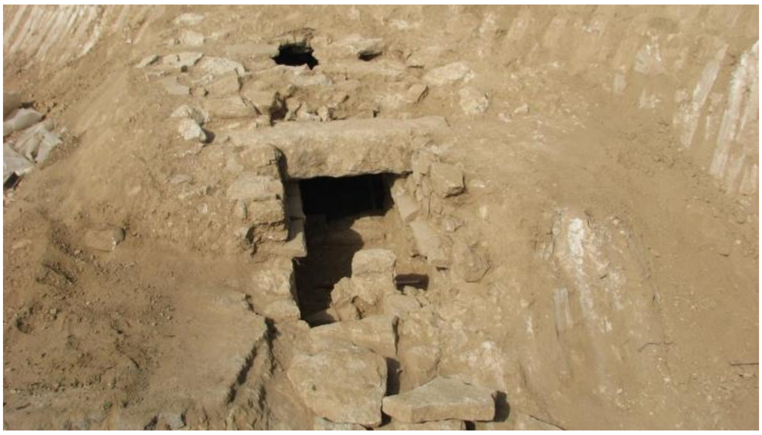

Supplementary Figure 25. The exterior of the Krousonas tholos tomb.

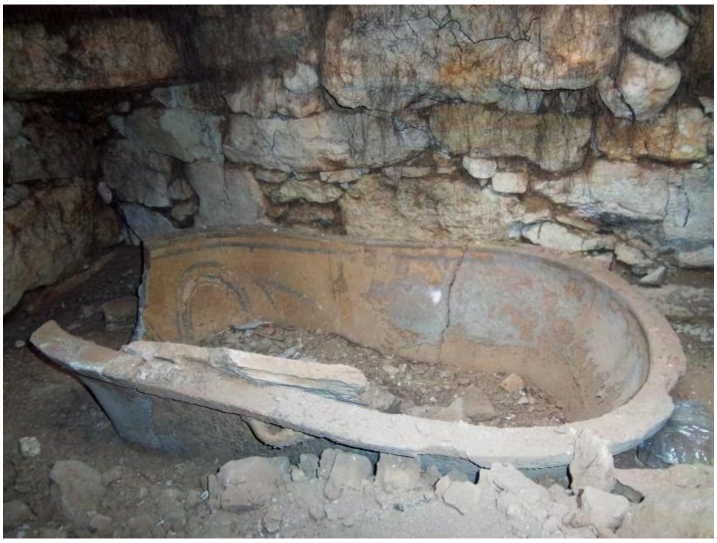

Supplementary Figure 26. The interior of the Krousonas tholos tomb.

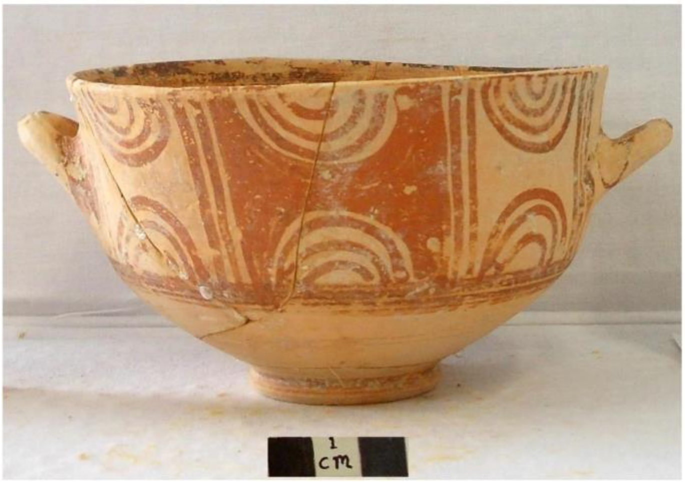

Supplementary Figure 27. Typical Late Minoan IIIC deep bowl.

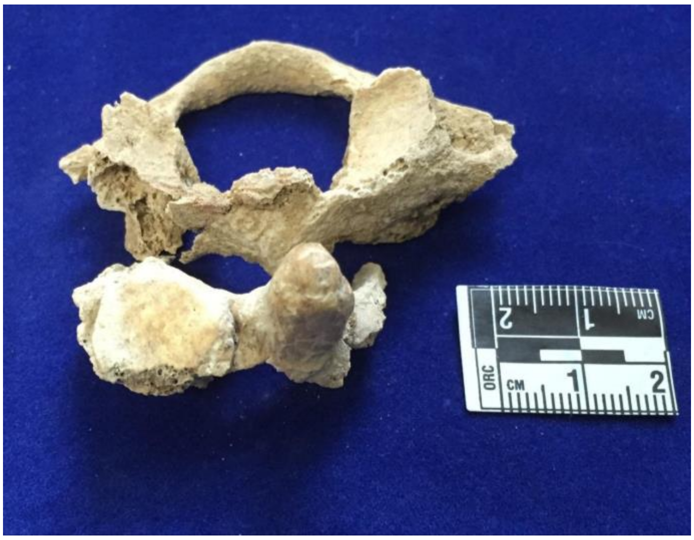

Supplementary Figure 28. A superior view of the first two cervical vertebrae, the atlas (top) and axis (bottom) preserved in an incomplete and fragmentary state due to taphonomic effects, recovered anatomically disassociated, yet morpho-anatomically belonging to the parent skeleton of an older female individual, revealing palaeopathological changes caused by spondyloarthropathies. On the atlas, they were recorded as manifestations of marginal lipping on both the fovea articularis superior and inferior bilaterally, as well as at the fovea dentis, while marginal osteophytic growths were documented peripherally to the fovea dentis, on the region of arcus anterior and inferiorly towards the basal adjacency of *tuberculum anterius*. On the axis, incipient marginal lipping manifestations were observed on the *facies articularis anterior dentis*, yet more derived marginal lipping was observed on the preserved, right side, *facies articularis superior* and *processus articularis inferior*. Due to taphonomic disturbances, the vertebrae can only be tentatively assigned to individual KRO009.

MAINLAND GREECE

Aidonia, Corinthia, Greece

Coordinates: 37.84083, 22.5832

Excavation(s): 4th Ephorate of Antiquities (Nafplion), 1978-1980 and 1986, directed by Kalliope Krystalli-Votsi and Konstantina Kaza-Papageorgiou; 37th Ephorate of Antiquities (Corinth), 2002, directed by Panagiota Kasimi; TAPHOS (Corinthian Ephorate of Antiquities and the Nemea Center for Classical Archaeology, UC Berkeley), 2014-present, directed by Konstantinos Kissas and Kim Shelton. Bioanthropological study: Dr. Gypsy Price.

The site of Aidonia is situated on the slopes of the northeastern Phlious Valley in southwestern Corinthia. Today located in olive groves, during the Late Bronze Age, the hillside was used as an extramural cemetery with at least three *systades* of chamber tombs (upper, middle, and lower) and associated mortuary features. The site was discovered through looting in the late 1970s, followed by rescue and systematic excavations (Krystalli-Votsi, 1998; Krystalli-Votsi and Kaza-Papageorgiou, 2013). The settlement was located by the Phlious Valley Survey in the early 2000s (Casselman et al., 2004; Hachtmann, 2013) and remains unexcavated.

Most of the tombs excavated in the late 1970s and early 1980s had been disturbed and looted, and precious material from them came onto the antiquities market during the early 1990s. These multi-burial and multi-generational tombs contained material dating to the Late Helladic (LH) period, from the 15th to early 13th centuries BC (LH II-IIIB1) and an unknown number of burials. The Middle Cemetery, centrally located on the hillside, is the location of the original cemetery known since the 1970s, and the focus of the TAPHOS excavations in 2015-2019, along with three chamber tombs in the Lower Cemetery in 2016-2018. Our project has produced evidence of burials from the late 16th to early 12th centuries BC (LH I-IIIC Early) based on a broad range of grave gifts and burial materials. The TAPHOS project has also found evidence of use in the area during the Geometric, Archaic-Classical, Late Roman, and Medieval periods.

To date, the TAPHOS project has excavated eight chamber tombs in the Middle and Lower Cemeteries with a MNI of 97 from primary and secondary burials. Primary burials were placed on the chamber floors and in cists under the floors, while a variety of burial rituals are exhibited through disarticulated secondary burials individually placed in cists and on the

chamber floor or commingled in piles or cists, some quite organized and others very mixed (Kvapil and Shelton, 2019).

Samples for this study were taken from 17 individuals from three chamber tombs excavated by TAPHOS in 2015-2016. Tombs 100 and 101 were adjacent to each other in the southeastern part of the Middle Cemetery. Both contained multiple individuals, with 100 in use ca. 1500-1375 BC (LH IIA-IIIA1) and 101 in use ca. 1375-1200 BC (LH IIIA-IIIB). Tomb 103 was one of two early and large chamber tombs located in the Lower Cemetery. It contained at least 12 individuals, several of which were primary burials in a central shaft-like cist, while others were secondary burials placed in a variety of deposit types. Sampling preference was largely based on preservation (individuals with preserved petrous portions, associated teeth - preferably with dental calculus). In addition, we focused on individuals from discrete contexts, but both primary and secondary burials were chosen.

Nine individuals from Aidonia produced genome-wide data that are presented in the genetic analyses:

- AID001 ([1] 15/100064) is an adult female in a primary burial context in the centre cist under the chamber floor of Tomb 100, which dates to LH IIIA1. The skeleton exhibits significant attrition but no apparent pathologies.
- AID002 (15/100028 skull A) is an adult (middle-aged) male in a secondary burial deposit from the Western Burial Platform of Tomb 100. This skull was one of the four that were arranged at the bottom of the deposit on the burial platform. It has no definitively associated skeletal elements; however, the upper left third molar was inverted and partially erupted well above the typical location, with no apparent associated abscess or lesion. The burial likely dates to LH II.
- AID007 ([3] 16/101132) is a young adult male, one of two primary burials at the bottom of Tomb 101's SW Cist. The burial dates broadly to LH IIIA-IIIB. It had no pathologies aside from a couple carious lesions.
- AID008 ([4] 16/101132), the second of two primary burials (with AID007 [3] above) at the bottom of Tomb 101's SW Cist, is also a young adult male with no apparent pathologies. The burial also dates broadly to LH IIIA-IIIB.
- AID009 ([6] 16/101133) is a young adult male from a secondary burial which overlay the primary burial ([7] 16/101133) in the West Cist of Tomb 101. This burial had no apparent pathologies and dates broadly to LH IIIA-IIIB.

- AID010 (16/101123 skull K) is an adult female. This individual is one of at least three fragmentary secondary burials scattered throughout the eastern half of Tomb 101 near the original floor surface. The deposit dates to LH IIIB.
- AID012 ([3] 16/103058) is a middle-aged female in a primary burial context within the Centre Cist of Tomb 103 dating to Late Helladic IIA. This is one of several primary burials placed on top of and alongside each other on the floor of the deep shaft-like cist in the centre of the large chamber. Burial [3] immediately preceded in death/burial the final primary burial in the cist ([2]). It exhibited no apparent pathologies or trauma.
- AID014 (16/103056 skull A) is a young adult female. This individual is one of at least three likely female individuals from a secondary commingled deposit in a shallow oval cist located in the northeastern portion of Tomb 103's Dromos. There are no apparent pathologies. The deposit has a *terminus ante quem* of LH IIB but contains earlier material as well, including sherds that join vases from the Centre Cist (see contexts of AID012 and AID017).
- AID017 (16/103058 skull) is an adult female from a secondary burial of fragmentary and commingled remains of two individuals at the northern end of Tomb 103's Centre Cist. Preservation and the dissociation of elements prevents any further biological profiles, while associated ceramic material suggests a date of Late Helladic I-IIA.

Glyka Nera, Attica, Greece

Coordinates: 37.9901700, 23.8499070

Excavation(s): B΄Ephorate of Prehistoric and Classical Antiquities, 1991-2002, directed by E. Kakavogiannis and O. Kakavogianni (Honorary Director).

Bioanthropological study: Dr. Anastasia Papathanasiou.

The cemetery of Glyka Nera is a rich and extensive Mycenaean, Late Helladic chamber tomb cemetery, radiocarbon-dated around 1400-1325 BC. The site is situated in eastern Attica on a plain not far from the Aegean coast. Salvage excavations started in 1991 (Kakavoyannis, 1999-2001), and no settlement or palatial centre has yet been located. The cemetery consists of 22 unlooted chamber tombs and a number of pit burials. Notable is a considerably larger square chamber tomb with exceptional, valuable, symbolic, imported offerings, some of clear Minoan

origin. All tombs were reused for multiple inhumations, representing 102 partial individuals, including a variety of pottery, seals, beads, and a number of terracotta or lead figurines (Polychronakou-Sgouritsa and Kakavogiannis, 2020).

The study of the human assemblage from Glyka Nera confirmed trends of social and gender differentiations (Papathanasiou et al., 2020), compared to other Mycenaean sites or within the same site, between the Large Chamber and the rest of the tombs. Subadult underrepresentation, pointing towards differential burial treatment of these age groups and preferential access to the chamber tomb cemeteries, is observed in Glyka Nera, as in all Mycenaean chamber tomb and tholos cemeteries, but Glyka Nera is differentiated from other Mycenaean cemeteries in resembling more peripheral regions of the Peloponnese and less those of the palatial centres of southern Greece, as there is a presence, though modest, of subadults and especially of individuals under the age of four. Statistical analysis of the prevalence of dental pathological conditions showed that ante-mortem tooth loss (AMTL), linear enamel hypoplasia (LEH), and caries are significantly higher in females, implying that females in Glyka Nera, both in childhood and in adult life, may have experienced a lower health status, parallel to similar observations in the chamber and tholos tomb burials in the Athenian Agora and Pylos.

Bone collagen samples from 40 individuals were analysed for stable carbon and nitrogen isotopes, showing a primarily C_3 terrestrial, plant-based diet. Compared to other Bronze Age sites, Glyka Nera individuals have high $\delta^{15}N$ values, similar to individuals from the royal tholoi of the palatial centers of Pylos, Kazanaki, and Grave Circles A and B in Mycenae, and comparatively higher than the observed values in individuals buried in chamber tombs at those and other Late Helladic sites. Differential access to animal protein resources in the Mycenaean world has been documented, with individuals buried in richer tombs showing better, protein-rich diets, reflecting consistent status differences. The diet at Glyka Nera, as a whole group, conforms with one similar to the richer and higher status Mycenaean tombs.

An intra-site statistically significant difference was observed in the mean $\delta^{15}N$ values between individuals who were buried in the Large Chamber tomb and those who were interred in the other graves, implying that individuals in the Large Chamber tomb at Glyka Nera were consuming more animal protein in the form of meat and/or dairy products than those in the other tombs. Since the Large Chamber tomb is also differentiated in its material culture, being richer in offerings of special importance and Minoan origin, and is more prominent architecturally, the isotopic data corroborate possible status differences within this community.

In the case of Glyka Nera, questions about the origin of the prominent individuals of the Large Chamber are raised by the presence of exquisite ritual artefacts which were most probably of Minoan provenience. Finally, the possibility of genetic affinities within the entire group should be investigated, as the prevalence of certain cranial non-metric traits is relatively high.

Three individuals from Glyka Nera were sampled for DNA. We present genome-wide data and direct radiocarbon dates for two of them:

- GLI002 (K2) is a male individual of 30-40 years of age at death represented by a partial cranium and mandible. He was one of the individuals buried in the Large Chamber in the Vorylla plot. A number of pathological conditions were observed on these cranial remains, including porotic hyperostosis on the left parietal, LEH on the lower canines, and an abscess on a right first molar. The burial is dated to the LH IIA-B (ca. 1400-1325 BC). Radiocarbon-dating on human bone (GLI002.A): 3103±22 BP, 1429-1293 cal BC (95% probability), (ID: MAMS-49522, AMS, IntCal20).
- GLI003 (Individual 3) is a male individual of 40-50 years of age at death represented by an almost complete cranium, the right innominate, and partial long bones. He was found in Chamber 5 of the Krystalli plot, a small chamber tomb containing the highest number of individuals and common offerings. Ante-mortem tooth loss of all right mandibular teeth was observed. The burial is dated to the Late Helladic IIA-B (ca. 1400-1325 BC). Radiocarbon-dating on human bone (GLI003.A): 3110±27 BP, 1421-1313 cal BC (95% probability), (ID: MAMS-49523, AMS, IntCal20).

Mygdalia, Achaea, Greece

Coordinates: 38.186889, 21.776528

Excavation: Ephorate of Antiquities of Achaea, 2008-present, directed by Dr. Lena Papazoglou-Manioudaki, co-directed by Dr. Constantinos Paschalidis.

Bioanthropological study: Dr. Olivia A. Jones.

The excavation on Mygdalia hill (Papazoglou-Manioudaki and Paschalidis, 2017) provides a unique opportunity to investigate the life ways of a local Mycenaean society in the Patras region of Achaea, the settlement, the cemeteries, and the resources available (arable land, areas for herding, water supply).

Mygdalia belongs to the group of Mycenaean settlements that were founded in the transitional period of Middle Helladic III/Late Helladic I (Mygdalia I) and rose to local prominence in the Early Mycenaean period (Mygdalia II). Substantial architectural remains, floor deposits and a tholos tomb furnished with pottery, that now finds parallels in settlement strata, will help define this important period in western Achaea. Its floruit came to an abrupt end at the beginning of the Palatial period, and continuation of full-scale habitation on the hill remains ambiguous (Mygdalia III) until its new floruit in the 12th century BC (Mygdalia IV). The mansion on top of Mygdalia hill (Terrace 1) and a large storeroom (Terrace 2) provide evidence for social organization in the Postpalatial period, the time of chamber tomb cemetery reuse and the warrior graves in Achaea.

The primary domestic space at Mygdalia was found on Terrace 2 (Supplementary Figure 29). The area consists of densely built houses with rectangular rooms, semi open spaces and courtyards. Also found on Terrace 2 were four Mycenaean intramural children's graves (Supplementary Figure 29), containing the remains of multiple infant and child inhumations interred in stone cists without grave goods, and two Archaic adult burials containing single primary inhumations (Papazoglou-Manioudaki et al., 2019) dating from the onset of the 7th century BC (Mygdalia V) when the area was transformed into an early Greek temple.

Intramuros Child Grave 3 is a stone slab cist grave with cover stone that is located just outside of the settlement's wall (Papazoglou-Manioudaki et al., 2019) (Supplementary Figure 30). A minimum of eight individuals could be identified, and the completeness of the skeletons suggest primary burials with secondary manipulation likely occurring when subsequent individuals were added to the grave. No grave goods were included. Despite the good preservation of the skeletons, the body positions, orientations and the temporal sequence of the interments could not be easily reconstructed due to the disarticulation and commingling of the bones. In total, eight petrous portions from the left temporal bones were sampled for DNA. We present genome-wide data and direct radiocarbon dates for seven of them:

- MYG001 (Intramuros Child Grave3.A) is a perinatal infant of 30-40 weeks in utero with no apparent pathologies. Radiocarbon-dating on human bone (MYG001.A): 3265±21 BP, 1611-1457 cal BC (95% probability), (ID: MAMS-47527, AMS, IntCal20).
- MYG002 (Intramuros Child Grave3.B) is a perinatal infant of 30-40 weeks in utero with no apparent pathologies. Radiocarbon-dating on human bone (MYG002.A):

3318±21 BP, 1626-1518 cal BC (95% probability), (ID: MAMS-47528, AMS, IntCal20).

- MYG003 (Intramuros Child Grave3.C) is an infant of 30-40 weeks in utero with no apparent pathologies. Radiocarbon-dating on human bone (MYG003.A): 3318±21 BP, 1596-1438 cal BC (95% probability), (ID: MAMS-47529, AMS, IntCal20).
- MYG004 (Intramuros Child Grave3.D) is a perinatal infant of 30-40 weeks in utero with no apparent pathologies. Radiocarbon-dating on human bone (MYG004.A): 3318±21 BP, 1609-1446 cal BC (95% probability), (ID: MAMS-47530, AMS, IntCal20).
- MYG005 (Intramuros Child Grave3.E) is a perinatal infant of 30-40 weeks in utero with no apparent pathologies. Radiocarbon-dating on human bone (MYG005.A): 3198±23 BP, 1504-1425 cal BC (95% probability), (ID: MAMS-47531, AMS, IntCal20).
- MYG006 (Intramuros Child Grave3.F) is a perinatal infant of 30-40 weeks in utero with no apparent pathologies. Radiocarbon-dating on human bone (MYG006.A): 3262±29 BP, 1612-1452 cal BC (95% probability), (ID: MAMS-47532, AMS, IntCal20).
- MYG008 (Intramuros Child Grave3.H) is an infant one-three months old with no apparent pathologies. Radiocarbon-dating on human bone (MYG008.A): 3262±29 BP, 1611-1452 cal B-(95% probability), (ID: MAMS-47533, AMS, IntCal20).

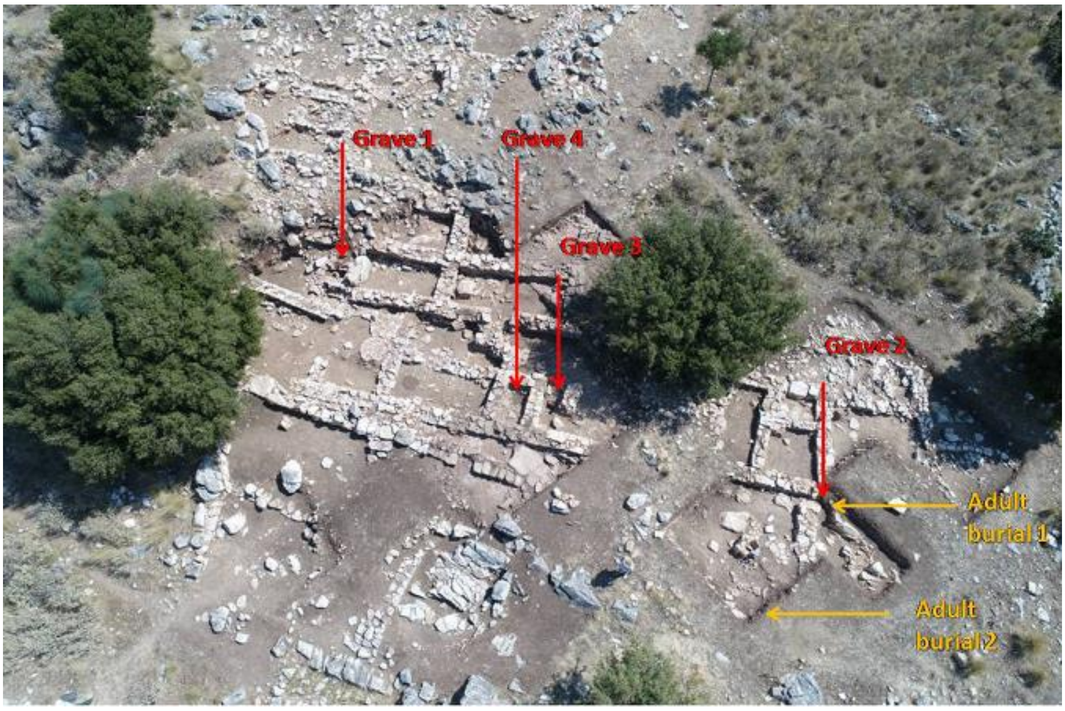

Supplementary Figure 29. Plan view of the Terrace 2 in Mygdalia. The locations of the four infant graves are marked.

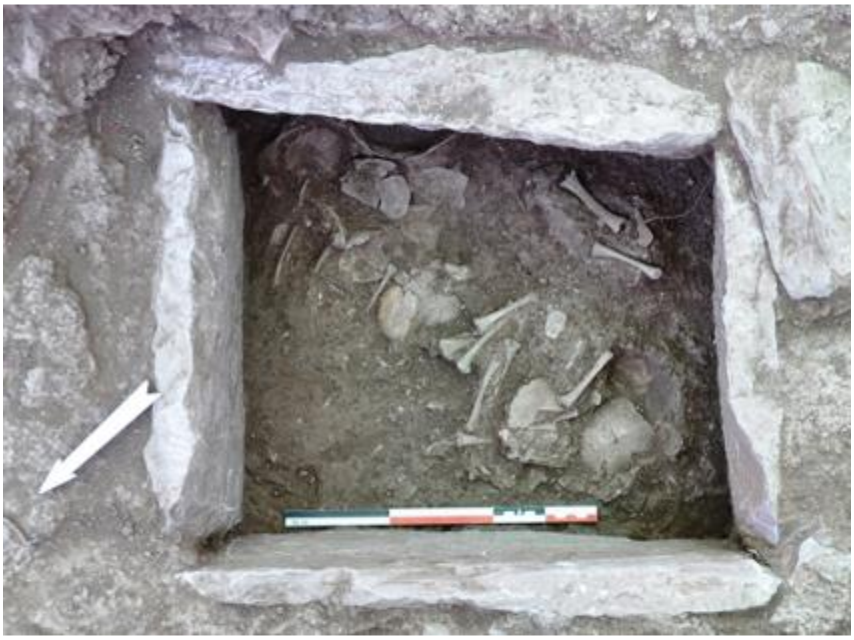

Supplementary Figure 30. Intramuros Child Grave 3 at Mygdalia.

Nea Styra, Euboea, Greece

Coordinates: 38.17975, 24.207417

Excavation: 11th Ephorate of Prehistoric and Classical Antiquities (currently Ephorate of Antiquities of Euboea), 2009, directed by Maria Kosma (rescue excavation). The excavation of Graves 2 and 3 was completed in 2013, directed by Maria Kosma and funded by the Phycha Foundation. Bioanthropological study: Eleni-Anna Prevedorou.

The town and harbour of Nea Styra is located on the western coast of southern Euboea in the sheltered Nea Styra Bay. The site lies at a strategic location on a narrow strait along the north-south and east-west axes of the maritime routes along the Euboean Gulf. The presence of a flourishing Early Bronze Age site on the coast of the Bay of Nea Styra has been known since the accidental discovery of three marble Early Cycladic figurines in the late 19th century (Kosma, 2010; Wolters, 1891) and detailed by field surveys in the area during the 20th century [e.g., see Sackett et al. (1966); Sampson (1980); Theocharis (1959)]. In 2009, three monumental built shaft graves of the Early Bronze Age were recovered during salvage excavations on the low hill of Gkisouri to the east of the modern settlement [see Kosma (2010)]. Thus, the recent excavations have marked Nea Styra as an important node in the southern

Euboean Gulf, right across from the roughly contemporaneous cemetery of Tsepi at Marathon in eastern Attica. The coexistence of both Helladic (mainland) and Cycladic features and influences in the burial practices at Nea Styra raise significant questions regarding its people and may suggest a regional network (Kosma, 2020b; Kosma, 2020a).

The extent of the cemetery is not known, though there was evidence for two more graves on the slopes, completely or partially destroyed by the construction of the road (Kosma, 2020b). The excavated graves were of monumental construction, all oriented differently. Grave 1 (Kosma, 2020b; Kosma, 2019) was lined with square schist slabs and consisted of a trapezoidal chamber and a short passage with three stone-paved steps leading to it (*dromos* or *prothyron*). The entrance was blocked by a schist slab and preserved two schist slabs as antae (pilasters) and a threshold. No covering or roof was preserved, though there was evidence for the presence of a large lintel made out of schist. Grave 3 was similar in construction, and both of these differ significantly from contemporaneous graves in Euboea and Boeotia, as well as from the cist graves observed in the Cyclades (Kosma, 2020b). Grave 2, however, shared more similarities with the rock-cut tombs in the Cyclades and Crete (Kosma, 2019). Overall, the Nea Styra graves showed great similarities with Early Helladic cemeteries in Attica, specifically with Tsepi at Marathon (Pantelidou-Gofa, 2005), Asteria at Glyfada (Kaza-Papageorgiou, 2019; Kaza-Papageorgiou, 2018) and Hagios Kosmas (Mylonas, 1959). A ritual deposit pit was discovered to the southeast of the complex of Graves 2 and 3 containing a large number of broken vessels (Kosma, 2020b). Analogous special deposits areas have been found at the Early Helladic cemetery of Tsepi in Marathon (Pantelidou-Gofa, 2005; Pantelidou-Gofa, 2008; Pantelidou-Gofa, 2016), as well as Asteria in Glyfada (Kaza-Papageorgiou, 2019; Kaza-Papageorgiou, 2018).

The material culture recovered in the three graves showed close stylistic affinities with the Cyclades, including a plethora of fragmented marble bowls and *pyxides*, marble palettes, bone tubes, obsidian blades, and figurines (Kosma, 2019). Specifically, a total of 16 specimens of marble Cycladic figurines were recovered (Kosma, 2019). Artifact typologies were representative of the “Keros-Syros culture,” dating the assemblage to the Early Helladic II (ca. 2700-2300 BC) (Kosma, 2019; Kosma, 2010; Kosma, 2020a).

The Nea Styra graves were used for the burial of multiple individuals. No articulated and/or *in situ* skeletons were recovered, suggesting their use as ossuaries. Skeletal remains, as well as artifacts were found fragmented, and at different depths. Skeletal elements, mainly skulls, from earlier burials were sometimes covered by schist slabs and/or layers of pebbles (Kosma, 2019). The specimens included in this study were recovered from Grave 1, which

produced the largest number of skeletal remains: an estimated number of 50 skulls, commingled with postcranial elements, according to the excavation notes.

Conservation completion of the skeletal remains was supported by the Institute for Aegean Prehistory. Final bioarchaeological analysis and publication are in progress. All of our work is a tribute to the late Maria Kosma and her untimely loss in 2015.

A total of 26 individuals from Grave 1 were sampled for DNA. We present genome-wide data and direct radiocarbon dates for the following five:

- NST001 (NST-1; Cranium 5, Layer 3, Tomb 1) is an adult individual represented by cranial and dental elements. Radiocarbon-dating on human bone (NST001.A): 3956±27 BP, 2568-2348 cal BC (95% probability), (ID: MAMS-41246, AMS, IntCal13).
- NST004 (NST-5; Cranium 18, Layer 4, Tomb 1) is an adult individual represented by cranial elements. Radiocarbon-dating on human bone (NST004.A): 3974±28 BP, 2574-2371 cal BC (95% probability), (ID: MAMS-41247, AMS, IntCal13).
- NST005 (NST-7; Cranium 25, Layer 4, Tomb 1) is an adult individual represented by cranial elements. Radiocarbon-dating on human bone (NST005.A): 3886±28 BP, 2465-2292 cal BC (95% probability), (ID: MAMS-41248, AMS, IntCal13).
- NST010 (NST-12; Cranium 41, Layer 5, Tomb 1) is an adult individual represented by cranial elements. Radiocarbon-dating on human bone (NST010.A): 4075±28 BP, 2851-2495 cal BC (95% probability), (ID: MAMS-41249, AMS, IntCal13).
- NST012 (NST-14; Skeletal Group 18, Layer 5, Tomb 1) is an individual represented by an isolated temporal bone. Radiocarbon-dating on human bone (NST012.A): 3933±28 BP, 2475-2350 cal BC (95% probability), (ID: MAMS-41250, AMS, IntCal13).

Tiryns, Argolid, Greece

Coordinates: 37.5995263, 22.7995951

Excavation(s): First excavation by the German Archaeological Institute, 1876-1929; latest excavation by the German Archaeological Institute and the Greek Archaeological Service, 2013-2018, directed by Prof. Joseph Maran and Dr. Alkestis Papadimitriou. A detailed history of the excavations is provided in Table 1. Bioanthropological research: Prof. Michael Schultz, Dr. Tyede Schmidt-Schultz.

1876 – 1929	German Archaeological Institute, excavations directed by Heinrich Schliemann, Wilhelm Dörpfeld, Georg Karo and Kurt Müller in the Upper, Middle and Lower Citadel, the Lower Town and the chamber tomb necropolis, as well as the first tholos tomb at the Prophitis Elias hill.
1957 – 1963	Greek Archaeological Service, excavations directed by Nikolaos Verdelis in the Lower Citadel
1957 – today	Greek Archaeological Service rescue excavations in the Lower Town
1965	Greek Archaeological Service and German Archaeological Institute, excavations directed by Nikolaos Verdelis in the Lower Citadel
1967 – 1974	German Archaeological Institute, excavations directed by Ulf Jantzen in the Lower Citadel and Western Lower Town
1976 – 1985	German Archaeological Institute, excavations directed by Klaus Kilian in the Northwestern Lower Town, Lower and Upper Citadel
1985	Greek Archaeological Service, excavations directed by Eleni Palaiologou in the second tholos tomb at the Prophitis Elias hill
1997 – 1998	German Archaeological Institute, excavations directed by Joseph Maran in the Upper Citadel
1999 – 2000	German Archaeological Institute and Greek Archaeological Service, excavations directed by Joseph Maran and Alkestis Papadimitriou in the Northeastern Lower Town
2000 – 2003	German Archaeological Institute, excavations directed by Joseph Maran in the Lower Citadel

2006 – 2010	German Archaeological Institute, excavations directed by Joseph Maran in the Western Lower Town
2013 – 2018	German Archaeological Institute and Greek Archaeological Service, excavations directed by Joseph Maran and Alkestis Papadimitriou in the Northwestern Lower Town

Table 1. History of excavations at the site of Tiryns.

The strongly fortified Mycenaean acropolis and palatial centre of Tiryns is situated about 1.5km from the present coast of the Bay of Nafplion (but only about 500m in the Early Bronze Age and 1km in the Late Bronze Age), where it perches on a narrow, rocky outcrop that reaches a height of up to 28m above sea level. The acropolis hill slopes from south to north, a topographic feature used during the Mycenaean palatial period (1400–1200 BC) to create a division into an Upper Citadel, a Middle Citadel, and a Lower Citadel by demarcating the limits of the different parts of the hill with strong, supporting walls built in so-called Cyclopean masonry. The acropolis was surrounded by an extensive settlement, the Lower Town, whose size during the different phases of occupation of the site is still difficult to determine.

During the Mycenaean period, extramural cemeteries used by inhabitants of Tiryns are attested by two tholos tombs and a chamber tomb necropolis (Rudolph, 1973; Müller, 1975) cut into the slopes of the Prophitis Elias hill, situated at a distance of about 1km to the east of the acropolis. In addition, an unusually high number of intramural burials without grave offerings dating to the Late Palatial and Postpalatial Mycenaean period (ca. 1300–1050 BC) was uncovered in the Lower Citadel (see below). Burials of the Early Iron Age (ca. 1050–700 BC) were encountered in different parts of the Lower Town next to house remains of that period, with the highest concentration of such burials having been found in the Western and Southwestern Lower Town. In the Byzantine period, burials are attested in the acropolis and different parts of the Lower Town.

Inhabited intermittently from the Middle Neolithic (ca. 5900–5400 BC), Tiryns became in the mid-3rd millennium BC an extensive and important Early Helladic settlement. Between ca. 1400 and 1200 BC, Tiryns was one of the major palatial centres of the Mycenaean palatial period in Greece and the most important harbour of the Peloponnesian Argolid region (Maran, 2010). The first palace built on the Upper Citadel in the course of the 14th century BC was replaced around 1250 BC by a second palace that was destroyed around 1200 BC in a major

conflagration (Kilian, 1988b). During the palatial period, the Lower Citadel was densely occupied, and its inhabitants were closely tied to the palace as administrators, artisans, workers or warriors. After the palatial period, Tiryns recovered much more rapidly than other sites from the setback caused by the destruction of the palace around 1200 BC and rose to the status of one of the foremost centres of the ensuing Postpalatial Aegean (ca. 1200–1050 BC) (Maran, 2016; Maran, 2015). After the Bronze Age, Tiryns remained continuously inhabited between the Early Iron Age and the early Classical period (ca. 1050–450 BC), but the focus of occupation shifted to the Lower Town, while the acropolis remained, at most, sparsely inhabited, with the Upper Citadel serving as a cult focus for the community of Tiryns. Little is known about Tiryns during the Byzantine period (ca. 7th – 14th cent CE), during which the occupation of the site seems to have been concentrated on the acropolis.

Based on the most recent anthropological-paleopathological analysis, at least 150 skeletal individuals buried without grave goods dating to the Late Palatial (ca. 1300–1200 BC; Late Helladic IIIB) or Postpalatial period (ca. 1200–1050 BC; Late Helladic IIIC) have been uncovered since the 1960s in open spaces or in ruins of former buildings of the Lower Citadel (Maran, 2008; Kilian, 1980). Despite the lack of grave goods, the burials do not have an irregular or haphazard appearance, since the deceased were carefully deposited on their backs in an extended position or lying on their side in a crouched (i.e., 'foetal') position in shallow pits. The burials without grave goods in the Lower Citadel markedly deviate from the Palatial and Postpalatial period burial tradition of interring the dead with grave goods in extramural chamber tombs dug into the slopes of hills around a settlement, such as the chamber tomb necropolis at the nearby Prophitis Elias hill. Kilian (1980) (Kilian, 1980) interpreted such Mycenaean intramural burials of the Late Palatial period as those of members of low social status groups within the palatial society to whom the right was denied to be buried with grave goods in such chamber tombs. This interpretation may very well be correct, but fact is that the practice of depositing the dead without grave goods continued after the destruction of the palace, when the social conditions must have been considerably different to those from the Palatial period. In the early 12th century BC, it seems that the entire northernmost part of the Lower Citadel was even temporarily transformed into a burial ground for such burials (Maran, 2008). Therefore, we may be dealing with funerary traditions linking the Palatial and Postpalatial period that were not only practiced because burial in chamber tombs was prohibited, but because there were social groups that did not identify with the normative funeral traditions and wanted to bury their dead differently.

In contrast to these Mycenaean burials, the ones of the Early Iron Age were usually placed in stone cists, storage vessels or in pits and were usually furnished with grave offerings (Papadimitriou, 2003). Single burials or groups of burials of the Byzantine period, some of them with grave offerings, were found in grave pits and rarely in stone cists in the Lower Citadel and different parts of the Lower Town.

The ongoing anthropological-paleopathological study by Michael Schultz and Tyede H. Schmidt-Schultz began in 2011 and focuses on the human skeletal remains from burials dating between the Mycenaean and the Byzantine period that have been uncovered since the 1960s in different parts of the acropolis and Lower Town. Unfortunately, due to a lack of available human skeletal remains from earlier excavations, it was not possible to include any of the burials in the extramural chamber tomb necropolis and tholos tombs at Prophitis Elias hill in the study. The anthropological-paleopathological study aims to determine the sex, age, height, type of constitution and handedness, as well as finding evidence of the living conditions, diseases and, when possible, the cause of death, in order to demographically characterize this segment of the population of Tiryns (Schultz and Schmidt-Schultz, 2015). The identified skeletal individuals were differentiated by the anthropologists through a numbering system consisting of the name of the excavator and a consecutive number starting with “1” for each excavator (cf. “Kilian 035”).

Of the 16 individuals from the Mycenaean contexts sampled for this study, only three produced genome-wide data that are included in the genetic analyses. All of them were directly radiocarbon-dated:

- TIR001 (Burial 5/03; LXIII35/03, VIF/VIG; Maran 010) is a male individual approximately 26-30 years old. The skeleton was found in 2003 in the Northwestern Lower Citadel lying on its back in an extended position and without grave offerings in an archaeological context dating to the Late Palatial period (Maran, 2008). The individual had a normally developed musculoskeletal system and suffered from chronic diseases of the upper and the lower respiratory tract. Radiocarbon-dating of human bone: 3053±24 BP, 1403–1229 cal BC (95% probability), (ID: MAMS-47534, AMS, IntCal20).
- TIR002 (Grave 7/1; LXII37/8 Nr.3; Nr. 1015; Kilian 024) is an individual ca. 40-55 years old represented by the partially preserved upper body of the skeleton found in 1977 in the Northwestern Lower Citadel. It was part of a group of burials without grave offerings in an archaeological context dating either to the Late Palatial period (LH IIIB

Developed or Final) or the early Postpalatial period (Kilian, 1979; Damm-Meinhardt, 2015) (LH IIIC Early). The individual had a very well-developed musculoskeletal system and suffered from chronic diseases of the upper respiratory tract. Despite the absence of important diagnostic features (e.g., the pelvis, femur, tibia, radius and ulna), a morphological sex diagnosis could be established based on the very pronounced male features of the skull (i.e., muscle marks on the occipital bone) and the humerus. Interestingly though, genetic sexing conducted on a tooth sample determined it was a female. Radiocarbon-dating of human tooth (TIR002.A): 3044±23 BP, 1394-1222 cal BC (95% probability), (ID: MAMS-47535, AMS, IntCal20).

- TIR010 (Grave 18; LXII44/29.30, XVIIa Nr.1048; Kilian 032) is a male individual approximately 45-50 years old. The skeleton was found in 1982/1983 lying on its back in an extended position in the Southwestern Lower Citadel as part of a group of burials without grave offerings in an archaeological context dating to the Late Palatial period (Kilian, 1988a) (LH IIIB Middle or Developed). The individual had a very well-developed musculoskeletal system and suffered from chronic diseases of the upper respiratory tract. Radiocarbon-dating of human bone: 3116±26 BP, 1440-1299 cal BC (95% probability), (ID: MAMS-42114, AMS, IntCal13).

Of the five individuals from the Iron Age (Protogeometric and Geometric) contexts sampled for this study, only one produced genome-wide data.

- TIR008 (Burial 1/14, Individual 1; LII25/58 II/II A & Ofl. II in. Nr. 134/14 and LII 25/58 I A; Maran 067) is represented by a well-preserved skeleton of a male who died at the age of 25-30 years, possibly as late as 35 years. This young male was of average height and had a medium physical constitution. The bones of this individual were found in 2014 in the Northwestern Lower Town in a concentration of redeposited human bones and three miniature vessels of the Geometric period immediately to the west of the covering slabs of Grave 2/14 containing burials of the Geometric period (Maran and Papadimitriou, 2016). The redeposited bones are likely to represent the collected remains of earlier, removed internments that dated to the Geometric period and possibly also the Protogeometric period, since the latter is represented by pottery also found in the vicinity of the same grave. Radiocarbon-dating of human bone: 2765±26 BP, 991-835 cal BC (95% probability), (ID: MAMS-42113, AMS, IntCal13).

AEGEAN ISLANDS

Koukounaries, Paros, Greece

Coordinates: 37.126595, 25.208784

Excavation: Archaeological Society at Athens, 1976-1992, directed by Prof. Demetrius U. Schilardi. Excavations by the "Paros Excavations" team were carried out under the auspices of the Archaeological Society at Athens, and subsequent studies and research on Koukounaries were supported by the Institute for Aegean Prehistory, the A.G. Leventis Foundation, the National Geographic Society and private donors (see acknowledgements in Schilardi, 2016). Bioanthropological study: Eleni-Anna Prevedorou.

The study and final publication of Koukounaries were interrupted by the sudden death of Dimitrius Schilardi in 2020. We are all making efforts to continue his devoted work. The volume on the Mycenaean pottery is now in press with Archaeopress by Robert Koehl. The publication of the Koukounaries excavations and related materials is currently coordinated by Prof. Alexandros Mazarakis Ainian under the auspices of the Archaeological Society at Athens.

The site of Koukounaries is located on a rocky hill (about 75m above sea level) by the Naoussa Bay, on the northern coast of Paros Island. The panoramic view over the bay and the sea, combined with the difficult access to the summit of the hill, offer strategic advantages. The site forms a palimpsest of occupation phases ranging from the Late Neolithic to Hellenistic times (Schilardi, 2016; Schilardi, 1999).

Late Neolithic (5th millennium BC) and successive Early Cycladic II finds (ca. 2700-2300 BC) were recovered on the Lower Plateau. In the early 12th century BC, the hill was fortified with a Cyclopean wall, transforming it into a citadel, including a Mycenaean palatial "mansion" and numerous artifacts of wealth and prestige; this citadel was destroyed in ca. 1150 BC. This destruction did not mark the end of habitation at the settlement, though, and both cult and domestic buildings are present from the Protogeometric through the early Archaic periods, with a temple of Athena continuing in use until the 3rd century BC.

The Mycenaean phase of Koukounaries Hill is of great importance. The primary Mycenaean phase dates to the LH IIIC – Middle (around 1175-1150 BC) (Schilardi, 2016). The mansion was built in LH IIIC Middle Developed, i.e., after the destruction of the mainland Mycenaean citadels, and destroyed in LH IIIC Middle Advanced, perhaps as the result of a siege, as suggested by the piles of sling bullets, arrowheads and pikes found in the destruction

level. A siege might thus explain the circumstances of the intramural burial in storeroom 2 (E 1; see below). The building, which was originally two-storied, collapsed, thus preserving a large number of storage areas filled with everyday vessels and storage jars (*pithoi*), as well as prestigious artifacts and metal finds. A layer of ash, in places reaching over 1m in thickness, covered the floors of the mansion. Skeletal human and faunal remains (including horse remains) were identified in the lower stories (basement and ground floor) of the building. This led to the hypothesis that the skeletal remains recovered belonged to people who sought refuge in the citadel along with animals during a siege and probably died in the collapse of the building during the conflagration (Schilardi, 2016). After the destruction of the mansion, small areas of the building were cleared and re-occupied in LH IIIC Late. Additionally, a small cave located on the upper slope of the hill, just outside the mansion, contained the skeletal remains of a male, probably dated to LH IIIC Middle.

The nature of the destruction, population mobility and the role of Koukounaries during late Mycenaean times remain important archaeological questions under investigation. We should note that a Mycenaean cemetery with chamber tombs was identified in the nearby narrow valley to the west called Loggos, associated with the acropolis (Schilardi, 2016). However, the tombs were completely emptied, and no skeletal or other remains were recovered. Thus, all skeletal material discussed here comes from the citadel. A subset of the human skeletal remains from the Koukounaries acropolis were studied by Sara Bisel in 1981 but were not published [see Schilardi (1999)]. Bioarchaeological and biogeochemical analyses and the publication of the human skeletal assemblage recovered at Koukounaries are in progress. The skeletal information reported here is based only on preliminary observations. Sampling and isotopic analyses are funded by the M. H. Wiener Foundation.

Of the seven individuals from Koukounaries sampled for this study, four produced genome-wide data and are included in the genetic analyses:

- KUK001 (KOU-1) is a middle adult female represented by a nearly complete skeleton whose burial was dug into the floor of the second storeroom (Schilardi, 2016). The skeleton was found in a tightly contracted position, lying on the right side. An engraved, discoid sealstone worn as a pendant was recovered with the skeleton (Schilardi, 2016). Preliminary skeletal evidence includes sharp-force cranial trauma, antemortem tooth loss and extensive osteoarthritic changes.
- KUK002 (KOU-3) is a young adult male represented by a nearly complete skeleton, buried in the cave at the northeastern edge of the Upper Plateau, accessed by a stone

staircase (Schilardi, 1999; Schilardi, 2016). The skeleton was placed in a supine position, with the lower legs bent, possibly due to a lack of space (Schilardi, 1999). It was accompanied by the ceramic figurine of a small horse. Sara Bisel reported hypertrophy of the adductor tubercles (and thus of the muscles) of the femora and attributed this to habitual horse-back riding (Schilardi, 1999).

- KUK005 (KOU-B) is an infant, approximately 3 years old, represented by an incomplete skeleton (Plateau, D2, Layer 8, 1982).
- KUK006 (KOU-D) is an infant, approximately 2 years old, represented by an incomplete skeleton (Plateau, D5, 1981).

Lazarides, Aegina, Greece

Coordinates: 37.721203, 23.505420

Excavation(s): B΄ Ephorate of Antiquities, 1979-1980, directed by Cl. Eustratiou; Department of Archaeology and History of Art of the University of Athens, 2002-present, directed by Prof. Naya Polychronakou-Sgouritsa. Bioanthropological study: Eleni-Anna Prevedorou.

The Mycenaean settlement at Lazarides, in the neighbourhood of the homonymous semi-mountainous modern village, is located on a high plateau on eastern Aegina, 10km from Kolonna and an hour's walk from the the bay of Kylindras, where finds suggest another Mycenaean community (Supplementary Figure 31). Although not visible from the sea, it had an excellent view of the Saronic Gulf and the adjacent areas of Attica and the northwestern Peloponnese (Sgouritsa and Salavoura, 2014). Research started in 1979 as a salvage excavation of the built chamber tomb cemetery and of the settlement in 1980, continuing to the present day. In 2002, the Department of Archaeology and History of Art of the University of Athens undertook a field survey and the systematic excavation of this site. Since then, a great part of the settlement has been discovered (Supplementary Figure 32), and the material, old and recent, from the cemetery has been published (Eustratiou and Polychronakou-Sgouritsa, 2016).

The site thrived during the period from LH IIIA2 to the end of LH IIIB/beginning of LH IIIC, judging by the pottery from the settlement and the neighbouring cemetery about 200m away. The earliest evidence dates to the Middle Helladic (MH) III/LH I period (Sgouritsa, 2010; Sgouritsa, 2021b), and it is highly probable that people from Kolonna founded the community at Lazarides. Besides agro-pastoral activities, the inhabitants of the site maintained

contacts with Attica, the northwestern Peloponnese (Sgouritsa, 2015), the Cyclades, the southeastern Aegean and Crete (Eustratiou and Polychronakou-Sgouritsa, 2016): they participated in exchange networks involving, among other goods (Sgouritsa and Salavoura, 2014; Sgouritsa, 2021b), storage and, mostly, cooking vessels, that reached to Thessaly (Sgouritsa, 2021b; Lis, 2012), while metal artifacts of bronze, lead, silver and iron (Tselios, 2016; Eustratiou and Polychronakou-Sgouritsa, 2016), as well as jewellery (Sgouritsa, 2012), point to an unexpected affluence. Moreover, weights found in the settlement imply relations with foreign traders, possibly Cypriots (Sgouritsa, 2021a).

Human remains come from both the cemetery, with male, female and infant interments, and the settlement (Prevedorou, 2016), where infant burials were discovered in several rooms of different uses. The latter were either simply deposited in the corners of rooms (Supplementary Figure 33), wherein one burial was enclosed by a wall (Supplementary Figure 34), or in small cist graves (Supplementary Figure 35). Sometimes they were supplied with offerings, such as a necklace, a figurine or even miniature bronze tools (probably toys?). Besides the intramural infant burials, very few adults seem to have been buried in the settlement during the last phase of its inhabitation, although the cemetery was still in use at this time.

The human skeletal remains included in this study have been published with the remains excavated before 2013 (Prevedorou, 2016). The bioarchaeological, biogeochemical, and radiocarbon analyses of the Lazarides skeletal assemblage are in progress by Eleni-Anna Prevedorou, funded by the Institute for Aegean Prehistory (INSTAP). Of the 22 individuals from Lazarides sampled for this study, five produced genome-wide data and are included in the genetic analyses. All of them were directly radiocarbon-dated, and all come from within the settlement (Supplementary Figure 32).

- LAZ017 (LZR-23) is probably an adult individual represented by a fragmented petrous bone. It was found in a bone concentration at the southern wall/entrance of room 1 of the complex located in the Papadimitris Plot. In total, the remains of at least three mature individuals and one young child approximately 3 years old (± 1 year) could be identified in a pile of mingled bones and teeth. The early date of LAZ017 suggests this was likely a secondary treatment of earlier burials, a relocation carried out for unknown reasons or a burial of several persons. Radiocarbon-dating of human bone (LAZ017.A): 4171±25 BP, 2881-2635 cal BC (95% probability), (ID: MAMS-47523, AMS, IntCal20).

- LAZ018 (LZR-24) is a neonate, represented by cranial and postcranial elements found in Room 1 of the complex, used for storage and dated to the LH IIIA2/B1 period. The burial was deposited in the southwestern corner without offerings (Supplementary Figure 31) and with the head to the east. Radiocarbon-dating of human bone (LAZ018.A): 3097±21 BP, 1424-1293 cal BC (95% probability), (ID: MAMS-47524, AMS, IntCal20).
- LAZ019 (LZR-25) is an infant approximately 1 year old (12-16 months). The relatively complete skeleton was found in a cist tomb without a slab cover (0.6m E-W x 0.35m N-S) in space 5a, which was probably an open area, in 2010. It was provided with a necklace (Supplementary Figure 33). It may be dated to the LH IIIB period, as it was discovered in close proximity to a wall reconstructed at that time. Cranial and postcranial involvement suggest severe, prolonged symptoms of genetic anemia. The co-presence of infectious processes (e.g., parasitic) is also possible. The skeletal evidence and the clinical literature indicate that the infant had been severely ill for several months, if not all, of his young life [see Prevedorou (2016)]. Radiocarbon-dating of human bone (LAZ019.A): 3045±27 BP, 1398-1221 cal BC (95% probability), (ID: MAMS-47525, AMS, IntCal20).
- LAZ020 (LZR-26) is an infant approximately 12-16 months old represented by cranial and postcranial elements. It was found in a bone concentration in the southeastern corner of Room 5 of the Papadimitris Plot excavated in 2011 and contained the remains of at least five more individuals: one preterm, three perinates, and an infant about 6-9 months old, along with a few faunal remains. It was provided with a typical Psi- type figurine, dated to the LH IIIB1 period. Radiocarbon-dating of human bone (LAZ020.A): 3055±21 BP, 1403-1233 cal BC (95% probability), (ID: MAMS-47526, AMS, IntCal20).
- LAZ021 (LZR-27) is a perinatal infant (ca. 36-38 weeks old) represented mainly by cranial elements. It was found in Room 6 of the Papadimitris Plot, excavated in 2013, under a platform built in the northwestern corner during its final reorganization in the LH IIIB2 period. It may be dated to the LH IIIA2/B1 period. Radiocarbon-dating of human tooth (LAZ021.A): 3311±26 BP, 1626-1508 cal BC (95% probability), (ID: MAMS-49526, AMS, IntCal20).

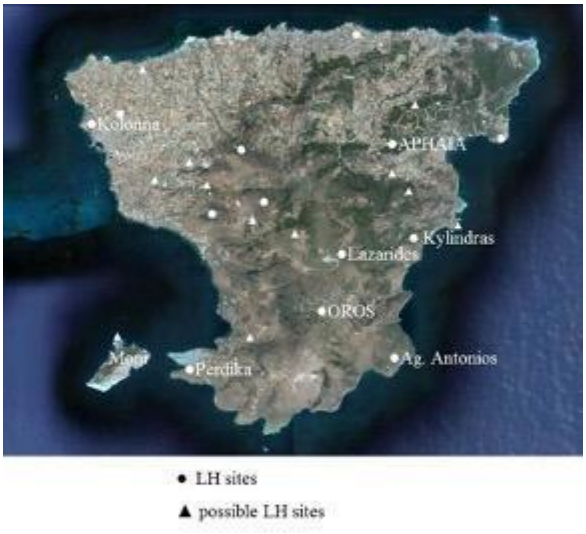

Supplementary Figure 31. Map of Aegina with the location of Lazarides and other Late Helladic (LH) sites.

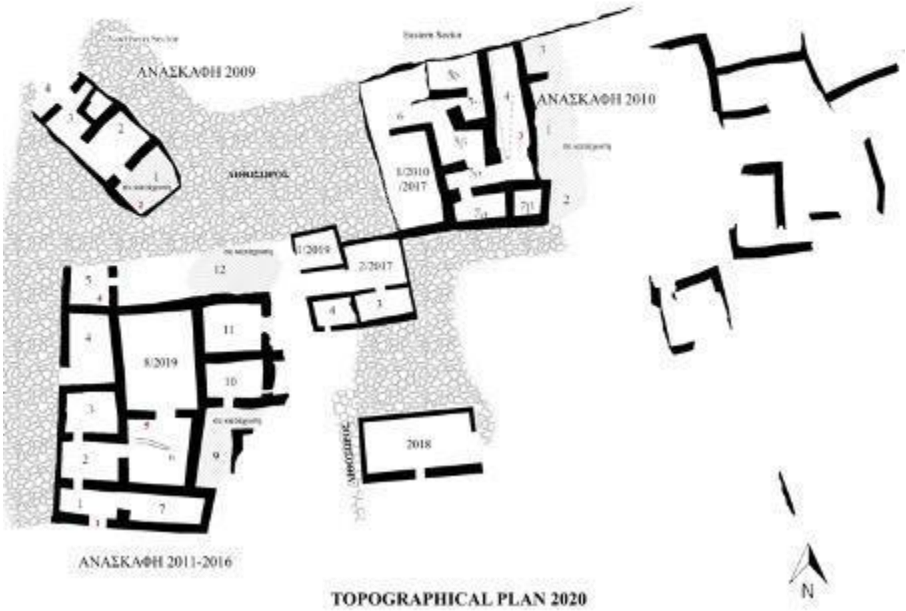

Supplementary Figure 32. Topographic plan of Lazarides with the locations of the individuals presented in this study marked in red.

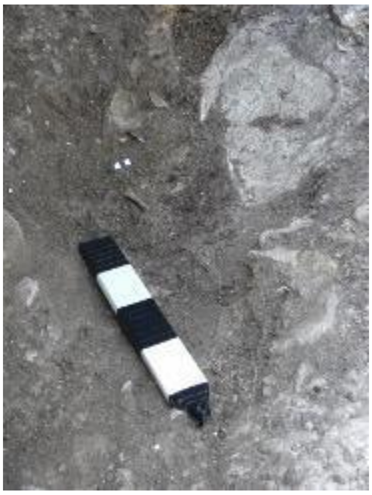

Supplementary Figure 33. Infant burial in the southwestern corner of Room 1 (excavation 2009).

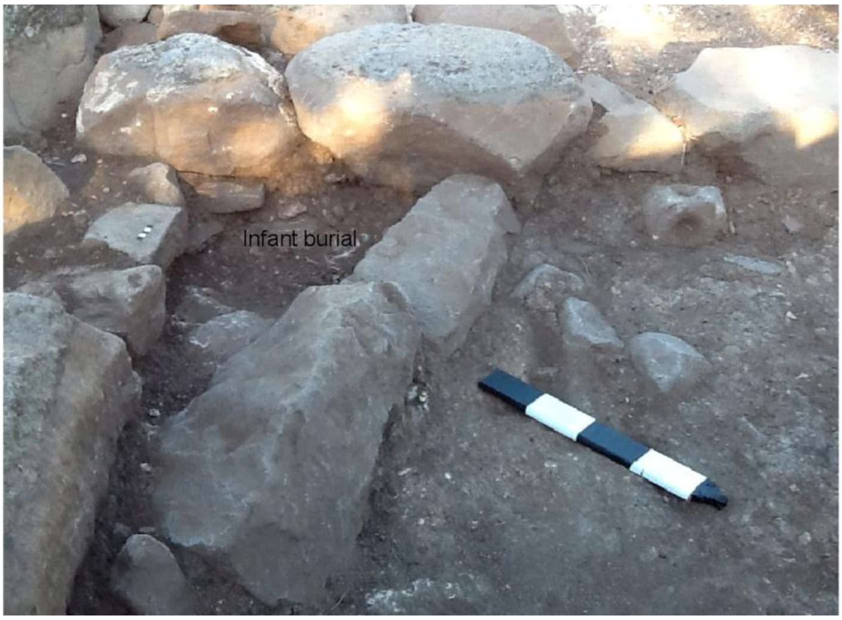

Supplementary Figure 34. Infant burial in the southwestern corner of Room 7 (2013).

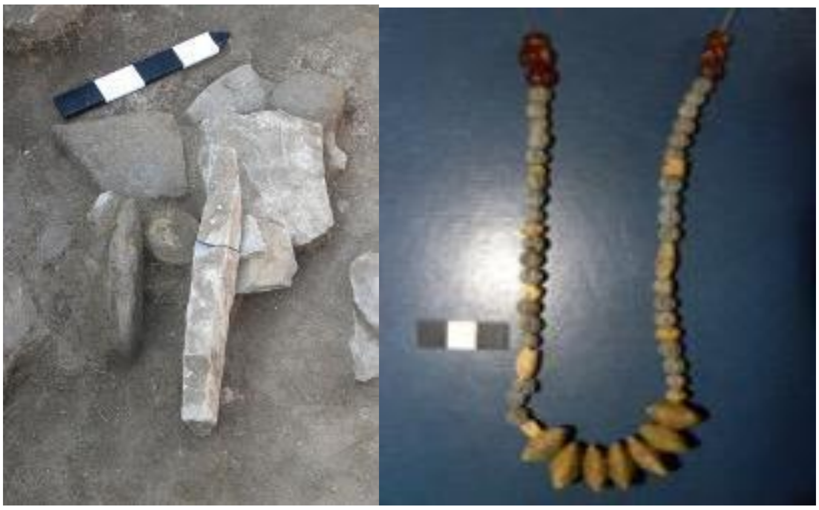

Supplementary Figure 35. Infant burial in a cist grave (excavation 2010) provided with a necklace.

References

Agelarakis, A. P. 1996. A Field and Laboratory Manual for Archaeologists, for the Excavation, Documentation, and Preservation of Human Osseous Remains. Ariadne, 189-247.

Agelarakis, A. P. 2014. On the Preservation and Conservation of Archaeologically Recovered Anthropological Remains: A Brief Communication to Younger Colleagues. In: Korka, E. (ed.) The Protection of Archaeological Heritage in Times of Economic Crisis. Cambridge Scholars Publishing.

Agelarakis, A. P. & Kanta, A. 2019. Intra-group dynamics, glimpses of labor diversity and specialization, and evidence of incipient social stratification in Neolithic Crete: Reflections from the Aposelemis Burial Ground. 12th International Congress of Cretan Studies, 21-25 September 2016 Heraklion. 1-17.

Agelarakis, A. P. & Kanta, A. 2020. The Neolithic Cemetery of Aposelemis. In: Karanastasi, P., Tsigounaki, A. & Tsigonaki, C. (eds.) Proceedings 4th Archaeological Work in Crete Congress. Hellenic Ministry of Culture & Athletics, Antiquities Authority of Rethymnon, University of Crete.

Alexiou, S. 1953. Anaskafai en Katsamba [Ανασκαφαί εν Κατσαμπά; Excavations at Katsambas]. ΠΑΕ / Prakt

Andreadaki-Vlazaki, M. 1992. Oikopedo Emm. Lentari - H. Manolakaki [Οικόπεδο Εμμ. Λεντάρη – Χ. Μανωλικάκη]. ArchDelt (Αρχαιολογκόν Δελτίον), 42 (1987), 556-557.

Andreadaki-Vlazaki, M. 1997. La nécropole du Minoen Récent III de la ville de La Canée. In: Driessen, J. & Farnoux, A. (eds.) La Crète mycénienne.

Andreadaki-Vlazaki, M. 2003. Odos Malinou kai Kritovoulidou (oikopedo K. Rovithaki) [Οδός Μαλινού και Κριτοβουλίδου (οικόπεδο Κ. Ροβιθάκη)]. ArchDelt (Αρχαιολογικόν Δελτίον), 52 (1997), 1003-1042.

Andreadaki-Vlazaki, M. 2004. Odos Kanevaro [Οδός Κανέβαρο]. ArchDelt (Αρχαιολογκόν Δελτίον), 53 (1998), 856-858.

Andreadaki-Vlazaki, M. 2010. Khania (Kydonia). In: Cline, E. H. (ed.) The Oxford Handbook of the Bronze Age Aegean (ca. 3000-1000 BC). Oxford University Press.

Andreadaki-Vlazaki, M. 2011. To palimpsiston tis arhaias Kydonias [Το «παλίμψηστον» της αρχαίας Κυδωνίας]. Proceedings of 10th International Congress of Cretan Studies (Chania 1-8 October 2006) [Πεπραγμένα του 10ου Διεθνούς Κρητολογικού Συνεδρίου (Χανιά, Οκτ. 1-8, 2006)]. The Philological Association "Chrysostomos".

Andreadaki-Vlazaki, M. 2015. Sacrifices in LMIIIB: Early Kydonia Palatial Centre. Pasiphae, 9, 27-40.

Andreadaki-Vlazaki, M. 2018. Das Menschenopfer von Kydonia. Besanftigung zorniger Gotter? Mykene. Die sagenhafte Welt des Agamemnon. wbg Philipp von Zabern.

Andreadaki-Vlazaki, M. 2022a. Recent Evidence from the 'Katre 1' Excavation, Kastelli Hill, Khania. Pasiphae, 14, 25-36.

Andreadaki-Vlazaki, M. 2022b. The Arrival of the Mycenaeans at Kydonia: Ancient Tradition and Archaeological Evidence. In: D'Agata, A. L., Girella, L., Papadopoulou, E. & Aquini, D. G. (eds.) Supplemento 2 - One State, Many Worlds. Crete in the Late Minoan II-IIIA2 Early Period. Edizioni Quasar.

Andreadaki-Vlazaki, M. & Protopapadaki, E. 2009. '"Kouklaki" excavation (73-77 Igoumenou Gabriel St. In: Andreadaki-Vlazaki, M. (ed.) A Tour to Sites of Ancient Memory. Ministry of Culture and Tourism – 25th Ephorate of Prehistoric and Classical Antiquities.

Betancourt, P. P. 2014. Hagios Charalambos: A Minoan Burial Cave in Crete: I. Excavation and Portable Objects, Philadelphia, INSTAP Academic Press.

Betancourt, P. P., Davaras, C., Stravopodi, E., Karkanas, P., Langford-Verstegen, L., Mubly, J. M., Hickman, J., Dierckx, H. M. C., Ferrence, S. C., Reese, D. S., et al. 2008. Excavations in the Hagios Charalambos Cave, a Preliminary Report. Hesperia, 77, 539-605.

Bonga, L. 2019. Neolithiki keramiki apo to spilaio Pelekiton [Νεολιθική κεραμική από το σπήλαιο Πελεκητών; Neolithic ceramics from the Pelekita cave]. In: Mitsotaki, C. & Tzedaki-Apostolaki, L., eds. Proceedings of 12th International Congress of Cretan Studies, 2016 Heraklrion. Heraklion: Εταιρία Κρητικών Ιστορικών Μελετών - Ιστορικό Μουσείο Κρήτης.

Bonga, L. & Ferrence, S. In press. Pelekita Cave, Kato Zakros, Crete: Late Neolithic and Minoan Interactions with the Dodecanese. Southeast Aegean / Southwest Coastal Anatolian Region International Conference (SASCAR). Material Evidence and Cultural Identity: 1. Early and Middle Bronze Age, 12-14 May 2016 Italian Archaeological School at Athens.

Bunni, A., Lagarce, J., Lagarce, E., Saliby, N., J., L. & P., B. 1998. Ras Ibn Hani, fouilles 1979-1995. Synthèse preliminaire I. Le palais nord du bronze recent., Beyrouth, Institut français d'archéologie du Proche-Orient.

Casselman, C., Fuchs, M., Ittameier, D., Maran, J. & Wagner, G. A. 2004. Interdisziplinäre landschaftsarchäologische Forschungen im Becken von Phlious, 1998-2002. Archäologischer Anzeiger, 1, 1-58.

Damm-Meinhardt, U. 2015. Baubefunde und Stratigraphie der Unterburg (Kampagnen 1976-1983). Die mykenische Palastzeit (SH III B2) und beginnende Nachpalastzeit (Beginn SH III C), Dr. Ludwig Reichert Verlag.

Davaras, C. 1976. Guide to Cretan Antiquities, New Jersey, Park Ridge.

Davaras, C. 1983. Hagios Charalambos (Getontomouri) Lasithiou [Άγιος Χαράλαμπος (Γεροντομουρί) Λασιθίου]. ArchDelt (Αρχαιολογικόν Δελτίον), 31, 379-380.

Davaras, C. 1986. Proimes minoikes sfragistikoi daktylioi apo to spilaio Gerontomouri Lasithiou [Πρώιμες μινωικές σφραγιστικοί δακτύλοι από το σπήλαιο Γεροντομουρί Λασιθίου]. ArchEph (Αρχαιολογική Εφημερίς), 9-48.

Davaras, C. 1989a. Spilaio Gerontomouri [Σπήλαιο Γεροντομουρί; Gerontomouri Cave]. ArchDelt (Αρχαιολογικόν Δελτίον), 38, 375.

Davaras, C. 1989b. Spilaio Hagiou Charalambous [Σπήλαιο Αγίου Χαραλάμπους; Hagios Charalambos Cave]. ArchDelt (Αρχαιολογικόν Δελτίον), 37, 387-388.

Davaras, C. 2015. The Elusive site of the Primary Burials of the Hagios Charalambos Cave: A Speculative Scenario. In: Betancourt, P. P., Davaras, C. & Stravopodi, E. (eds.) Hagios Charalambos. A Minoan Burial Cave in Crete II, The Pottery. INSTAP Academic Press.

Dawkins, R. M., Hawes, C. H. & C., B. R. 1904-1905. Excavations at Palaikastro. IV.

Douka, K., Efstratiou, N., Hald, M. M., Henriksen, P. S., Karetsou, A. & (2017) “Dating Knossos and the arrival of the earliest Neolithic in the southern Aegean, A. C. U. P., 91(356), pp. 304–321. doi: 10.15184/aqy.2017.29. 2017. Dating Knossos and the arrival of the earliest Neolithic in the southern Aegean. Antiquity, 93, 304-321.

Efstratiou, N., Karetsou, A. & Ntinou, M. (eds.) 2013. The Neolithic Settlement of Knossos in Crete: New Evidence for the Early Occupation of Crete and the Aegean Islands, INSTAP Academic Press.

Eustratiou, K. & Polychronakou-Sgouritsa, N. 2016. To Mykinaiko nekrotafeio stous Lazarides Aiginas [Το Μυκηναϊκό νεκροταφείο στους Λαζάρηδες Αίγινας; The Mycenaean cemetery at Lazarides, Aegina]. ArchDelt (Αρχαιολογικόν Δελτίον), 65-66, 2010-2011, 1-162.

Evans, A. J. 1901. The Neolithic Settlement at Knossos and its Place in the History of Early Aegean Culture, London, Royal Anthropological Institute of Great Britain and Ireland.

Evans, A. J. 1921. The Palace of Minos at Knossos: The Neolithic and Early and Middle Minoan Ages, London, Macmillan and Co., Limited.

Evans, A. J. 1928. The Palace of Minos at Knossos: A comparative account of the successive stages of the early Cretan civilization as illustratted by the discoveries, London, Macmillan and Co., Limited.

Evans, J. D. 1964. Excavations in the Neolithic Settlement of Knossos, 1957–60. Part I. The Annual of the British School at Athens. Cambridge University Press.

Galanidou, N. & Manteli, K. 2008. Neolithic Katsambas Revisited: the Evidence from the House. In: Isaakidou, V. & Tomkins, P. D. (eds.) Escaping the Labyrinth: The Cretan Neolithic in Context. Oxbow Books.

Hachtmann, V. 2013. The Bronze Age settlement at Aidonia. In: Kissas, K. & Niemeier, W.-D. (eds.) The Corinthia and the Northeast Peloponnese. Topography and History from Prehistoric Times until the End of Antiquity.

Hallager, B. P. & McGeorge, P. J. P. 1992. Late Minoan III burials at Khania. The tombs, finds and deceased in Odos Palama, Göteborg, Åström.

Hallager, E. & Andreadaki-Vlazaki, M. 2017. The Greek-Swedish-Danish Excavations 2014: A short preliminary report. Proceedings of the Danish Institute at Athens, 8, 280-292.

Hallager, E. & Hallager, B. P. (eds.) 2003, 2011 and 2016. The Greek-Swedish Excavations at the Agia Aikaterini Square, Kastelli, Khania 1970-1987 and 2001.

Istituto per gli studi micenei ed, e.-a. 1989. Scavi a Nerokourou, Kydonias, Roma, Edizioni dell'Ateneo.

Kakavoyannis, E. 1999-2001. Mykinaiko nekrotafeio sto lofo Fouresi tou Dimou ton Glykon Neron Attikis [Μυκηναϊκό νεκροταφείο στο λόφο Φούρεσι του Δήμου των Γλυκών Νερών Αττικής; Mycenaen cemetery at the Fouresi hill of Glyka Nera in Attica]. Athens Annals of Arcaheology, 32-34, 55-70.

Kanta, A., Ferrence, S. & Bonga, L. in press. Spilaio Pelekiton Zaktou 2014, 2015. Prokatarktiki Ekthesi ton ergasion. [Σπήλαιο Πελεκητών Ζάκρου 2014, 2015. Προκαταρκτική Έκθεση των εργασιών; Pelekita Cave at Zakros 2014, 2015. A preliminary report]. 4th Meeting for the Archaeological Work in Crete, 2016 Rethymnon.

Kanta, A. & Serpetsidaki, I. To ergo tis 23 Ephoreias Proïstorion kai Klassikon Arhaiotiton kata ta eti 2011-2013 [Το εργο της ΚΓ΄ Εφορείας Προϊστορικών και Κλασικών Αρχαιοτήτων κατά τα έτη 2011 – 2013; The work of the 23st Ephorate of Prehistoric and Classical Antiquities between 2011 and 2013]. In: Karanastasi, P., Tzigoudaki, A. & Tsigonaki, C., eds. 3rd Meeting for the Archaeological Work in Crete, 2013 Rethymnon. 53-68.

Kataki, E. 2014. Symvoli ton odon I. Sfakianaki 27 kai Platonos (oikopedo Melefaki) [Συμβολή των οδών Ι. Σφακιανάκη 27 και Πλάτωνος (οικόπεδο Μαλεφάκη)]. ArchDelt (Αρχαιολογικόν Δελτίον), 63 (2008), 1154-1155.

Kaza-Papageorgiou, K. 2018. Asteria Glyfadas. Anthropini Parousia stin proistoriki epohi [Asteria at Glyfada. Human presence at prehistoric times]. Arhaiologia kai Tehnes, 128, 30-41.

Kaza-Papageorgiou, K. 2019. Cycladic-type figurines from the Early Helladic cemetery of Asteria at Glyfada, Attica. In: Marthari, M., Renfrew, C. & Boyd, M. J. (eds.) Beyond the Cyclades. Early Cycladic Sculpture in Context from Mainland Greece, the North and East Aegean. Oxbow Books.

Kilian, K. 1979. Ausgrabungen in Tiryns 1977. Bericht zu den Ausgrabungen. Archäologischer Anzeiger, 379–411.

Kilian, K. 1980. Zum Ende der mykenischen Epoche in der Argolis. Jahrbuch des Römisch-Germanischen Zentralmuseums Mainz, 27, 166–195.

Kilian, K. 1988a. Ausgrabungen in Tiryns 1982/83. Bericht zu den Ausgrabungen. Archäologischer Anzeiger, 105–151.

Kilian, K. 1988b. Mycenaeans up to Date: Trends and Changes in Recent Research. In: French, E. B. & Wardle, K. A. (eds.) Problems in Greek Prehistory: Papers Presented at the Centenary

Conference of the British School of Archaeology at Athens, Manchester, April 1986. Bristol Classical Press.

Kontopodi, D. forthcoming. Kannia Gortys. ArchDelt (Αρχαιολογικόν Δελτίον).

Kosma, M. 2010. New Early Cycladic figurine at Nea Styra. Mediterranean Archaeology and Archaeometry, 10, 29-36.

Kosma, M. 2019. Cycladic Marble Figurines from the Early Bronze Age Cemetery at Nea Styra, Euboea. In: Marthari, M., Renfrew, C. & Boyd, M. J. (eds.) Beyond the Cyclades. Early Cycladic Sculpture in Context from Mainland Greece, the North and East Aegean. Oxbow Books.

Kosma, M. 2020a. Nea stoiheia gia tis sheseis Attikis kai Euboias kata tin Proimi Epohi tou Halkou, mesa apo ta dedomena tou protoelladikou nekrotafeiou sta Nea Styra Euboias [Νέα στοιχεία για τις σχέσεις Αττικής και Εύβοιας κατά την Πρώιμη Εποχή του Χαλκού, μέσα από τα δεδομένα του πρωτοελλαδικού νεκροταφείου στα Νέα Στύρα Ευβοίας; New insights for the relations between Attica and Euboea during the Early Bronze Age via the evidence from the Early Helladic cemetery at Nea Styra in Euboea]. In: Papadimitriou, N., Wright, J. C., Fachard, S., Polychronakou-Sgouritsa, N. & Andrikou, E. (eds.) Athens and Attica in Prehistory: Proceedings of the International Conference. Athens, 27-31 May 2015. Archaeopress.

Kosma, M. 2020b. To protoelladiko nekrotafeio sti thesi Gkisouri sta Nea Styra Euboias [Το πρωτοελλαδικό νεκροταφείο στη θέση Γκισούρι στα Νέα Στύρα Εύβοιας; The Early Helladic Cemetery at Gkisouri in Nea Styra of Euboea]. In: Mazarakis Ainian, A. (ed.) Archailogiko Ergo Thessalias kai Stereas Elladas, 5 (2015): Praktika Epistimonikis Synantisis, Volos, 26 Fevrouariou – 1 Martiou 2015. Ypourgeio Politismou kai Athlitismou, Tameio Arhaiologikon Poron kai Apallotrioseon, Ergastirio Arhaiologias Panepistimiou Thessalias.

Krystalli-Votsi, K. 1998. The Excavation of the Mycenaean Cemetery at Aidonia. In: Demakopoulou, K. (ed.) The Aidonia Treasure. Seals and Jewelry of the Aegean Late Bronze Age.

Krystalli-Votsi, K. & Kaza-Papageorgiou, K. 2013. To mykinaiko nekrotafeio ton Aidionion (Το μυκηναϊκό νεκροταφείο των Αηδονιών; The mycenaen cemetary at Aidonia). In: Kissas, K. & Niemeier, W.-D. (eds.) The Corinthia and the Northeast Peloponnese. Topography and History from Prehistoric Times until the End of Antiquity.

Kvapil, L. & Shelton, K. 2019. Among the Ancestors at Aidonia. In: Borgna, E., Caloi, I., Carinci, F. M. & Laffineur, R. (eds.) Μνήμη/Mneme: Past and Memory in the Aegean Bronze Age. Proceedings of the 17th International Aegean Conference, University of Udine, Department of Humanities and Cultural Heritage, and the Ca' Foscari University of Venice, Department of Humanities. Peeters Publishers & Booksellers.

Langford-Verstegen, L. 2015. Hagios Charalambos. A Minoan Burial Cave in Crete II, The Pottery, Philadelphia, INSTAP Academic Press.

Lis, B. 2012. Aeginetan pottery in Central Greece and its wider perspective. Arhailogiko Ergo Thessalias kai Stereas Elladas, 3 (2009): Praktika Epistimonikis Synantisis, Volos, 12 – 15 Martiou, Volos.

Mackenzie, D. 1903. The Pottery of Knossos. Journal of Hellenic Studies, 23, 157–205.

Manteli, K. 1992. The Neolithic Well at Kastelli Phournis in Eastern Crete. Annual of the British School at Athens, 87, 103-120.

Maran, J. 2008. Forschungen in der Unterburg von Tiryns 2000–2003. Archäologischer Anzeiger, 35–111.

Maran, J. 2010. Tiryns. In: Cline, E. H. (ed.) The Oxford Handbook of the Bronze Age Aegean (ca. 3000-1000 BC). Oxford University Press.

Maran, J. 2015. Tiryns and the Argolid in Mycenaean Times: New Clues and Interpretations. In: Schallin, A.-L. & Tournavitou, I. (eds.) Mycenaeans Up to Date: The Archaeology of the North-Eastern Peloponnese. Current Concepts and New Directions. Swedish Institute at Athens.

Maran, J. 2016. Against the Currents of History: The Early 12th-Century BCE Resurgence of Tiryns. In: Driessen, J. (ed.) RA-PI-NE-U. Studies on the Mycenaean world offered to Robert Laffineur for his 70th Birthday. UCL Presses Universitaires de Louvain.

Maran, J. & Papadimitriou, A. 2016. Gegen den Strom der Geschichte - Die nördliche Unterstadt von Tiryns: Ein gescheitertes Urbanisierungsprojekt der mykenischen Nachpalastzeit. Archäologischer Anzeiger, 19–118.

McGeorge, P. J. P. 1988. Health and Diet in Minoan Times. In: Jones, R. E. & Catling, H. W. (eds.) New Aspects of Archaeological Science in Greece: Proceedings of a Meeting held at the British School at Athens January 1987 BSA Occ. Paper 3 of the Fitch Laboratory.

McGeorge, P. J. P. 2003. Appendix 2 : Intramural Infant Burials in the Aegean. In: Hallager, E. & Hallager, B. P. (eds.) The Greek-Swedish Excavations at the Agia Aikaterini Square Kastelli, Khania 1970-1987 and 2001. Astrom Editions.

McGeorge, P. J. P. 2011. Trauma, surgery and prehistoric events. Proceedings of the 10th International Congress of Cretan Studies, 1-8 October 2006 Khania. 347-361.

McGeorge, P. J. P. 2011. Intramural infant burials in the Aegean Bronze age: Reflections on symbolism and eschatology with particular reference to Crete. 2èmes Rencontres d'archéologie

de l'IFEA: Le Mort dans la ville Pratiques, contextes et impacts des inhumations intra-muros en Anatolie, du début de l'Age du Bronze à l'époque romaine. IFEA-Ege yayınları.

McGeorge, P. J. P. 2012. The Petras intramural infant jar burial: context, symbolism, eschatology. In: Tsipopoulou, M. (ed.) Petras, Siteia – 25 years of excavations and studies. The Danish Institute at Athens.

McGeorge, P. J. P. 2015. The Earliest Archaeological Evidence for a Mycenaean Greek Ritual Form of Human Sacrifice. Pasiphae, 9, 43-52.

McGeorge, P. J. P. 2017. The Pit L Baby Burial – Hermeneutics: Implications for immigration into Kydonia in MMIII/LMI. Proceedings of the Danish Institute at Athens, 8, 293-302.

McGeorge, P. J. P. 2020. Palaeo-Oncological Findings from Prehistoric Crete. In: Γραμματείας, Κ. Ε. τ. Ε. κ. Λ., ed. Proceedings of the 2nd International Symposium of the European Society for the History of Oncology, 2019 Athens. Athens: Academy of Athens, 81-91.

Morris, M. W. 2002. Soil Science and Archaeology: Three Test Cases from Minoan Crete, Philadelphia, INSTAP Academic Press.

Müller, K. 1975. Das Kuppelgrab von Tiryns. In: Jantzen, U. (ed.) Tiryns: Forschungen und Berichte. Philipp von Zabern.

Mylona, D. 2015. Sacrifices in LM IIIB: Early Kydonia Palatial Centre. The Animal Remains. 9, 53-58.

Mylonas, G. E. 1959. Aghios Kosmas: an Early Bronze Age settlement and cemetery in Attica, Princeton University Press.

Nordquist, G. C. 1987. A Middle Helladic village: Asine in the Argolid. Academia Ubsalaliens.

Pantelidou-Gofa, M. 2005. Tsepi Marathonos: to protoelladiko nekrotafeio [Τσέπι Μαραθώνος: το πρωτοελλαδικό νεκροταφείο; Tsepi at Marathon: the Early Helladic cemetery], Athens, Archaeological Society at Athens.

Pantelidou-Gofa, M. 2008. The EHI deposit pit at Tsepi, Marathon: features, formation and the breakage of finds. In: Brodie, N., Doole, J., Gavalas, G. & Renfrew, C. (eds.) Horizon: a colloquium on the prehistory of the Cyclades. McDonald Institute for Archaeological Research/Stavros Niarchos Foundation.

Pantelidou-Gofa, M. 2016. Tsepi Marathonos: o apothetis 39 tou protoelladikou nekrotafeiou [Τσέπι Μαραθώνος: ο αποθέτης 39 του πρωτοελλαδικού νεκροταφείου; Tsepi at Marathon: the deposit 39 of the Early Helladic cemetery], Athens, Archaeological Society at Athens.

Papadatos, Y. 2008. The Neolithic-Early Bronze Age Transition in Crete: New Evidence from the Settlement at Petras Kephala, Siteia. In: Isaakidou, V. & Tomkins, P. D. (eds.) Escaping the Labyrinth: The Cretan Neolithic in Context. Oxbow Books.

Papadimitriou, A. 2003. Oi ypomykinaikoi kai protogeometrikoi tafoi tis Tirynthas. Analysi kai ermineia [Οι υπομυκηναϊκοί και πρωτογεωμετρικοί τάφοι της Τίρυνθας. Ανάλυση και ερμηνεία]. In: Vlachopoulos, A. & Birtacha, K. (eds.) Argonautis. Timitikos tomos gia ton kathigiti Christo G. Douma apo tous mathites tou sto Panepistimio Athinon (1980-2000) [Αργοναύτης. Τιμητικός τόμος για τον καθηγητή Χρίστο Γ. Ντούμα από τους μαθητές του στο Πανεπιστήμιο Αθηνών (1980–2000)]. Kathimerini.

Papathanasiou, A., Tiliakou, A., Kwok, C. S., Moutafi, I. & Kakavogianni, O. 2020. The human remains of the Late Bronze Age cemetery of Glyka Nera. In: Papadimitriou, N., Wright, J., Fachard, S., Polychronakou-Sgouritsa, N. & Andrikou, E. (eds.) Athens and Attica in Prehistory. Proceedings of the International Conference, Athens, 27-31 May 2015. Archaeopress.

Papazoglou-Manioudaki, L. & Paschalidis, C. 2017. A Society of Merchants and Warriors to the East of the West. The Case of the Mycenaean Settlement on Mygdalia Hill, near Patras, in Achaea. In: Fotiadis, M., Laffineur, R., Lolos, Y. & Vlachopoulos, A. (eds.) 'Εσπερος/Hesperos. The Aegean seen from the West. Peeters.

Papazoglou-Manioudaki, L., Paschalidis, C. & Jones, O. A. 2019. Community and Memory in the Periphery of the Mycenaean World: Incidents in the Life of the Mygdalia Settlement Near Patras, in Achaeae. In: Borgna, E., Caloi, I., Carinci, F. M. & Laffineur, R. (eds.) MNHMH/MNEME: Past and Memory in the Aegean Bronze Age. Proceedings of the 17th International Aegean Conference, University of Udine, Department of Humanities and Cultural Heritage, and the Ca' Foscari University of Venice, Department of Humanities. Peeters.

Persson, P. Å. 2003. A Note on the Foetus. In: Hallager, E. & Hallager, B. P. (eds.) The Greek-Swedish Excavations at the Agia Aikaterini Square Kastelli, Khania 1970-1987 and 2001. Astrom Editions.

Polychronakou-Sgouritsa, N. & Kakavogiannis, E. 2020. Minoans in Mesogaia? In: Papadimitriou, N., Wright, J., Fachard, S., Polychronakou-Sgouritsa, N. & Andrikou, E. (eds.) Athens and Attica in Prehistory Proceedings of the International Conference, Athens, 27-31 May 2015. Archaeopress.

Preve, S. 2009a. "Lendari" excavation (83 A. Papandreou St.). In: Andreadaki-Vlazaki, M. (ed.) Khania (Kydonia). A Tour to Sites of Ancient Memory. Ministry of Culture and Tourism – 25th Ephorate of Prehistoric and Classical Antiquities.

Preve, S. 2009b. "Rovithaki" excavation (11 Malinou St.). In: Andreadaki-Vlazaki, M. (ed.) Khania (Kydonia). A Tour to Sites of Ancient Memory. Ministry of Culture and Tourism – 25th Ephorate of Prehistoric and Classical Antiquities.

Prevedorou, E. 2016. A bioarchaeological perspective on the human skeletal remains from the Mycenaean settlement and cemetery at Lazarides. To Mykinaiko nekrotafeio stous Lazarides Aiginas [Το Μυκηναϊκό νεκροταφείο στους Λαζάρηδες Αίγινας; The Mycenaen cemetery at Lazarides, Aegina].

Protopapadaki, E. 2021. I poli pano sti nekropoli: I nekropoli tis arhaias Kydonias sti xoriki egrafi tis syghronis polis ton Hanion [Η πόλη πάνω στη νεκρόπολη: Η νεκρόπολη της αρχαίας Κυδωνίας στη χωρική εγγραφή της σύγχρονης πόλης των Χανίων]. Masters, Technical University of Crete.

Rudolph, W. 1973. Die Nekropole am Prophitis Elias bei Tiryns. In: Jantzen, U. (ed.) Tiryns: Forschungen und Berichte. Verlag Philipp von Zabern.

Sackett, L. H., Hankey, V., Howell, R. J., Jacobsen, T. W. & Popham, M. R. 1966. Prehistoric Euboea: Contributions toward a survey. Annual of the British School at Athens, 61, 33-112.

Sampson, A. 1980. Proistorikes theseis kai oikismoi stin Euboia [Προϊστορικές θέσεις και οικισμοί στην Εύβοια; Prehistoric sites and settlements in Euboea]. Arheio Euboikon Meleton (Αρχείο Ευβοϊκών Μελετών), 23, 91-249.

Schilardi, D. U. 1999. The Mycenaean horseman (?) of Koukounaries. In: Betancourt, P. P., Karageorghis, V., Laffineur, R. & Niemeier, W.-D. (eds.) Meletemata: Studies Presented to Malcolm H. Wiener as He Enters his 65th Year.

Schilardi, D. U. 2016. Koukounaries, Paros: the excavations and history of a most ancient Aegean acropolis (version in Greek), Athens, Paros excavations, Center of Historical and Archaeological Studies.

Schultz, M. & Schmidt-Schultz, T. 2015. Anthropologie. In: Maran, J. & Papadimitriou, A. (eds.) Tiryns, Griechenland: Die Arbeiten der Jahre 2012 bis 2014.

Serpetsidaki, I. Neoteri anaskafiki ereuna sto neolithiko Katsamba [Νεότερη ανασκαφική έρευνα στο νεολιθικό Κατσαμπά; Latest excavation survey at Neolithic Katsambas]. 11th International Congress of Cretan Studies, 21-27 October 2011 Rethymnon. 2011.

Sgouritsa, N. 2010. Lazarides on Aegina: Another prehistoric site. In: Touchais, G., Philippa-Touchais, A., Voutsaki, S. & J. Wright, J. (eds.) Mesohelladica. The Greek Mainland in the Middle Bronze Age. De Boccard.

Sgouritsa, N. 2012. Remarks on jewels from the Mycenaean Settlement and Cemetery at Lazarides on eastern Aegina. In: Nosch, M.-L. & Laffineur, R. (eds.) Kosmos. Jewellery, Adornment and Textiles in the Aegean Bronze Age. Peeters.

Sgouritsa, N. 2015. Lazarides on eastern Aegina: The relationships with the NE Peloponnese. In: Schallin, A.-L. & Tournavitou , I. (eds.) Mycenaeans Up to Date: The Archaeology of the NE Peloponnese–Current Concepts and New Directions. Proceedings of the Conference held 10-16 November 2010, Swedish Institute at Athens. The Editorial Committee of the Swedish Institutes at Athens and Rome.

Sgouritsa, N. 2021a. Animal-shaped standard-weights in the Aegean of the Mycenaean Era. In: Lambrinoudakis, V., Mendoni, L., Koutsoubou, M., Panagou, T., Sfiroera, A. & Charalampidou, X. (eds.) Έξοχος άλλων. Τιμητικός Τόμος για την Ε. Σημαντώνη –Μπουρνιά.

Sgouritsa, N. 2021b. O Mykinaikos oikismos stous Lazarides Aiginas: deka hronia ereunas. Erotimata pou zitoun akomi apantisi [Ο Μυκηναϊκός οικισμός στους Λαζάρηδες Αίγινας: δέκα χρόνια έρευνας. Ερωτήματα που ζητούν ακόμη απάντηση]. In: Karantzali, E. (ed.) Prakrika tou C' diethnous diepistimonikou symposiou, I Perifereia tou Mykinaikou Kosmou. Prosfata eurimata kai porismata tis ereunas, Lamia 18-21 Maïou [Πρακτικά του Γ΄ διεθνούς διεπιστημονικού συμποσίου, Η Περιφέρεια του Μυκηναϊκού Κόσμου. Πρόσφατα ευρήματα και πορίσματα της έρευνας, Λαμία 18-21 Μαΐου 2018; Proceedings of the third interdisciplinary symposium, The periphery of the Mycenaean Word].

Sgouritsa, N. & Salavoura, E. 2014. The Exploitation of Inland Natural Resources on an Island Environment: The Case of the Mycenaean Settlement at Lazarides and the south/southeast Aegina. In: Touchais, G., Laffineur, R. & Rougemont, F. (eds.) PHYSIS. L'environnement naturel et la relation home-milieu dans le monde égéen protohistorique. Leuven

Theocharis, D. 1959. Ek tis proistorias Euboias kai Skyrou [Εκ της προϊστορίας Ευβοίας και Σκύρου; From the prehistory of Euboea and Skyros]. Arheio Euboikon Meleton (Αρχείο Ευβοϊκών Μελετών), 6, 279-328.

Todaro, S. & Di Tonto, S. 2008. The Neolithic Settlement of Phaistos Revisited: Evidence for Ceremonial Activity on the Eve of the Bronze Age. In: Isaakidou, V. & Tomkins, P. D. (eds.) Escaping the Labyrinth: The Cretan Neolithic in Context. Oxbow Books.

Tomkins, P. D. 2007. Neolithic: Strata IX-VIII, VII-VIB, VIA-V, IV, IIIB, IIIA, IIB, IIA and IC Groups. In: Momigliano, N. (ed.) Knossos Pottery Handbook: Neolithic and Bronze Age (Minoan). The British School at Athens.

Tomkins, P. D. 2009. Domesticity by default. Ritual, ritualization and cave-use in the Neolithic Aegean. Oxford Journal of Archaeology, 28, 125-153.

Tomkins, P. D. 2012. Landscapes of Ritual, Identity, and Memory: Reconsidering Neolithic and Bronze Age Cave Use in Crete, Greece. In: Holley, M. (ed.) Sacred Darkness: A Global Perspective on the Ritual Use of Caves. University Press of Colorado.

Tomkins, P. D. 2018. About Time. Rehabilitating chronology in the interpretation of settlement in east Crete between the Neolithic and Early Minoan I. Creta Antica, 19, 45-92.

Triantaphyllou, S. 2008. Living with the Dead: a Re-Consideration of Mortuary Practices in the Greek Neolithic. In: Isaakidou, V. & Tomkins, P. D. (eds.) Escaping the Labyrinth: The Cretan Neolithic in Context. Oxbow Books.

Trotter, M. 1970. Estimation of stature from intact long limb bones. Personal identification in mass disasters, 71-83.

Tselios, C. 2016. Ergastiriaki Eksetasi ton metallikon eurimaton tou mykinaikou oikismou kai nekrotafeiou ton Lazaridon Aiginas (Εργαστηριακή εξέταση μεταλλικών ευρημάτων του μυκηναϊκού οικισμού και του νεκροταφείου των Λαζάρηδων Αίγινας). To Mykinaiko nekrotafeio stous Lazarides Aiginas [Το Μυκηναϊκό νεκροταφείο στους Λαζάρηδες Αίγινας; The Mycenaean cemetery at Lazarides, Aegina].

Tzedakis, Y. 1970. Anaskafi tou spilaiou Genariou [Ανασκαφή σπηλαίου Γερανίου; Excavation at Gerani Cave]. ArchDelt (Αρχαιολογκόν Δελτίον), 25, 474-476.

Vagnetti, L. 1972-1973. L'insediamento neolitico di Festos. Annuario della Scuola archeologica di Atene e delle Missioni italiane in Oriente, 50-51, 7-138.

Vagnetti, L. 1973. Tracce di due insediamenti neolitici nel territorio dell'antica Gortina, Catania, Università di Catania, Instituto di Archaeologia.

Vassilakis, A. 1987. Anaskafi Neolithikou Spitiou stous Kalous Limenes Kritis [Ανασκαφή Νεολιθικού Σπιτιού στους Καλούς Λιμένες της Νότιας Κρήτης; Excavation of the Neolthic House at Kaloi Limenes, Crete]. In: Platon, N., Kastrinakis, L., Orphanou, G. & Giannadakis, N. (eds.) Eilapini: Tomos timitikos gia ton kathigiti N. Platona (Ειλαπινή: Τόμος Τιμητικός για τον καθηγητή Ν. Πλάτωνα). Δήμος Ηρακλείου.

Warren, P., Jarman, M. R., Jarman, H. N., Shackleton, N. J. & Evans, J. D. 1968. Knossos Neolithic, Part II. Annual of the British School at Athens, 63, 239 – 276.

Wolters, P. 1891. Marmorkopf aus Amorgos. Athenische Mitteilungen, 16, 46-58.

SUPPLEMENTARY NOTE 2

Modeling and dating of genetic admixture

In this section we provide details on allele frequency-based methods that were applied in order to quantify gene-flow into the Aegean populations, infer ancestry mixture models and estimate the dates of the admixture events.

To avoid biases in allele frequencies caused by genetic relatedness, we removed first and second-degree relatives from group-based analyses. For the sites of Mygdalia and Hagios Charalambos, where a high rate of relatedness is observed among the individuals (Extended Data Fig. 4), we also excluded individuals related at the third degree. On the other hand, given the high rate of genotype missingness in Aposelemis individuals, we included both individuals from a second-degree related pair (APO004 and APO028), but we also estimated and compared f_4-statistics and significance levels from APO004 alone (highest SNP coverage).

Individual and group IDs are reported as in https://reich.hms.harvard.edu/allen-ancient-dna-resource-aadr-downloadable-genotypes-present-day-and-ancient-dna-data, and whenever another grouping label was employed, we specify which individual were included.

The following abbreviations are applied on the nomenclature E: Early, M: Middle, L: Late, N: Neolithic, En: Eneolithic, C: Chalcolithic, BA: Bronze Age, W.: Western, S.: Southern, N.: Northern, E.: Eastern, C.: Central, .sg/.SG: pulldown to 1240K SNPs from shotgun sequencing data. We use these abbreviations across the text compositely (e.g., EMBA: Early Middle Bronze Age, or SE: Southeastern).

f_4-statistics

We computed a 4-population test (qpDstat) from ADMIXTOOLS [v57.1] (Patterson et al., 2012) with default parameters and f4mode:YES. qpDstat (or here f_4-statistic) is a formal test of admixture based on the allele-frequency correlation patterns among the four populations of the test. Under a null hypothesis of an unrooted topology of the populations ((A,B),(C,D)), the test f_4(A, B; C, D) calculates $(p_A-p_B)(p_C-p_D)$, which is expected to be zero as the allele differences within these two pairs should not be correlated. The calculation of the summary statistic includes a weighted block jackknife method for the calculation of the standard error (SE). A f_4-statistic that differs from 0 (conventionally beyond ± 3SE or $|Z| > 3$) is interpreted as a rejection of the null topology for a different one, or as gene-flow between A/B and C/D after their divergence. Hence, in order to use the method as a formal test of admixture prior knowledge about the divergence of the four populations is necessary. The sign of the statistic

is also informative about the direction of the allele sharing. A negative statistic means asymmetric allele sharing between A and D or B and C, while an asymmetric allele sharing between A and C or B and D results in a positive value. Another important implication derived from the formula of the 4-population test is that it harnesses information from the internal branches of the topology and therefore, it is insensitive to post gene-flow genetic drift on the branches leading to populations A, B, C and D.

We performed f4-statistics of the form f4(Mbuti, Test; Anatolian farmer group, Aegean groups) (Supplementary Table 4). By placing in the position of 'A' an African population who is a common outgroup to the others, we could directly test for populations who -compared to the Anatolian farmers- share excessive alleles with Neolithic and Bronze Age Aegean groups. As 'Test' we run a battery of populations, earlier or coeval to the Aegean groups, who represent ancestries present in West Eurasia since the Early Holocene. Positive results ($Z \geq 3$) were obtained in the majority for the Late Bronze Age (LBA) Aegean groups and suggest affinity of the latter with populations from East Europe and Central Asia [e.g., Eastern European hunter-gatherers (EEHG)] and West Asia [e.g., Caucasus hunter-gatherers (CHG)], or younger populations related to the pastoralists from the Eurasian Steppe (e.g., Russia_Smara_EBA_Yamnaya, Poland_Globular_Amphora, Italy_Sardinia_LBA, Germany_CordedWare) who in turn derive their ancestry from these earlier groups. Among the preceding Aegean groups (Early and Middle Bronze Age), Nea Styra EBA, Hagios (Hg.) Charalambos EMBA and published Odigitria EMBA from Crete displayed evidence of excessive allele sharing that is specific to earlier/contemporaneous West Eurasian groups from Iran and the Caucasus, and occasionally groups affiliated to the BA Eurasian Steppe. Strongly positive results (i.e., $Z \geq 2.5$ but no more than 4) for the Cretan Neolithic population Aposelemis_N indicate that the groups likely harbors some extra affinity with some Iranian/Levantine populations. Because such affinities are not visible in the PCA, and Aposelemis_N as a group has low heterozygosity (i.e., pairwise mismatch rate is equivalent to second-third degree relatives for the rest of the Aegean dataset - see also Extended Data Fig. 4), we also provide the same f_4 tests but only on APO004, the individual with the highest SNP coverage. By doing so, we can test for overestimation of the significance of the allele frequency differences between Aposelemis and W.Anatolia_N/ Mainland_Greece_N, owing to long-term inbreeding at Aposelemis. We note that the most positive Z scores decreased (now <2, with the exception of Iran_C_TepeHissar, ANE and Levant_C with $3<Z\leq2$), while for some test the sign became negative or more negative (i.e., WEHG, BalkanHG) making the tests more consistent with those from f_4(Mbuti, Test; W.Anatolia_N, Mainland_Greece_N).

Admixture f_3-test

We performed a test of admixture f_3(A,B;C) from ADMIXTOOLS [v57.1] (Patterson et al., 2012) which essentially tests whether allele frequencies in the target population C are intermediate between those of the source populations A and B, in which case the statistic becomes negative. However, high rate of data missingness or post-admixture drift on the branch of 'C' can hinder the power of the test to detect admixture. To enhance the statistical power of the test, we used merged meta-populations. For population A, we used Early European-Anatolian farmers (EEAF) that include Anatolia_N (Barcın, Menteşe and Bocuklu), Germany_EN_LBK (associated with the Early Neolithic Linear Pottery archeological horizon in Central Europe). For population B, we iterated over Anatolia_LC-EBA (sites Arslantepe, İkiztepe, Çamlıbel Tarlası, Harmanören-Göndürle Höyük), mCaucasus_En-BA (mountain Caucasus Eneolithic-Bronze Age: I1635, I1633, I1658, I1656, RISE396.SG, RISE397.SG, RISE407.SG, RISE408.SG, RISE412.SG, RISE413.SG, RISE416.SG, RISE423.SG, DA31.SG, DA35.SG, ARM001, ARM002, I1720, I2051, I2056, I6266, I6267, I6268, I6272, KDC001, KDC002, MK5004, MK5008, OSS001, SA6002, VEK007, and the lowland ALX002), W. Eurasian Steppe En-BA ('WES' ancestry, i.e., Yamnaya pastoralists from the Caucasus, Eastern Russia and Ukraine: I0370, I0441, I0444, I0439, I0357, I0429, I0438_published, RISE240.SG, RISE546.SG, RISE547.SG, RISE548.SG, RISE550.SG, RISE552.SG, I0443, I2105, I3141_published, I7489, BU2001, GW1001, I1723, KBD001, LYG001, MK3003, MK5009, PG2001, PG2002, PG2004, RK1001, RK1003, RK1007, RK4001, RK4002, SA6003, VJ1001, ZO2002, I0231_published), CHG (Caucasus hunter-gatherers: Kottias KK1.SG and Satsurblia SATP.SG), Eastern European hunter-gatherers (EEHG: I0124, I0211, I0061, Popovo2, UzOO77_new), and W. Iran N (Iran_GanjDareh_N). We chose these groups according to the f_4-statistics and their PCA coordinates (Fig. 2). We run the admixture f_3-test with the option inbreed:YES, which is recommended by the authors of the method when the target population is pseudo-diploid data. We present evidence of admixture detected with the test (< -3 SE) (Supplementary Table 5). Among the M/LBA groups, Chania LBA is the largest group (n=27) and exhibits the strongest signals of admixture between a population like EEAF and a population related to East Europe, the Caucasus and Iran, but not with Anatolia LC-EBA. Aidonia LBA, Glyka Nera LBA, Krousonas LBA, Logkas MBA, Mygdalia LBA and Tiryns LBA also display admixture signals from one or more distal sources (CHG, EEHG or W. Iran N), but always from W. Eurasian Steppe En-BA. On the contrary, for the earlier Nea Styra EBA admixture source include the proximal mount. Caucasus En-BA,

and -more weakly- Anatolia LC-EBA (Z = 1.14). Notably, no admixture evidence is obtained for Hg. Charalambos EMBA or Odigitria EMBA from Crete despite the evidence from PCA and f_4-statistics, which could be attributed to private drift on the lineage leading to these groups.

Dating of recent admixture

Using the same setting of source and target populations, we tested for a signal of recent admixture with DATES (https://github.com/priyamoorjani/ DATES) (Methods). The method effectively estimates local ancestry within the target individual/population -that is, whether a genomic segment descends from either of the two sources. The size of these segments is expected to decay across genetic distance at a rate that depends on the time since admixture.

To minimize standard errors for the admixture dates, we compiled five Late Neolithic-Bronze Age supergroups as targets based on geography and archeological periodization: 1. Crete LBA [Chania, Krousonas, Aposelemis and published Armenoi (Lazaridis et al., 2017)], 2. Crete EMBA [Hagios Charalambos from this study, Hagios Charalambos and (Moni) Odigitria previously published (Lazaridis et al., 2017), and Kephala (Clemente et al., 2021)], 3. S. Mainland-Islands LN-EBA [LN I3708, I3709, I2318 and I3920 from Peloponnese (Mathieson et al., 2018), Kou01.SG , Kou03.SG Koufonisia and Euboea (Clemente et al., 2021), and Nea Styra and Lazarides], 4. S. Mainland-Islands LBA [Aidonia, Glyka Nera, Mygdalia, Tiryns, Lazarides, Koukounaries and previously published Apatheia, Pylos and Salamis (Lazaridis et al., 2017)] and 5. N. Mainland MBA [two Logkas individuals (Clemente et al., 2021)]. In addition, we dated admixture separately on Chania LBA and Nea Styra EBA to test whether the variation in the PCA is consistent with more recent admixture. We also merged W. Eurasian Steppe En-BA with ‘Germany_CordedWare’ into the supergroup source 2 ‘Steppe-Corded Ware En-BA’. We present the results and mark the tests for which a recent date was inferred and an exponential decay curve against the genetic distance was fitted (Supplementary Table 6). We also present the admixture dates in BC format. On average, earlier groups (i.e., S. Mainland-Islands LN-EBA and Crete EMBA) are consistent with older admixture dates (ca. 5000 BC), while the succeeding LBA groups with earlier ones (ca. 3000 BC). Admixture on the group Nea Styra is younger than the average on all LN-EBA individuals combined, but for Chania LBA the admixture date matches the average from the group Crete LBA. In addition, for the two supergroups S. Mainland-Islands LBA and N. Mainland MBA, the more recent admixture is inferred only with either EEHG or Steppe-CordedWare En-BA as sources. On the contrary, more genetically distinct sources produce similar decay and date estimation for the earlier groups, which might point to a reduced resolution of the method to

distinguish between these sources for older admixture events. Furthermore, these results corroborate a multi-phased genetic admixture in the Aegean between the 6th and 2nd millennia BC, also shown with the *f*-statistics and the *qpAdm* modeling (see below). We acknowledge though that contextualization of the aforementioned dates with archeological evidence comes with certain limitations as the method cannot distinguish between one-pulse and continuous admixture. In particular, this hinders robust signals of very recent admixture in Crete_LBA as the incoming source(s) were also most likely very recently admixed.

Ancestry deconvolution with *qpWave/qpAdm*

To better understand the interplay of the various ancestry components that formed the gene-pool of the Aegean populations since the Neolithic until the end of the Bronze Age, we performed analyses with the *qpWave/qpAdm* tools from ADMIXTOOLS [v7.1] (Patterson et al., 2012) (see also Methods). A critical parameter when using these tools is the choice of reference (outgroup or 'right') populations. While explicit knowledge of the phylogeny relating those populations is not necessary, the method behind these tools relies on some assumptions in order to infer meaningful models. For *qpWave*, which is usually used to estimate the minimum number of independent gene pools that explain a set of targets from the references, it is important that the references are chosen such that the target populations are differentially related to them because of their deeper or more recent evolutionary history. For *qpAdm*, which is used to fit ancestries on a target from a set of source populations and estimate mixture coefficients, two more criteria need to be fulfilled. First, the references should be related with the target through the source populations and second, there should be no subsequent direct gene flow between the references and the target.

We first run *qpWave* under default parameters in order to cluster individuals within and/or across sites that are genetically indistinguishable compared to the set of reference (right) populations. We set the following set of eleven ancient reference populations (R11) from published studies (Methods): Ethiopia_4500BP_published.SG (Mota.SG), Russia_Ust_Ishim_HG_published.SG, Russia_Kostenki_14, Balkan_HG (hunter-gatherers from the Iron Gates in Serbia), Western European hunter gatherers (WEHG; Loschbour_published.DG, Iboussieres25-1, Iboussieres31-2, Rochedane, BerryAuBac, I1507, Villabruna, I2158, Bichon.SG, I1875, I4971, Falkenstein, Chaudardes1_published, Ranchot88_published), CHG, EEHG, Israel_Natufian_published, Ancestral North Eurasians (ANE; MA1.SG and AfontovoGora2.SG), 'W. Iran N' (Iran_GanjDareh_N) and 'W. Anatolia N' (individuals from sites Barcın and Menteşe in the Marmara Sea). With the exception of

Mota.SG who is an outgroup, the remaining populations represent Upper Pleistocene and Early Holocene lineages present in West Eurasia and beyond.

We summarise the results from the pairwise *qpWave* models in the form of a heatmap (Extended Data Fig. 1). Grey tiles correspond to rejected models (p-values < 0.01), which suggests that more than one stream of ancestry from the references are necessary to explain the given pair of individuals. Subsequently, grouping such individuals for downstream analyses (i.e., *qpAdm*) can underestimate genetic complexity. The highest rate of p-values < 0.01 is observed for pairs including an individual from the Late Bronze Age and an individual from a previous period (Neolithic or Early-Middle Bronze Age) (upper left or lower right corner of heatmap). Within sites of the same period (black-outlined squares), individuals are clustering together and the rate of rejected models (e.g., individuals HGC*XXX*, I071, I073, I074 and I9005 from Hagios Charalambos) is the expected for true models to be rejected with a cut-off of 1% given a uniform distribution of the p-values. However, a high rate of rejected models is observed among individuals from Chania LBA (XAN*XXX*), and the islands of Euboea, Koufonisia and Aegina [Nea Styra EBA (NST*XXX*), Mik15, Kou001/3, and Lazarides (LAZ0017)].

We repeated the same analysis on the MBA and LBA individuals adding 'W. Eurasian Steppe En-BA' to R11 (Extended Data Fig. 2.A). With this setting we wanted to test whether this metapopulation can pull additional differences among the LBA individuals, implying that their affinity to WES-related ancestry shown with f_4-statistics was diverse within the Greek mainland, Crete and the other islands. Within sites of the same period, the new set of references increased the number of non-cladal pairs in Chania from 56 to 65 (p-value ≥ 0.01), or from 14 to 27 (p-value ≥ 0.05). Overall though, the *qpWave* analysis under the two setting supported the 'Site_period' grouping with the exception of the published individuals from Logkas and Koufonisia, as well as Nea Styra and Chania.

We performed *qpAdm* modeling both per individual, as well as on groups. Besides the 'Site_period' groups, we split Chania in 'Chania LBA (a)', 'Chania LBA (b)' and 'Chania (XAN030)' and , and grouped Hg. Charalambos, Odigitria and Kephala from Early/Middle Bronze Age (Minoan) Crete as 'Crete EMBA'. By applying this mixed approach of grouping, we reconcile the need to maintain geographical and chronological designations for testing of archeological hypotheses with the need to reduce redundancy on a genetic level, and thereby increase the resolution of admixture inferences based of allele-frequencies (i.e., *qpAdm*) (Eisenmann et al., 2018). Accordingly, we also tested *qpAdm* models with proximal sources on the group of all LN-EBA individuals from Euboea, other islands as well as Peloponnese ('S.

Mainland-Islands LNEBA'). Despite the differences among these individuals indicated by *qpWave* and the per-individual *qpAdm* models, we tested whether this group was on average consistent with admixture from some proximal sources rather than others, a difference that might not be captured when the targets are single individuals with low SNP coverage.

'Crete N' (Aposelemis): One-way qpAdm models from Anatolia

We carried out *qpAdm* analysis (default parameters) on 'Crete Aposelemis N' initially by testing the scenario that the group is a sister clade of Neolithic Anatolian groups of farmers. As a set of reference populations, we applied R11b: Ethiopia_4500BP_published.SG, Russia_Ust_Ishim_HG_published.DG, Russia_Kostenki14, BalkanHG, EEHG, ANE, Israel_Natufian_published, WEHG, CHG, W. Iran N and 'S. Levant N'. 'S. Levant N' which consists of ten Pre-Pottery Neolithic individuals from Israel and Jordan. The two 7th millennium BC ceramic farming groups from Çatalhöyük in Central Anatolia and the Marmara Sea region ('W. Anatolia N') could model 'Crete Aposelemis N' (p-value of one-way model = 0.2 and 0.58, respectively). On the contrary, Aposelemis was not cladal to either the Central Anatolian aceramic farmers from Boncuklu (9th millennium BC), or the 7th millennium BC Tepecik Ciftlik -located more to the east and harboring additional ancestry related to W. Iran N (p-value = $9.3*10^{-5}$ and $2.5*10^{-3}$, respectively). This stark difference between the fit of the ceramic and aceramic farmers for Aposelemis was also noted for many of the other Neolithic individuals from the Greek mainland (e.g., Rev05, I2937, I2318, I3708), excluding those later who were shown to require additional contribution from a CHG/W. Iran N-related source (e.g., I3920) (Fig. 3).

We also checked whether the inclusion of the Boncuklu group in the references [R11b + Turkey_Boncuklu_N(.SG)] influenced the fit of the one-way model from W. Anatolia N. We found that although the p-value decreased, the model for Aposelemis N as cladal to W. Anatolia N remained adequate (p-value = 0.08). We also checked the one-way model from the three earliest Aegean Neolithic individuals in the Greek mainland 'Mainland_Greece_N' (Rev5.SG, I5427 and I2937), which was also adequate (p-value=0.8). Overall, these analyses support a shared genetic pool within the Aegean as far as the inner part of the Anatolian littoral, without further contribution from populations related to the Levant and/or Iran.

Ancestry modelling for the Early/ Middle Bronze Age Aegean populations

We implement a framework for *qpAdm* in which we explore two-way admixture models by fixing the first source to be either W. Anatolia N or Crete Aposelemis N. The plausible

populations serving as second sources were chosen based on insights provided by the PCA and f_4-statistics. We assess the fit of the models by rotating the second source, that is by moving the remaining candidate sources 2 in the reference (right) populations. This ‘competing’ approach was shown to enable *qpAdm* to differentiate between genetically similar sources that would otherwise result in equally fitting models if tested independently (Harney et al., 2021). For the Late Neolithic and Early/Middle Bronze Age groups (‘Crete EMBA’, ‘S. Mainland-Islands LN-EBA’), we rotated among the distal CHG, EEHG, W. Iran N, S. Levant N, and proximal Iran C (from She Gabi), Anatolia LC-EBA, (mountain) Caucasus En-BA and W. Eurasian Steppe En-BA. These temporally proximal metapopulations can be modelled as a linear combination from the temporally distal sources. The fixed references included Ethiopia_4500BP_published.SG, Russia_Ust_Ishim_HG_published.DG, Russia_Kostenki14, BalkanHG, ANE, Turkey_Boncuklu_N, Israel_Natufian_published, WEHG (R8) (Supplementary Table 7). Models with ‘Crete Aposelemis N’ as source 1 became adequate for the majority of the rotating sources 2. On the contrary, models with ‘W. Anatolia N’ as source 1 were estimated on ca. twice as many SNPs and were doomed inadequate besides source 2 being mount. Caucasus En-BA for S. Mainland-Islands LN-EBA. For Crete EMBA the less strongly rejected model included Anatolia LC-EBA (p-value = $1.5*10^{-3}$), which lead us to explore possible tree-way admixture models with W. Anatolia N and Anatolia LC-EBA as fixed sources. By rotation of the remaining candidate sources, only the three-way W. Anatolia N’ + ‘Anatolia LC-EBA’ + (5.4 + 2.9%) ‘W. Iran N’ became adequate, but notably, it did not serve as an alternative model for ‘S. Mainland LN-EBA’ (p-value = $8.91*10^{-3}$). Only when this model was substituted with ca. 10% from CHG it fitted the data from S. Mainland LN-EBA (p-value = 0.48). These results suggest that in the central Aegean (e.g., Euboea, Cyclades, Peloponnese) gene-flow could have been directly associated with populations from the Caucasus (the mountain area and to the south), whereas in Crete with Anatolia, precisely to populations likely lying on the W/C. Anatolian-Caucasian genetic cline.

Ancestry modelling for the Middle/Late Bronze Age Aegean populations

Multiple analyses (f_4-statistics, admixture f_3, PCA and DATES) point to a substantial change in the ancestry profile of the Aegean populations towards the Late Bronze Age. In fact, most all the LBA groups including those from Crete exhibit affinities with ancient Eastern European populations that are not observed in the preceding populations. Archeological evidence suggests that movements of people from Northern Greece and the Balkans (currently underrepresented in the aDNA record) towards southern parts of the Greek mainland were a

regular feature that started already in the 4th millennium BC (Maran, 1998). Therefore, gene-flow from northern populations could be taking place at the time of the EBA individuals of this study. However, confined to Euboea -an island adjacent to the west with the central Greek mainland, the EBA individuals of this study might not serve as a surrogate of the biological relations between the mainland and these northern populations.

We first modelled the genetic shift from EBA/EMBA to M/LBA by trying exploratively two-way models for LBA groups from the corresponding local source ('Crete EMBA', 'S. Mainland-Islands_LN-EBA', or 'N. Mainland LN' consisting of individuals Klei10 and Pal07), and selected representative sources from a set of Late Neolithic-Bronze Age European populations from published datasets (Supplementary Table 8). We applied R11. We also tested one-way models from these local sources as well as non-local but genetically similar groups like 'Italy-Sicily LBA', 'Croatia MBA', and 'Italy BA (WES)'. The latter group consists of individuals from the N. and C. Italy (Regina Margherita and Broion) that represent the earliest presence of WES-related ancestry in the peninsula (Saupe et al., 2021). Some groups showed continuity from their local baseline (i.e., 'Aposelemis LBA', 'Chania LBA (a)' and 'Salamis LBA'), but the remaining 'Site/Individual_MBA/LBA' required additional contribution from one of these external sources: Serbia EBA (Mokrin Necropolis - Maros culture), W. Eurasian Steppe En-BA, Czech EBA 'Bell Beaker', Czech Bohemia EBA 'Unetice', Croatia MBA or Italy BA (WES). Individuals XAN030, Pylos LBA, Logkas2 MBA, Logkas4 MBA and the small groups (n=2) Glyka Nera LBA and Apatheia LBA could also be modelled as cladal from Croatia MBA or Italy BA (WES). To overcome model discrepancies owing to variable group sizes and SNP coverage among the target groups, we grouped them in six supergroups based on geography and their overlapping coefficients of WES-related source (Fig. 4A): 'Mainland LBA' (southern), 'Mainland MBA' (northern), 'Islands LBA', and 'Crete LBA' altogether, or split in 'Crete LBA (Group A)', 'Crete LBA (Group B)' and 'Crete LBA (Group C)'. We tested whether some of the candidate sources fit better in the models than others by rotating them: Germany LN-EBA 'Corded Ware' (C. Europe), W. Eurasian Steppe En-BA (E. Europe), Serbia EBA and Croatia MBA (SE. Europe), and Italy BA (WES) (W. Europe). Notably, models with the most spatially proximal sources like Serbia EBA, Croatia MBA and Italy BA (WES) failed for the Islands and the Mainland (p-value $\leq 2.3*10^{-3}$). On the contrary, two-way models with any of these sources were adequate for all Crete LBA or the two groups (B and C) separately (Fig. 4B, Supplementary Table 9). Alternatively, Group C could be modelled as cladal to (S.) Mainland LBA, which is counterintuitive to the WES ancestry (modeled with Germany LN-EBA 'Corded Ware') being significantly higher in Chania Group C (i.e., 32 ± 2.7 %; 1SE) than

in (S.) Mainland LBA (i.e., 24 ± 1 %; 1SE). As this inconsistency could reflect a lack of resolution of *qpAdm* to distinguish between these two genetically very similar groups, we do not interpreter this result as direct evidence of immigrants from the S. Mainland in Chania, but equally consider the broader Aegean region as well as admixture from less proximal sources similar to Italy BA (WES).

References

Clemente, F., Unterländer, M., Dolgova, O., Amorim, C. E. G., Coroado-Santos, F., Neuenschwander, S., Ganiatsou, E., Cruz Dávalos, D. I., Anchieri, L., Michaud, F., et al. 2021. The genomic history of the Aegean palatial civilizations. *Cell.*

Eisenmann, S., Bánffy, E., van Dommelen, P., Hofmann, K. P., Maran, J., Lazaridis, I., Mittnik, A., McCormick, M., Krause, J., Reich, D., et al. 2018. Reconciling material cultures in archaeology with genetic data: The nomenclature of clusters emerging from archaeogenomic analysis. *Scientific reports,* 8**,** 13003-13003.

Harney, É., Patterson, N., Reich, D. & Wakeley, J. 2021. Assessing the performance of qpAdm: a statistical tool for studying population admixture. *Genetics*.

Lazaridis, I., Mittnik, A., Patterson, N., Mallick, S., Rohland, N., Pfrengle, S., Furtwangler, A., Peltzer, A., Posth, C., Vasilakis, A., et al. 2017. Genetic origins of the Minoans and Mycenaeans. *Nature,* 548**,** 214-218.

Maran, J. 1998. *Kulturwandel auf dem griechischen Festland und den Kykladen im späten 3. Jahrtausend v. Chr. Studien zu den kulturellen Verhältnissen in Südosteuropa und dem zentralen sowie östlichen Mittelmeerraum i. d. späten Kupfer- und frühen Bronzezeit,* Bonn, Habelt.

Mathieson, I., Alpaslan-Roodenberg, S., Posth, C., Szécsényi-Nagy, A., Rohland, N., Mallick, S., Olalde, I., Broomandkhoshbacht, N., Candilio, F., Cheronet, O., et al. 2018. The genomic history of southeastern Europe. *Nature,* 555**,** 197-203.

Patterson, N., Moorjani, P., Luo, Y., Mallick, S., Rohland, N., Zhan, Y., Genschoreck, T., Webster, T. & Reich, D. 2012. Ancient Admixture in Human History. *Genetics,* 192**,** 1065.

Saupe, T., Montinaro, F., Scaggion, C., Carrara, N., Kivisild, T., D'Atanasio, E., Hui, R., Solnik, A., Lebrasseur, O., Larson, G., et al. 2021. Ancient genomes reveal structural shifts after the arrival of Steppe-related ancestry in the Italian Peninsula. *Current Biology*.

SUPPLEMENTARY NOTE 3

Genotype imputation and pedigree reconstruction for Mygdalia individuals

In this section we describe additional analyses to resolve the pedigree among the three infants from the burial at Mygdalia.

The pair of infant males MYG001 and MYG008 are inferred to be full siblings, and both of them are inferred to be second degree-related with the infant male MYG006 (using READ and *lcMLkin*, see Methods; Fig. 5, Extended Data Fig. 4). The full siblings MYG001 and MYG008 have the same mtDNA and Y-chr haplogroup. Their second-degree relative MYG006 has the same Y-chr haplogroup as well, but a different *mt*DNA haplogroup. In addition, all three individuals are related to three more infant individuals at Mygdalia (see Fig. 5; e.g., all three are second degree relatives of MYG002). These lines of evidence limit the possible pedigrees to two: 1. MYG001/MYG008 are double first cousins with MYG006, so they share all four grandparents, or 2. MYG001/MYG008 are half-siblings with MYG006 sharing the same father (same Y-chr but different *mt*DNA haplogroup).

To distinguish between these two scenarios, we use the fact that scenarios 1 and 2 differ in *k2*, the probability that a pair of individuals shares both alleles at a locus as identical by descent (IBD), which is expected to be 0.0625 and 0, respectively. While *lcMLkin* estimates *k0, k1* and *k2* and can successfully distinguish between parent-offspring and full siblings, however *k2* estimates can become too noisy for more distantly related pairs, especially when coverage on 1240K positions is low (<1x).

Therefore, we decided to directly assess identical diploid genotypes along the genome. For this analysis, we computed genotype imputations using a reference panel as described below. The pair of full siblings MYG001 and MYG008 have long stretches of identical diploid markers on several chromosomes, as expected for a pair of full siblings (Supplementary Figure 36). However, both pairs MYG001-MYG008 and MYG001-MYG006 lack any such stretches of fully identical genotypes (Supplementary Figure 36), which would be expected for double first cousins (in ca. 6.25% of the genome), thereby pointing toward both pairs being paternal half-siblings.

For the genotype imputations, we used the 2bp-masked *q30*-filtered reads of the three Mygdalia individuals' ds-libraries and called genotype likelihoods (GL) through GATK [v3.5]

(DePristo et al., 2011). We called GL for all 29,083,171 diallelic positions with a minor allele count 5 or higher contained in the 1000 Genomes Phase 3 release (Auton et al., 2015), using the "UnifiedGenotyper" module with a mean base quality score higher than 30 "-mbq 30". We then provided these likelihoods to GeneImp [v1.3] software (Spiliopoulou et al., 2017) for the statistical imputation of all the 1KG SNPs, using all the 2,504 individuals of the 1KG statistically phased genomes as reference dataset. The imputation was run independently using three different window lengths "kl" {15, 20, 25}, following the developers' instructions (Spiliopoulou et al., 2017). For each imputed SNP the average genotype call probability (GP) across the three different runs was considered. GeneImp also assigns a posterior probability (PP) for each diploid call. For the evaluation of the performance of the imputation, we considered only the SNPs overlapping with the 1240K panel by extracting these specific genotypes and then merged them with the HO dataset (Methods) by keeping the intersection of the two panels. We computed PCA with smartpca in EIGENSOFT [v6.01] package (Patterson et al., 2006; Price et al., 2006) and projected the imputed data for four PP thresholds (0.9, 0.95, 0.99, 0.999) (Supplementary Figure 37A). All imputed versions were shifted in PC1 and PC2 towards modern European populations who are included in the reference panel, but this deviation from the original pseudo-diploid counterparts is very subtle. To further quantify the performance of imputations and the possible introduction of reference bias, we calculated heterozygosity from the imputed data applying the stringiest PP cut-off (0.999) (Supplementary Figure 37B) and computed outgroup f_3-statistics (Supplementary Figure 38). For the individuals with high initial coverage (MYG006 and MYG008, ca. 7-8x coverage), the proportions of heterozygous calls matched those calculated with pmr among non-related pairs from Mygdalia and other BA sites (ca. 0.26). For the lower-coverage individual MYG001 (3x) the heterozygosity was slightly lower (ca. 0.25). No reference bias could be detected with f_3-*statistics* (Supplementary Figure 38).

Supplementary Figure 36. Opposing genotypes along the 22 autosomes for the three pairs of infants from Mydgalia. For each of the three possible pairs between MYG001, MYG006 and MYG008, a subfigure shows identical and differing diploid imputed genotype status along the autosomes (raised and lowered points, respectively). The figures are restricted to SNPs from the 1240K panel with reported probability of the imputed diploid genotype at least 0.999. The pair of full siblings MYG001 and MYG008 have long stretches of identical diploid markers on several chromosomes. In contrast, the two other pairs of individuals do not have such stretches. But such identical stretches would be expected for double first cousins (in expectation 6.25% of the genome).

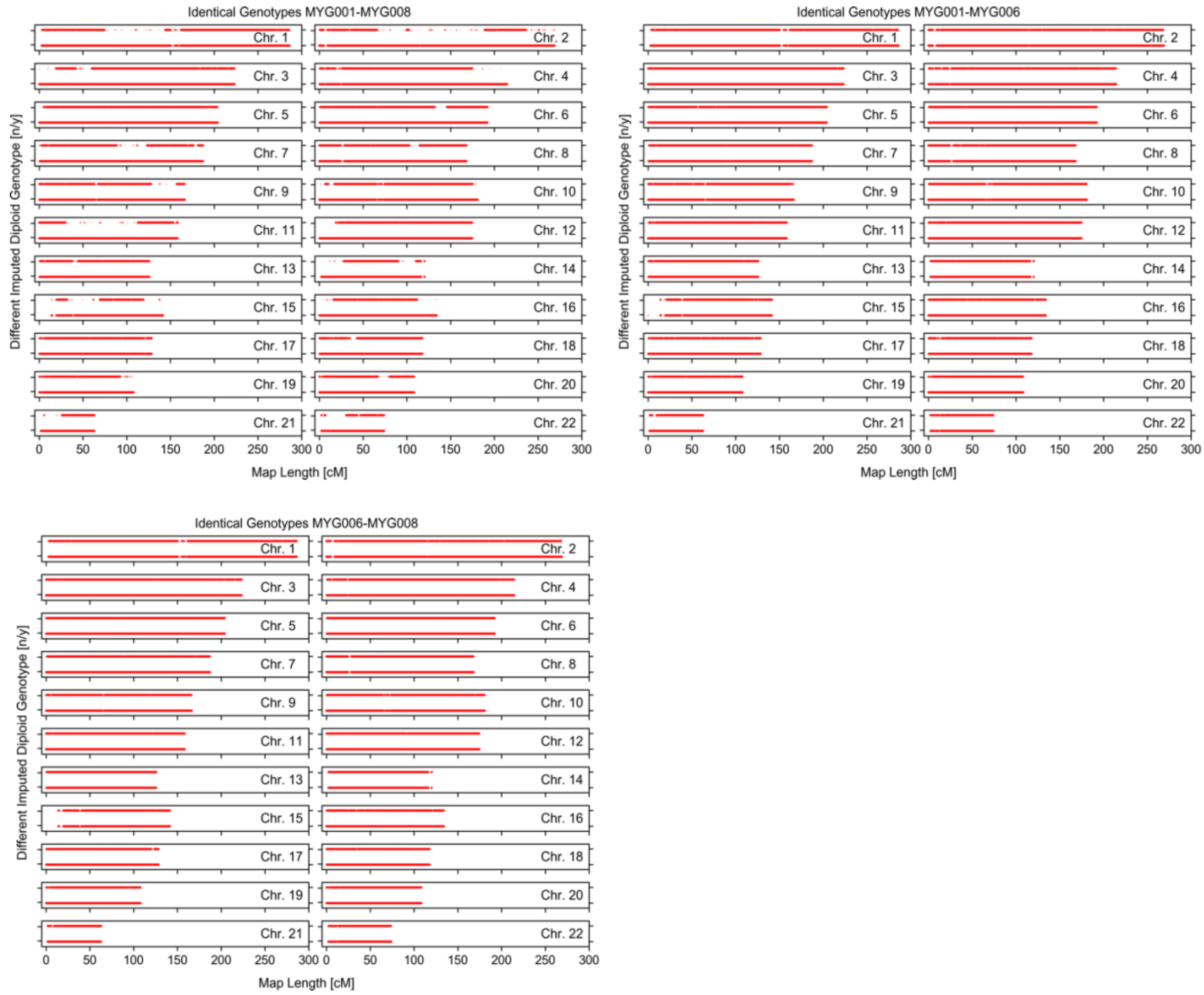

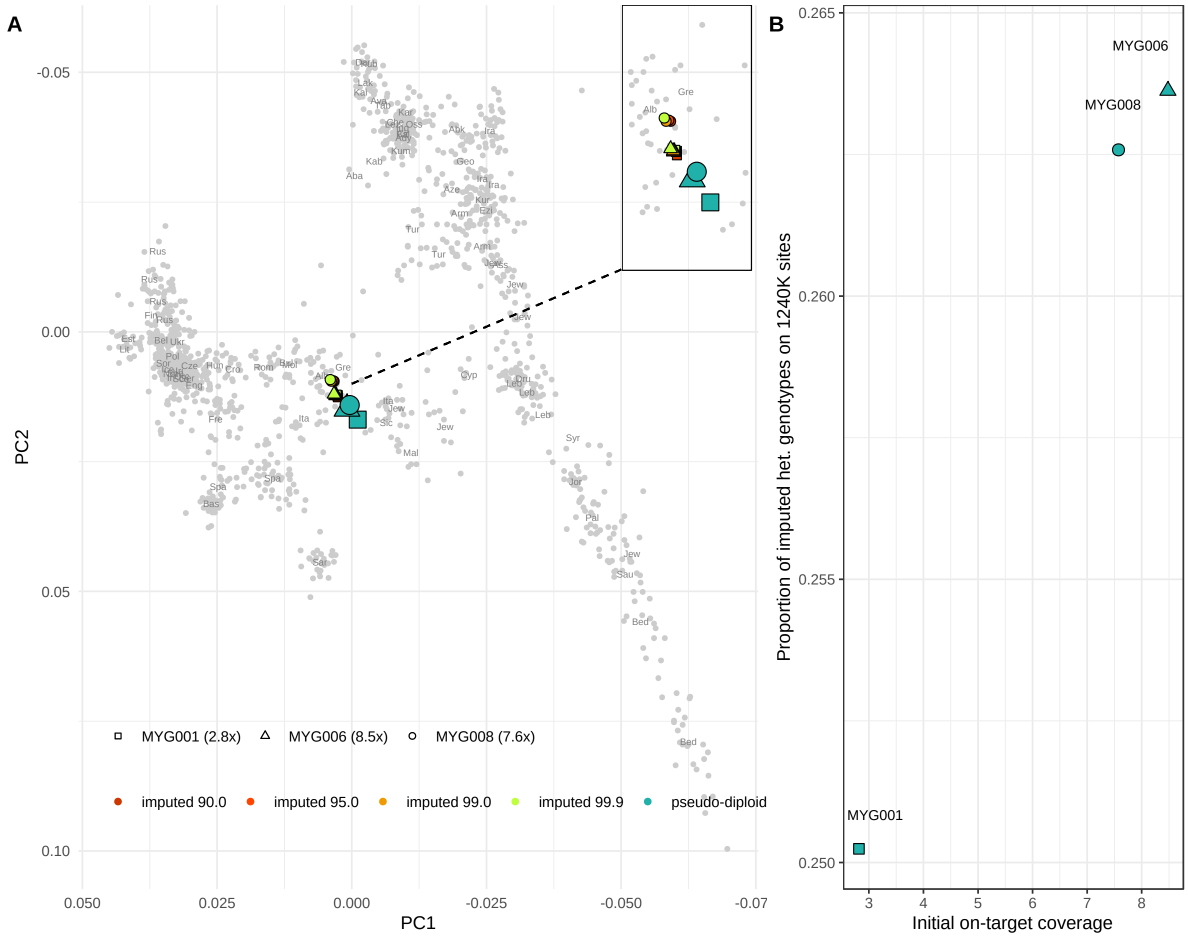

Supplementary Figure 37. A. West-Eurasian PCA with modern individuals plotted as individuals and as mean of within-population PC1 and PC2 coordinates. The pseudo-diploid and imputed genotypes for different PP thresholds (in %) of the three Mygdalia individuals are projected.

B. Heterozygosity calculated as the proportion of imputed (0.99 PP) heterozygous over all imputed genotypes against initial coverage on 1240K positions.

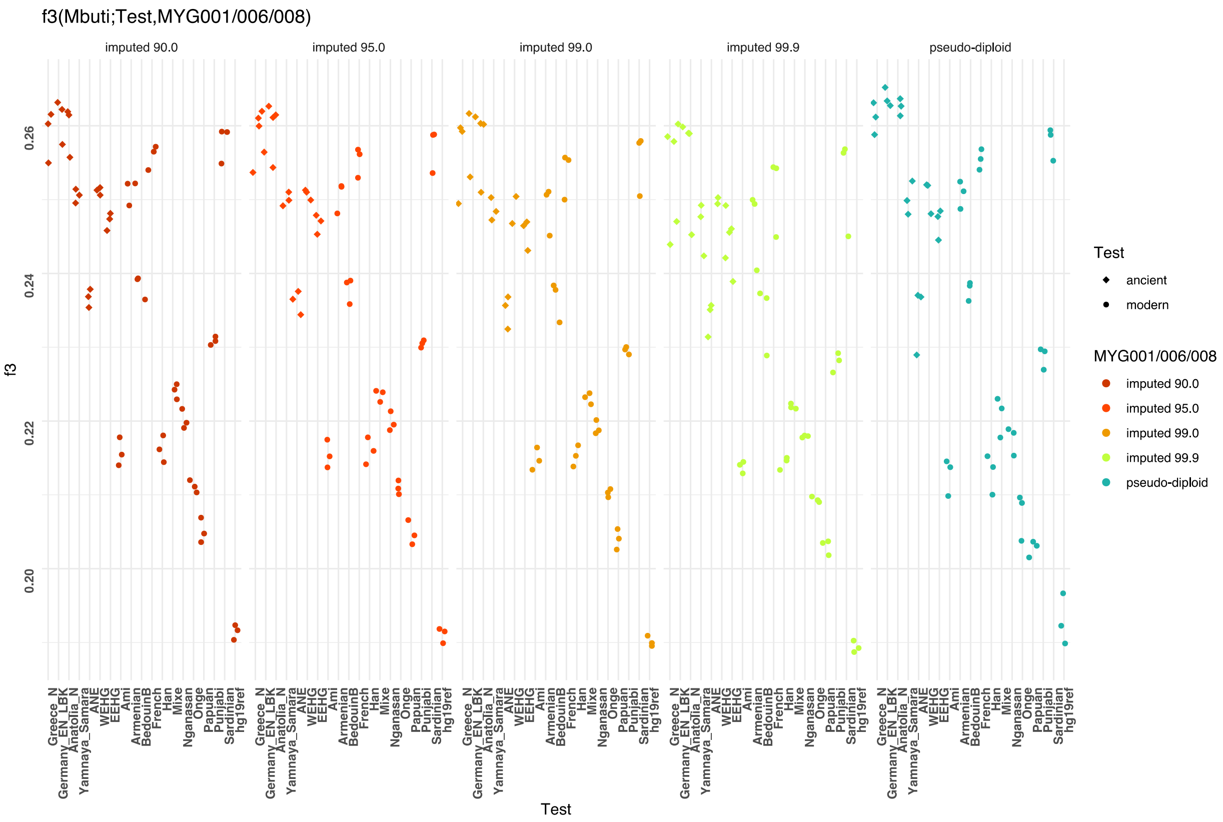

Supplementary Figure 38. Genetic drift measures with *f*$_3$*(Mubti;Test, MYG001/006/008)* with Test being non-African modern populations and some representative ancient groups.

References

Auton, A., Abecasis, G. R., Altshuler, D. M., Durbin, R. M., Abecasis, G. R., Bentley, D. R., Chakravarti, A., Clark, A. G., Donnelly, P., Eichler, E. E., et al. 2015. A global reference for human genetic variation. *Nature*, 526**,** 68-74.

DePristo, M. A., Banks, E., Poplin, R., Garimella, K. V., Maguire, J. R., Hartl, C., Philippakis, A. A., del Angel, G., Rivas, M. A., Hanna, M., et al. 2011. A framework for variation discovery and genotyping using next-generation DNA sequencing data. *Nature Genetics*, 43**,** 491-498.

Patterson, N., Price, A. L. & Reich, D. 2006. Population Structure and Eigenanalysis. *PLOS Genetics*, 2**,** e190.

Price, A. L., Patterson, N. J., Plenge, R. M., Weinblatt, M. E., Shadick, N. A. & Reich, D. 2006. Principal components analysis corrects for stratification in genome-wide association studies. *Nature Genetics*, 38**,** 904-909.

Spiliopoulou, A., Colombo, M., Orchard, P., Agakov, F. & McKeigue, P. 2017. GeneImp: Fast Imputation to Large Reference Panels Using Genotype Likelihoods from Ultralow Coverage Sequencing. *Genetics*, 206**,** 91-104.
