# Supplementary material for: Ancient DNA reveals admixture history and endogamy in the prehistoric Aegean
Source: Nat Ecol Evol. 2023 Jan 16;7(2):290–303. doi: 10.1038/s41559-022-01952-3 (PMC9911347; doi:10.1038/s41559-022-01952-3)
Supplement: Supplementary file 2 — Reporting Summary [file 41559_2022_1952_MOESM2_ESM.pdf]

nature portfolio

Corresponding author(s): Eirini Skourtanioti, Philipp Stockhammer, Choongwon Jeong, Johannes Krause

Last updated by author(s): Sep 22, 2022

Reporting Summary

Nature Portfolio wishes to improve the reproducibility of the work that we publish. This form provides structure for consistency and transparency in reporting. For further information on Nature Portfolio policies, see our Editorial Policies and the Editorial Policy Checklist.

Statistics

For all statistical analyses, confirm that the following items are present in the figure legend, table legend, main text, or Methods section.

n/a	Confirmed	
☐	☒	The exact sample size (*n*) for each experimental group/condition, given as a discrete number and unit of measurement
☐	☒	A statement on whether measurements were taken from distinct samples or whether the same sample was measured repeatedly
☐	☒	The statistical test(s) used AND whether they are one- or two-sided<br>*Only common tests should be described solely by name; describe more complex techniques in the Methods section.*
☐	☒	A description of all covariates tested
☐	☒	A description of any assumptions or corrections, such as tests of normality and adjustment for multiple comparisons
☐	☒	A full description of the statistical parameters including central tendency (e.g. means) or other basic estimates (e.g. regression coefficient) AND variation (e.g. standard deviation) or associated estimates of uncertainty (e.g. confidence intervals)
☐	☒	For null hypothesis testing, the test statistic (e.g. *F*, *t*, *r*) with confidence intervals, effect sizes, degrees of freedom and *P* value noted<br>*Give P values as exact values whenever suitable.*
☒	☐	For Bayesian analysis, information on the choice of priors and Markov chain Monte Carlo settings
☒	☐	For hierarchical and complex designs, identification of the appropriate level for tests and full reporting of outcomes
☒	☐	Estimates of effect sizes (e.g. Cohen's *d*, Pearson's *r*), indicating how they were calculated

Our web collection on statistics for biologists contains articles on many of the points above.

Software and code

Policy information about availability of computer code

Data collection	No sofware was used for data collection (see section below).
Data analysis	EAGER (v1.92.59), AdapterRemoval (v2.2.0), BWA (v0.7.12), samtools (v1.3), dedup (v0.12.2), mapDamage (v2.0.6), Preseq (v2.0), CircularMapper (v1.93.5), Schmutzi, AuthentiCT(v1.0.0), ANGSD (v0.910), trimBam, pileupCaller, EIGENSOFT(7.2.1), ADMIXTOOLS (v5.1), DATES (v753), hapROH (v1.0), READ, lcMLkin, GATK (v3.5), GeneImp (v1.3), Haplogrep (v2.1.25)

For manuscripts utilizing custom algorithms or software that are central to the research but not yet described in published literature, software must be made available to editors and reviewers. We strongly encourage code deposition in a community repository (e.g. GitHub). See the Nature Portfolio guidelines for submitting code & software for further information.

Data

Policy information about availability of data

All manuscripts must include a data availability statement. This statement should provide the following information, where applicable:
- Accession codes, unique identifiers, or web links for publicly available datasets
- A description of any restrictions on data availability
- For clinical datasets or third party data, please ensure that the statement adheres to our policy

Sequencing data can be accessed through the European Nucleotide Archive (ENA) under project ID: PRJEB56216. Haploid genotype data for the 1240K panel in eigenstrat format will be also provided.

Field-specific reporting

Please select the one below that is the best fit for your research. If you are not sure, read the appropriate sections before making your selection.

☐ Life sciences ☐ Behavioural & social sciences ☒ Ecological, evolutionary & environmental sciences

For a reference copy of the document with all sections, see nature.com/documents/nr-reporting-summary-flat.pdf

Ecological, evolutionary & environmental sciences study design

All studies must disclose on these points even when the disclosure is negative.

Study description	This study employs ancient DNA laboratory protocols to produce genome-wide data from human skeletal remains and applies statistical tools from the field of population genetics to address questions regarding the population history such as admixture, genetic relatedness, demography and consanguinity.
Research sample	Human skeletal remains from the Aegean (present-day Greece) that were recovered from archaeological excavations.
Sampling strategy	The overall burial record from the Aegean Neolithic and Bronze Age is a corpus which underwent specific selection criteria in the past and has been subject to specific modes of preservation and excavation since then (e.g., only individuals with a certain status and/or age were buried in a way that allows their study at present). The corpus of samples analyzed in this study represents a broad variety of burial contexts (e.g., shaft graves/collective graves, single graves, primary and secondary burials) through time, and comes from areas with distinct features with respect to their archaeological history. The majority of the samples dates to the Bronze Age, an archaeological period at the core of our questions regarding the contacts of the populations with neighbouring regions and the social organization.
Data collection	Bone powder was sampled following minimally invasive methods for sampling of archaeological material. DNA was extracted converted into a genomic library with adaptors for sequencing on Illumina platforms.
Timing and spatial scale	Timing scale: Neolithic (ca. 6000 BC; n=6), Bronze Age (ca. 2800-1050 BC; n=95), Iron Age (ca. 900 BC; n=1)<br>Spatial scale: Southern Greek mainland, Aegean islands and Crete.
Data exclusions	Processed samples for which a very low coverage of genetic markers was generated (e.g., ≤40,000 SNPs), or modern DNA contamination was estimated high.
Reproducibility	Sequence data will be uploaded to the European Nucleotide Archive, and all parameters (e.g., mapping quality filters, genotyping methods, admixtools) are provided in the Method's section and Supplementary Note 2.
Randomization	No statistical methods were applied for the determination of sample size and randomization.
Blinding	Blinding was not relevant/possible for our study, since all the data come from archaeological samples.

Did the study involve field work? ☐ Yes ☒ No

Reporting for specific materials, systems and methods

We require information from authors about some types of materials, experimental systems and methods used in many studies. Here, indicate whether each material, system or method listed is relevant to your study. If you are not sure if a list item applies to your research, read the appropriate section before selecting a response.

Materials & experimental systems

n/a	Involved in the study	
☒	☐	Antibodies
☒	☐	Eukaryotic cell lines
☐	☒	Palaeontology and archaeology
☒	☐	Animals and other organisms
☒	☐	Human research participants
☒	☐	Clinical data
☒	☐	Dual use research of concern

Methods

n/a	Involved in the study	
☒	☐	ChIP-seq
☒	☐	Flow cytometry
☒	☐	MRI-based neuroimaging

Palaeontology and Archaeology

Specimen provenance	Access to the material was granted through different applications: for every archaeological site a separate application was submitted to the Greek Ministry of Culture and Sports, and after its approval, skeletal samples and/or bone powder could be exported to Germany. All permits were issued in Greek and copies could be provided upon request.

Specimen deposition	Samples in the form of small fragments (e.g., petrous bones, teeth) or bone powder (max. 200 mg) were exported and sent to the Max Planck Institute for the Science of Human History (MPI-SHH) Lab facility, in Jena, Germany.
Dating methods	Radiocarbon dating with Accelerator Mass Spectrometry on bone/tooth samples weighing up to 1g. Samples were sent to the Klaus-Tschira-Archäometrie-Zentrum at the CEZ Archaeometry gGmbH, in Mannheim, Germany and were analyzed on a MICADAS-AMS platform. Measurements were calibrated using the datasets IntCal13 and IntCal20 and the software SwissCal 1.0.

☒ Tick this box to confirm that the raw and calibrated dates are available in the paper or in Supplementary Information.

Ethics oversight	No ethical approval/guidance was required. All the material was accessed after permission from the Greek Ministry of Culture and Sports and the agreement of the institutions/researchers who studied archaeologically the material and who also agreed to participate in this study.

Note that full information on the approval of the study protocol must also be provided in the manuscript.
